# Evidence-Based Clinical Recommendations for the Appropriate Use of Diagnostic Tests in Pediatric Allergology: Focus on Asthma, Rhinoconjunctivitis, and Keratoconjunctivitis Vernal

**DOI:** 10.3390/jcm15124848

**Published:** 2026-06-22

**Authors:** Valentina Fainardi, Matteo Riccò, Rachele Antignani, Simona Bellodi, Claudia Borrelli, Tommaso Carretta, Mauro Calvani, Fabio Cardinale, Elena Chiappini, Maria Angiola Crivellaro, Massimiliano Esposito, Roberto Grandinetti, Amelia Licari, Michele Miraglia Del Giudice, Maria Marsella, Alberto Martelli, Iria Neri, Rita Nocerino, Diego Peroni, Cristina Piersantelli, Giuseppe Pingitore, Arianna Rossi, Giuseppe Squazzini, Mariangela Tosca, Carlo Caffarelli, Susanna Esposito

**Affiliations:** 1 Paediatric Clinic, Department of Medicine and Surgery, University of Parma, 43125 Parma, Italy; valentina.fainardi@unipr.it (V.F.); claudia.borrelli@unipr.it (C.B.); tommaso.carretta@unipr.it (T.C.); roberto.grandinetti@unipr.it (R.G.); arianna.rossi@unipr.it (A.R.); carlo.caffarelli@unipr.it (C.C.); 2 Servizio di Prevenzione e Sicurezza Negli Ambienti di Lavoro (SPSAL), AUSL-IRCCS Reggio Emilia, 42122 Reggio Emilia, Italy; mricco2000@gmail.com; 3 Primary Care Pediatricians, Società Italiana Medici Pediatri (SIMPE), Italy; r.antignani@virgilio.it (R.A.); simona.bellodi@gmail.com (S.B.); squazzo1957@gmail.com (G.S.); 4 Società Italiana di Allergologia e Immunologia Pediatrica (SIAIP), Italy; maurocalvani58@gmail.com (M.C.); fabiocardinale1961@gmail.com (F.C.); amelia.licari@unipv.it (A.L.); michele.miragliadelgiudice@unicampania.it (M.M.D.G.); diego.peroni@unipi.it (D.P.); mariangelatosca@gaslini.org (M.T.); 5 UOC Pediatria, Ospedale S. Camillo-Forlanini, 00152 Roma, Italy; 6 UOC Pediatria, Ospedale Pediatrico “Giovanni XXIII”, 70126 Bari, Italy; 7 UOC Malattie Infettive, Ospedale Meyer, 50139 Firenze, Italy; elena.chiappini@unifi.it; 8 Società Italiana di Pediatria Preventiva e Sociale (SIPPS), Italy; 9 UOSD Allergologia Azienda Ospedale Università di Padova, 35122 Padova, Italy; mariaangiola.crivellaro@aopd.veneto.it; 10 Società Italiana di Medicina Legale e delle Assicurazioni (SIMLA), Italy; massimiliano.esposito@unikore.it; 11 Faculty of Medicine and Surgery, “Kore” University of Enna, 94100 Enna, Italy; 12 UOC Clinica Pediatrica, IRCCS Policlinico San Matteo, 27100 Pavia, Italy; 13 Dipartimento della Donna, del Bambino e di Chirurgia Generale e Specialistica, Università della Campania Luigi Vanvitelli, 80131 Napoli, Italy; 14 Società Italiana Gestori del Rischio in Sanità (SIGeRiS), Italy; m.marsella79@gmail.com; 15 UOC di Pediatria Azienda Ospedaliera di Rilievo Nazionale San Giuseppe Moscati di Avellino, 83100 Avellino, Italy; 16 Network delle Associazioni di Allergologia Pediatrica, Italy; albmartelli@tiscali.it; 17 Società Italiana di Dermatologia Pediatrica (SIDeRP), Italy; iria.neri@aosp.bo.it; 18 UOC Clinica Dermatologica, IRCCS Azienda Ospedaliera Universitaria di Bologna, 40138 Bologna, Italy; 19 Società Italiana di Pediatria Infermieristica (SIPINF), Italy; rita.nocerino@unina.it; 20 Clinica Pediatrica, Università di Pisa, 56126 Pisa, Italy; 21 Società Italiana delle Cure Primarie Pediatriche (SICuPP), Italy; cristinapiersantelli@libero.it; 22 Società Italiana di Malattie Respiratorie Infantili (SIMRI), Italy; giuseppe.pingitore@gmail.com; 23 Centro di Allergologia, IRCCS Giannina Gaslini, 16147 Genova, Italy; 24 Società Italiana di Telemedicina (SIT), Italy

**Keywords:** pediatric allergology, evidence-based recommendations, asthma, rhinoconjunctivitis, keratoconjunctivitis Vernal, diagnostic testing, clinical appropriateness

## Abstract

**Background:** Appropriateness of diagnostic test prescriptions represents a critical component of quality care in pediatric allergology, directly influencing diagnostic accuracy, therapeutic decisions, healthcare resource utilization, and patient outcomes. A multidisciplinary expert panel was convened to develop evidence-based clinical recommendations addressing the appropriate use of specialist consultations and diagnostic investigations in children with asthma, allergic rhinoconjunctivitis, and vernal keratoconjunctivitis (VKC). **Methods:** Clinical questions were formulated using the PICO framework and prioritized through structured expert consensus. Systematic literature reviews were conducted across major databases, and the certainty of evidence was assessed using the GRADE methodology. **Results:** Specialist evaluation emerged as a key determinant of improved diagnostic precision, optimization of treatment strategies, and reduction of inappropriate therapies. In asthma, spirometry, FeNO measurement, and allergy testing contributed to enhanced diagnostic accuracy and better control. In allergic rhinoconjunctivitis, allergological assessment supported diagnosis and the selection of immunotherapy, with demonstrated benefits on symptoms and quality of life. For VKC, multidisciplinary specialist involvement facilitated early diagnosis, personalized management, and prevention of complications. **Conclusions:** Although the overall certainty of evidence ranged from moderate to low, consistent clinical benefits supported consensus-based recommendations. Implementation of these recommendations may improve care quality, promote equitable access to diagnostic resources, and reduce unnecessary healthcare utilization.

## 1. Introduction

The appropriate use of diagnostic and instrumental tests is essential to ensure equitable access to healthcare, optimize resource allocation, and improve the quality of care. Inappropriate prescribing, conversely, may result in unnecessary investigations, increased healthcare costs, prolonged waiting times, and avoidable burdens for patients, families, and healthcare professionals. Therefore, the development of clear and shared recommendations based on the best available scientific evidence is crucial to promote high-quality care and support the sustainability of healthcare systems [1].

Given the substantial burden of allergic diseases in childhood and adolescence, evidence-based Good Clinical Practice Recommendations (GCPRs) may play a particularly important role in pediatric allergology, especially in the management of conditions such as asthma, allergic rhinoconjunctivitis, and vernal keratoconjunctivitis (VKC) [2,3,4,5].

The development of effective and up-to-date GCPRs requires several key methodological principles, including: (1) clarity in the definition of each attribute; (2) compatibility of each attribute and its definition with professional usage; (3) clear rationales and justifications for the selection of each attribute, and the (4) sensitivity to practical issues in using the attribute to assess actual sets of practice guidelines (i.e., “accessibility”). When these principles are adequately addressed, GCPRs can support validity, reproducibility, clinical applicability, flexibility, and multidisciplinary integration, ultimately facilitating their implementation in routine practice.

The aim of the work was to develop shared recommendations based on the best available evidence to reduce variability in diagnostic prescribing practices, harmonize clinical management at a national level, and improve equity in access to healthcare services for children and adolescents within the following themes: asthma, rhinoconjunctivitis, and vernal keratoconjunctivitis (VKC).

## 2. Materials and Methods

### 2.1. Working Group and Panel of Experts

The present document is the result of the joint work of a multidisciplinary panel, which includes representatives of pediatrics scientific societies (Italian Society of Pediatricians [Società Italiana Medici Pediatri, in Italian; SIMPE], Italian Society of Pediatrics [Società Italiana di Pediatria, in Italian; SIP], Italian Society of Pediatric Allergology and Immunology [Società Italiana di Allergologia e Immunologia Pediatrica, in Italian; SIAIP], Italian Society for Childhood Respiratory Diseases [Società Italiana per le Malattie Respiratorie Infantili, in Italian, SIMRI], Italian Society of Pediatric Emergency Medicine [Società Italiana di Medicina di Emergenza e Urgenza Pediatrica, in Italian; SIMEUP], Italian Society of Pediatric Primary Care [Società Italiana delle Cure Primarie Pediatriche, in Italian; SICuPP]); representatives of scientific societies of specialists involved in the care of allergic children (Italian Society of Pediatric Dermatology [Società Italiana di Dermatologia Pediatrica, in Italian; SIDerP], Italian Association of Local and Hospital Allergists and Immunologists [Associazione Allergologi Immunologi Italiani Territoriali e Ospedalieri, AAITO]), as well as experts in legal medicine from the Legal and Insurance Medicine (Società Italiana di Medicina Legale e delle Assicurazioni, in Italian; SIMLA), and in telemedicine from Italian Telemedicine Society, (Società Italiana di Telemedicina, in Italian; SIT).

To guarantee the multidisciplinary nature of GCPR on the topics of asthma, rhinoconjunctivitis, and VKC, a multi-professional working group was designed and included pediatric allergologists, general pediatricians, general practitioners, clinical immunologists, otolaryngologists, dermatologists, clinical pharmacologists, psychologists, and pediatric nurses. The group also had methodologists and representatives of patient associations to respond to the concrete needs of the families. This study was part of a broader initiative aimed at producing evidence-based GCPR for prescriptions in pediatric patients with allergic diseases convened under the Italian Superior Institute of Health (Istituto Superiore di Sanità, in Italian; ISS).

### 2.2. Formulation of Clinical Questions

As a preliminary step, clinical questions addressing the diagnostic management of asthma, rhinoconjunctivitis, and VKC in children and adolescents were identified and formulated by the working group according to the model population/patient (P), intervention (I), comparator (C), outcome (O), or PICO [6]. Each member of the working group was invited to propose up to five PICOs for each topic. All proposed questions were subsequently gathered, reviewed and prioritized by experts by means of a 9-point LIKERT scale (0–3 = disagreement, 4–6 = moderate agreement, 7–9 = strong agreement). Only PICOs with an average score greater than 7 were included in the subsequent evidence review and recommendation development process.

### 2.3. Systematic Review

To address the clinical questions approved by participating experts, a series of systematic literature reviews was conducted (see Prisma Checklist in Supplementary Material Table S1). Three different databases (Medline, Embase, and Cochrane) were searched, exploring a combination of keywords related to allergy, atopy, allergic disease, and condition-specific terms; complete search strategies are reported in Appendix A Table A1.

The following inclusion criteria were applied:

- Study design: randomized controlled trials (RCTs), cohort studies with a comparator, and case–control studies were included. Systematic reviews, with or without meta-analyses and clinical guidelines, were initially retrieved and then screened to identify additional relevant studies through a snowball approach. Case reports, letters to the editor, brief reports, and conference abstracts were excluded;
- Population: children and adolescents, i.e., subjects aged 0–18 years;
- Language and publication period: studies published in English, published between January 2015 and January 2025.

Study selection was conducted according to the Preferred Reporting Items for Systematic Reviews and Meta-Analyses (PRISMA) statement [6,7]. As a preliminary step, all suitable articles were initially pooled together. After duplicate removal, two reviewers independently screened titles and abstracts. Full-text assessment of potentially eligible articles was subsequently performed by the same reviewers. Disagreements at any stage were resolved through discussion and, when necessary, consultation with a third researcher.

The methodological quality of observational studies was assessed by means of the Newcastle–Ottawa scale (NOS, potential range from 0 to 9 points), which has been extensively adopted for risk-of-bias assessment in systematic reviews [8,9]. The NOS consists of four domains of risk of bias assessment: (1) selection bias; (2) performance bias; (3) detection bias; and (4) information bias. Although originally developed for cohort and case–control studies, the cohort version of the NOS was also applied to cross-sectional studies because of the substantial methodological similarities between these designs [10]. According to the current indications and the study protocols, two investigators independently rated all suitable articles and provided a summary of their potential shortcomings. Potential disagreements were primarily resolved by consensus between the two reviewers or input from a third investigator. Studies were classified as low (0–3 points), moderate (4–6 points), and high quality (7–9 points). Study quality was not used as an exclusion criterion. Instead, all studies meeting the predefined eligibility criteria were retained to ensure a comprehensive assessment of the available evidence. Methodological limitations were subsequently incorporated into the overall certainty of evidence through risk-of-bias assessment and the GRADE framework.

For RCT, risk of bias was evaluated by means of the ROB tool from the National Toxicology Program (NTP)’s Office of Health Assessment and Translation (OHAT) (now the Health Assessment and Translation (HAT) group) [11,12]. OHAT ROB provides a 40-point scale rating (from “definitely low”, “probably low”, and “probably high” to “definitely high”) on the following potential sources of bias: participant selection (D1), confounding factors (D2), attrition/exclusion (D3), detection (D4), and selective reporting (D5), as well as other sources of bias (D6). OHAT ROB was prioritized over other instruments for ROB assessment, as it does not provide an overall rating for each study, nor does it require that studies affected by a certain degree of ROB be removed from the pooled analyses [11].

### 2.4. Data Extraction

Studies meeting the predefined eligibility criteria were included in the data extraction process. Extracted information included: study title, authors, journal, year of publication, target population (disease and age group), intervention and comparator characteristics, outcomes, and main findings. Whenever available, numerical data were extracted to allow quantitative synthesis through a meta-analysis approach. In the absence of sufficient numerical information, findings were summarized narratively. Data extraction was performed with the aim of supporting both quantitative and qualitative evidence synthesis according to the characteristics of the available studies.

### 2.5. Meta-Analysis

When at least five studies evaluated the same type of intervention in the same population and reported the same outcome, a meta-analysis was conducted. For dichotomous data, if derived from randomized controlled trials, Relative Risk (RR) with a 95% confidence interval (95%CI) was used; for observational studies, Odds Ratio (OR) with a 95% CI was used. For continuous variables, the mean difference or standardized mean difference was used if different scores were used to evaluate the same outcome. These analyses were conducted when the mean, standard deviation, and sample size of each group were available. If the mean and standard deviation were not directly available but other data, such as the confidence interval, were available, the standard deviation was imputed from the available data. If data were not available, an email was sent to the corresponding author requesting the data to include the study in the meta-analysis.

Statistical analyses were performed by means of IBM SPSS Statistics 26.0 for Macintosh (IBM Corp. Armonk, NY, USA), GraphPad Prism Version 10.6.1 (799) (GraphPad Software LLC, San Diego, CA, USA), R (version 4.4.1) [13] and RStudio 2026.01.0 Build 392 (Posit Software PBC, Boston, MA, USA) by means of the packages fmsb (version 0.7.6), ggplot2 (version 4.0.1), ggpubr (version 0.6.2), and meta (version 8.2.1).

### 2.6. GRADE and GRADE Framework

The certainty of evidence was assessed using the GRADE (Grading of Recommendations Assessment, Development and Evaluation) methodology [14,15]. For each PICO, relevant outcomes were identified and grouped according to the intervention under evaluation. For each outcome, the certainty of evidence was assessed by considering study design, risk of bias, inconsistency of results, indirectness, imprecision, and potential publication bias. In addition to evaluating the certainty of evidence, recommendations were developed using the GRADE Evidence-to-Decision (EtD) framework. For each intervention, the panel considered multiple domains, including the magnitude of the problem, desirable and undesirable effects, certainty of evidence, patient values and preferences, resource requirements, certainty of evidence regarding resource use, cost-effectiveness, equity, acceptability, and feasibility. Recommendation strength was therefore determined not solely by the certainty of evidence, but by integrating all EtD domains. Consequently, strong recommendations could be formulated despite moderate certainty of evidence when the balance of benefits and harms was considered clearly favorable, implementation was feasible, and stakeholder acceptability was high.

### 2.7. Consensus Panel for the Strength of Recommendations

To reach a consensus on the strength of the recommendations, a survey was conducted among the expert panel. Once the recommendations were formulated, a survey was sent to each member of the panel. Using a Likert scale from 0 to 9, each expert was asked to express their opinion on the strength of the recommendation (0–3 low strength, 4–6 moderate, 7–9 strong) and on sustainability (0–3 not sustainable, 4–6 probably sustainable, 7–9 certainly sustainable). The average scores given by each expert were then used to define the strength of the recommendations. In case of highly contradictory responses, the survey results were discussed among the expert panel.

The development process of the recommendations was in accordance with the standards defined by the document “Methodological guidelines for drafting recommendations for good clinical and care practices” [16].

## 3. Results

The risk-of-bias assessment of each study and the quality of evidence, evaluated with GRADE methods and with the GRADE/GRADE EtD Framework, the description of the included studies, and the search strategies are shown in the Supplementary Materials Table S1.

### 3.1. Asthma

#### 3.1.1. Summary of Literature Search

**Figure 1:**
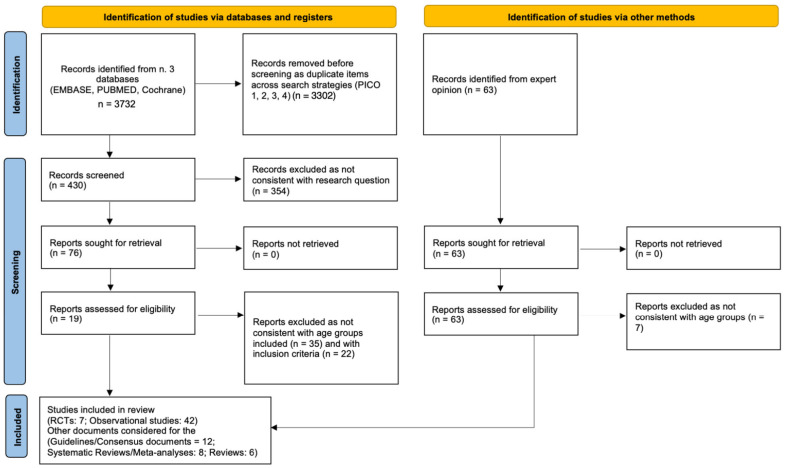
Flowchart of studies on asthma [6].

The literature research across Embase, Medline and the Cochrane Library identified 3732 articles. After duplicate removal (*n* = 3302, 88.5%), a total of 430 studies underwent title and abstract screening (11.5%). After the removal of articles considered not consistent with research questions (*n* = 354, 9.5%), a total of 76 articles were sought for retrieval (2.0%). Of them, 19 (0.5%) were included in the final analyses, while 35 articles were excluded as they were not consistent with the age groups included in the analyses (0.9%), and 22 were inconsistent with the inclusion criteria (0.6%). In addition, 63 articles were identified by the expert panel as potentially relevant sources of evidence. Of them, seven were then excluded as they were not consistent with the assessed age groups. Overall, the study selection process (Figure 1) led to the inclusion of 75 studies, comprising 42 observational studies, 7 RCTs, 12 clinical guidelines, 8 systematic reviews, and 6 narrative reviews.

The detailed list of the studies included in the present document is reported and described in Appendix A Table A2. Their corresponding quality appraisal is summarized in Appendix A Table A3 and Table A4.

#### 3.1.2. Clinical Questions

The full list of inquired PICOs is summarized in Table 1.

##### PICO 1

In children and adolescents with uncontrolled allergic asthma, is a pediatric allergy or pediatric pulmonology visit recommended compared to only management by the primary care pediatrician to improve therapy adherence and disease control?

According to this specific question, the main outcomes considered included: the level of asthma control, measured through standardized questionnaires; adherence to therapy, measured through standardized questionnaires or the use of devices; exacerbations; and diagnostic accuracy, measured through spirometry, bronchial FeNO, and allergy tests.

Evidence (Narrative report). Uncontrolled asthma is frequently associated with the excessive use of short-acting beta2 agonists (SABA), which has been linked to an increased risk of exacerbations, healthcare utilization, and asthma-related mortality [17]. Evidence from a large cohort study involving adolescents and adults with asthma demonstrated a significant association between high SABA consumption and increased rates of asthma exacerbations, hospitalizations, outpatient visits, and oral corticosteroid usage. Furthermore, the study observed a corresponding increase in asthma-related mortality, which rose proportionally with the number of SABA inhalers dispensed annually [18].

Several studies have identified frequent use of bronchodilators, discontinuity in medical care, poor inhalation technique, non-adherence to therapy, and delayed specialist referral as the major contributors to asthma exacerbations and poor asthma control [19,20,21,22,23,24]. In a cohort of children with severe asthma in the Scandinavian Peninsula, half were managed solely by primary care physicians [22], and in a large cohort of English children and adolescents with poorly controlled asthma, only 2% had been referred to specialist care [2,25]. These data highlight the need for greater awareness of the risks associated with poor asthma control and the need for increased management of uncontrolled asthma by respiratory specialists.

In the United Kingdom, where asthma patients have a higher risk of exacerbation and asthma-related death compared to other high-income European countries, a pilot asthma management hub was created to facilitate visits for patients with uncontrolled asthma or those who had experienced asthma attacks. Access to specialist care increased asthma diagnoses and allowed for thorough specialist visits with spirometry and bronchodilator reversibility tests (BDR), FeNO measurement, and asthma management education, resulting in the identification of individuals at risk of asthma attacks and therefore deserving of maintenance treatment [25].

Paskaradevan et al. [26] observed a group of asthmatic patients aged 4 to 18 years old admitted to pediatric intensive care for asthma attacks. Among patients who had a pneumological consultation during hospitalization (44.7%), there was a higher likelihood of obtaining an increase in maintenance therapy (66% vs. 21%) and a further visit in the clinic as outpatients compared to patients who did not have the consultation [26,27].

Specialist evaluation in asthmatic children can improve disease progression and influence the use of daily and as-needed therapy. As demonstrated by Rosman et al., after a specialist visit, the average number of emergency room visits and hospitalizations for asthma exacerbations decreased, as did the prescription of SABA and oral corticosteroids. However, the prescription of inhaled corticosteroids as maintenance therapy increased [28]. Other authors have also shown that when children hospitalized for asthma symptoms received a specialist visit within 14 days of the acute event, they had fewer emergency room visits for asthma in the following 60 and 365 days [29].

Despite guidelines recommending the daily use of maintenance inhalers in uncontrolled asthma, the daily use of inhaled corticosteroids (ICS) varies from 40% to 70% [30,31]. Factors associated with the lack of daily preventive asthma treatment include the absence of a specialist in asthma management and the follow-up by primary care physicians [32,33,34,35,36,37]. The literature indicates that specialists are more likely to prescribe inhaler maintenance therapy compared to primary care physicians [37,38]. The lack of instruments and time may contribute to the lower tendency of primary care physicians to diagnose asthma and prescribe daily treatment. Data indicate that adequately trained specialists need about 50 min to explore the various aspects of the disease, a significantly longer time than the average duration of a primary care visit [25,39]. Additionally, specialists in pulmonology are more experienced in managing patients with uncontrolled and severe asthma [39].

Assessment of lung function with spirometry, measurement of allergic bronchial inflammation with exhaled nitric oxide fraction (FeNO), prick tests for allergens and the measurement of blood eosinophils are integral parts of the management of patients with uncontrolled allergic asthma to confirm the diagnosis and initiate the most appropriate treatment [2,25,38,39,40,41,42].

A considerable part of the specialist visit is also dedicated to asthma education, including the management of acute attacks and inhalation technique. Recent systematic reviews of 26 RCTs have shown that educational interventions for asthma can improve not only patients’ quality of life but also reduce hospitalizations [43,44]. In addition to inhalation technique, adherence to therapy plays a prominent role in good asthma control, but this can be particularly low, especially in adolescents [43,44]. Data on the association between adherence and specialist management are controversial: Keemink et al. found that long-term adherence to daily maintenance therapy did not change before and after a scheduled follow-up visit, while Koole et al. demonstrated that providing personalized information based on the needs of the patient and caregiver improves long-term treatment adherence and agreement with the proposed therapy [45,46]. Poor asthma control can be attributed in most cases to modifiable factors, such as adherence to maintenance therapy [47]. In a retrospective study of 142 children and adolescents with uncontrolled asthma, 97.2% had underlying causes that could explain poor control [43]: 37.3% had poor adherence to maintenance therapy, 28.2% were constantly exposed to allergens to which they were sensitized, 19.7% had associated comorbidities, 7.7% used the inhaler incorrectly, and 4.2% did not have asthma but another disease. Only a specialist evaluation could identify these factors, which, once modified and improved, resulted in good asthma control and normal respiratory function in all children in the cohort [48,49,50,51].

Recommendation (Table 2). Due to the moderate quality of evidence, a consensus-based recommendation rather than an evidence-based one was framed. Briefly, in patients with uncontrolled asthma, a specialist evaluation is recommended to confirm asthma diagnosis, improve treatment adherence and asthma control, and reduce exacerbations.

##### PICO 2

In children and adolescents with suspected allergic asthma, do allergy tests (such as the skin prick test and measurement of specific IgE) offer greater effectiveness than clinical assessment alone in identifying triggers and improving disease control?

Evidence (Narrative report). Most children with asthma are atopic [35,50]. The Th2-high phenotype is characterized by eosinophilic airway inflammation, with increased levels of IL-4, IL-5 and IL-13 produced by both innate and adaptive immune cells. These cytokines promote eosinophil and mast cell activation and stimulate IgE production [49]. Biomarkers of Th2 inflammation include FeNO, blood eosinophil count, positive prick tests for allergens, and total and specific serum IgE [48]. Although these biomarkers may support the diagnosis of asthma, particularly when lung function tests are normal or inconclusive, a positive prick test or positive specific IgE for an allergen does not necessarily mean that that allergen is the trigger for asthma. The clinician should integrate these results with the patient’s clinical history to make a diagnosis [2]. On the other hand, in children with suspected asthma and negative prick tests or normal total IgE, the diagnosis of asthma is unlikely and should be confirmed with other tests, such as the bronchial provocation test. Some children can have non-allergic eosinophilic asthma, with peripheral eosinophilia in the absence of allergic sensitization to common environmental allergens [25]. One of the causes of uncontrolled allergic asthma is constant exposure to allergens [52], with almost 70% of children hospitalized for asthma sensitized to three or more allergens present in the home environment [35]. Routine allergy tests help to identify the phenotype of asthma (allergic eosinophilic, non-allergic eosinophilic, neutrophilic) and can guide specific therapy, such as immunotherapy or biologics in cases of severe asthma. Furthermore, the evidence of sensitization justifies allergen avoidance interventions, for example, in the home environment, resulting in reduced morbidity in patients at risk of exacerbation [49,53]. An RCT involving 434 children with allergic asthma demonstrated that the use of anti-mite mattress covers significantly reduced asthma exacerbations in children sensitized to dust mite [54,55]. In children sensitized to dust mite with allergic rhinitis and controlled asthma, sublingual immunotherapy seems to reduce symptoms, although evidence is still limited [56,57].

A subtype of asthma that can benefit from allergy tests is fungal sensitization asthma (SAFS). SAFS is predominantly mediated by IL-33 [49] and is clinically more severe and more resistant to steroids. Fungal sensitization can be demonstrated by positive prick tests or specific IgE towards at least one of the following seven fungi (*Aspergillus fumigatus*, *Alternaria alternata*, *Cladosporium herbarum*, *Penicillium chrysogenum*, *Candida albicans*, *Trichophyton mentagrophytes*, or *Botrytis cinerea*), in the absence of criteria for allergic bronchopulmonary aspergillosis (ABPA). These patients are generally more atopic, older, have higher serum IgE levels, which is associated with severe asthma and exacerbations [58,59,60], and are often treated with oral steroids [61].

A recent Swiss study evaluated the diagnostic accuracy of respiratory symptoms, spirometry, plethysmography, FeNO, prick tests, and IgE, as well as the most commonly used diagnostic algorithms proposed by GINA and the National Institute for Health and Care Excellence (NICE) for diagnosing asthma in children referred to a specialist for suspected asthma. Among the 514 subjects included in the study, 70% had asthma. The highest sensitivity and specificity were demonstrated by the presence of wheezing in clinical history, by the measurement of airway resistances through plethysmography, by the response to bronchodilator and by positive prick tests, which were very sensitive but not very specific [62].

A recent meta-analysis performed within the latest guidelines of the British Thoracic Society, which included three studies conducted in pediatric age [63,64,65], showed that the specificity and sensitivity of specific IgE and prick tests in diagnosing asthma were higher for dust mites and that these tests were especially useful in combination with other exams to diagnose suspected asthma. The NICE guideline on food allergy indicates that prick tests require 40 min to be performed and that the final cost for each test is 44.95 pounds [25]. The work emphasizes that this test is sustainable within a specialist diagnostic algorithm.

Recommendation (Table 3). Due to the heterogeneous design of included studies that ultimately impaired a quantitative summary of evidence, a consensus-based recommendation rather than an evidence-based one was framed. Children and adolescents with suspected allergic bronchial asthma can benefit from allergy tests (prick tests and specific IgE measurements) to confirm the diagnosis, especially in uncertain cases, identify subjects at risk of exacerbation, recognize asthma triggers, and guide specific interventions and treatments.

##### PICO 3

For children and adolescents with allergic asthma, does spirometry with bronchodilator reversibility improve diagnostic accuracy and asthma control compared to clinical evaluation alone?

Evidence (Narrative report). Spirometry with bronchodilator testing confirms reversible airway obstruction, an essential element to diagnose asthma in children [2,25,66]. In the diagnostic algorithms of the main asthma recommendations and guidelines, spirometry with bronchodilation represents one of the fundamental tools for establishing a diagnosis. Especially in pediatric age, respiratory symptoms can be nonspecific and sometimes caused by viral respiratory infections. An incorrect diagnosis can lead to excessive and unjustified treatment with inhaled corticosteroids or, conversely, to insufficient treatment [67]. However, data show that only one-third of children undergo spirometry in the year before or two years after the diagnosis of asthma [68].

In children, spirometry can be normal even in the presence of asthma. A response to bronchodilator with an increase in FEV1 ≥ 12% or >200 mL confirms the diagnosis of asthma and supports the importance of respiratory function tests even in apparently healthy children [66]. A recent meta-analysis of the literature (11), which included only one RCT conducted in pediatric age, showed that using a cut-off of 88.4% in the FEV1 value, spirometry had moderate sensitivity (0.68) and specificity (0.76). The study, which involved 275 children, suggested that FEF 25–75 may be the best value for diagnosing asthma with an area under the curve (AUC) of 0.81 (95% CI: 0.76–0.87), higher than for FEV1 [69]. No studies have evaluated the diagnostic accuracy of bronchodilator responsiveness in children.

Low lung function values can indicate an increased risk of exacerbation, as demonstrated by a cross-sectional study [70] in which the hospitalization rate for asthma was higher in the group with a lower FEV1/FVC ratio, regardless of asthma control and maintenance therapy. In addition, a marked bronchodilator response has been associated with higher risk of exacerbation [71].

Yimlamai S. et al. [72], in a retrospective study of 203 children with asthma with an average age of 10 years, observed that therapy adjustments based on symptoms or spirometry had the same outcome in terms of symptom control in the subsequent 12 months. An open-label RCT in 106 children with asthma showed that in the group where spirometry was used in addition to clinical evaluation, it influenced physicians’ decisions (modification of the diagnosis, assignment of severity, initiation of new therapies, request for investigations, decisions on the timing of the next follow-up), contributed to better disease control, and improved quality of life in relation to asthma symptoms [73].

Recommendation (Table 4). Due to the heterogeneous design of included studies that ultimately impaired a quantitative summary of evidence, a consensus-based recommendation rather than an evidence-based recommendation was framed. In children and adolescents with allergic asthma, spirometry with bronchodilator reversibility testing is recommended over clinical evaluation alone. This approach improves diagnostic accuracy, guides therapeutic decisions, and contributes to better disease control (Table 4).

##### PICO 4

In children and adolescents with suspected allergic asthma, spirometry combined with the measurement of exhaled nitric oxide (bronchial FeNO) is more useful than spirometry alone to improve diagnostic accuracy and guide therapeutic decisions.

Evidence (Narrative report). Nitric oxide is generated in the airways in response to type 2 inflammation, particularly IL-13 [74,75]. It correlates with eosinophils both in lung biopsies and in induced sputum [76]; it is considered a marker of allergic asthma. The major guidelines of the European Respiratory Society (ERS) [66,77] recommend the use of FeNO combined with other tests for the diagnosis of allergic asthma in children aged 5 to 16 years old, with a suggested threshold value of 25 ppb. The recent joint BTS/NICE/SIGN guidelines, considering the economic costs of diagnostic investigations, indicate for the first time that FeNO measurement should be performed as the first test in children with a clinical history suggestive of asthma, even before spirometry or allergy tests. A FeNO value of 35 ppb or higher is considered indicative of eosinophilic airway inflammation and supports the diagnosis of asthma with high sensitivity, while a value below 35 ppb, or the unavailability of the test, indicates that spirometry with bronchodilator reversibility testing should be used [25].

In addition to diagnosis, FeNO can be useful for measuring and monitoring allergic airway inflammation and for guiding therapy [78]. FeNO values can provide information on asthma severity and can also reflect the total serum IgE levels [79]. Moreover, since it is strongly influenced by ICS, it is particularly useful in assessing adherence to therapy. In a prospective observational study conducted in children aged 5 to 16 years with suspected or previous diagnosis of asthma, 54% of children who reported good asthma control still had abnormal spirometry and/or elevated FeNO, suggesting that symptom evaluation alone is not sufficient to identify children with airway obstruction or active allergic inflammation. After a complete visit that included spirometry and FeNO, a reduction in asthma attacks and an improvement in asthma control were observed during the subsequent six months [80,81].

Chen L. et al. [82], in a study on children belonging to ethnic minorities, found that intermediate and high FeNO levels were associated with lower airway obstruction and higher hospitalization rates. In a retrospective study on school-aged children with asthma [44], elevated FeNO values were long-term predictors of loss of asthma control. A 2016 literature meta-analysis that included nine RCTs conducted in pediatric age patients concluded that modifying asthma maintenance therapy based on FeNO value reduced exacerbations but not symptoms or the dose of inhaled steroids [83]. However, in a recent RCT on 506 children, the use of FeNO to guide therapeutic choice did not show a reduction in exacerbations compared to the standard of care, i.e., deciding therapy based on reported symptoms [84].

In two RCTs published after the 2016 meta-analysis, compared to children treated only with SABA, those treated with ICS showed significant reductions in FeNO, eosinophils in induced sputum, and IgE levels and had improvement in asthma control test scores [85,86]. Regarding feasibility, a prospective observational cohort study conducted in the UK [80,81] demonstrated that after adequate training, spirometry and FeNO measurement were feasible in most children aged 5 to 16 years old. The cost estimated by a recent literature meta-analysis varies from 6 to 11 pounds per measurement and the procedure requires approximately 15 min to perform. Considering the available economic analyses, the cut-off values reported in the literature, and comparisons with bronchodilator reversibility testing, the latest guidelines indicate a threshold of >35 ppb as providing the highest diagnostic accuracy. When FeNO measurement is combined with spirometry, both sensitivity and specificity for the diagnosis and management of allergic bronchial asthma in children increase [70,87,88].

Recommendation (Table 5). Due to the design of the research question, no quantitative analysis was performed and a consensus-based recommendation rather than an evidence-based recommendation was framed. In summary (Table 5), FeNO can be useful for identifying allergic inflammation and adherence to therapy. When combined with spirometry and bronchodilator testing, it improves diagnostic accuracy and helps guide treatment decisions in both allergic and non-allergic eosinophilic asthma.

### 3.2. Rhinoconjunctivitis

#### 3.2.1. Summary of Literature Search

A detailed description of the research questions is reported in Table 6.

**Figure 2:**
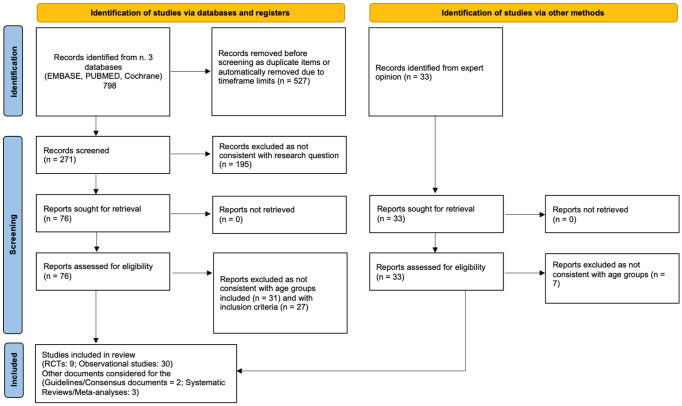
Flowchart of studies on rhinoconjunctivitis, PICO 1. See Appendix A Table A1 for the detailed search strategy [6].

**Figure 3:**
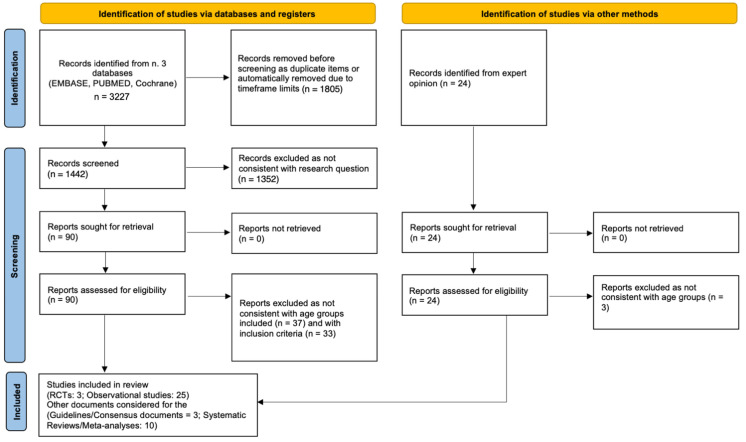
Flowchart of studies on rhinoconjunctivitis, PICO 2 and 3. See Appendix A Table A1 for detailed search strategy [6].

Due to the characteristics of targeted populations, we arbitrarily handled PICO 1 on the one hand, and PICO 2 and 3 on the other hand by means of two different flows of data, as depicted in Figure 2 and Figure 3.

As for PICO 1, the search across three databases (Embase, Medline, Cochrane) identified a total of 798 articles (Figure 2). After removing duplicates (*n* = 527, 66.0%), 271 studies were screened by title and abstract (34.0%). Of these, 195 were removed as not relevant to the underlying research question (24.4%). The remaining 76 articles were assessed by full text (9.5% of the parent sample): of them, 31 were removed as the participants’ age groups did not meet the inclusion criteria (3.9%), and 27 because they did not meet the predefined inclusion criteria (3.4%). The remaining 18 articles were included in the final analyses (2.3%). Moreover, based on expert opinion, 33 additional articles were added, for a total of 51 articles included in the analyses. Of them, 9 were RCTs, 30 were observational studies, 3 were systematic reviews with and without meta-analyses, and 2 were guidelines and/or consensus documents. The detailed characteristics of individual studies are reported in Appendix A Table A2, while their quality appraisal is reported in Appendix A Table A5 and Table A6.

Regarding PICO 2 and 3, the search across three databases (Embase, Medline, Cochrane) identified 3227 articles. After removing duplicates (*n* = 1805, 55.9%), 1422 studies were screened by title and abstract (44.1%). Of these, 90 articles were eventually reviewed in full text (2.8%): after assessment for relevance to the research question, 20 articles were included in the final analysis (0.6%). Based on expert opinion, 21 additional articles were added, for a total of 41 articles included. Of them, 25 were observational studies, 3 were RCTs, and the remaining studies consisted of systematic reviews with/without meta-analysis (*n* = 10) and guidelines/recommendations (*n* = 3).

The characteristics of the included studies are provided in Appendix A Table A2, while the detailed quality appraisal of retrieved studies is summarized in Appendix A Table A5 and Table A6.

#### 3.2.2. Clinical Questions

##### PICO 1

In children and adolescents with mild allergic rhinoconjunctivitis that does not respond to first-line therapy (topical nasal corticosteroids and/or oral antihistamines) or moderate/severe allergic rhinoconjunctivitis, is a consultation with a pediatric allergy specialist recommended over routine follow-up with a general pediatrician to identify the causative agent, initiate specific immunotherapy, and improve the quality of life?

Evidence (Narrative report). Allergy specialist consultation is a key element in the diagnosis, treatment, and improvement of quality of life in patients with mild allergic rhinoconjunctivitis that does not respond to first-line therapy with topical steroids or antihistamines, and in moderate/severe rhinoconjunctivitis. Numerous studies have shown that accurate identification of the causative agent, through the various available diagnostic methods, is essential to define an effective and manageable therapeutic plan [89,90].

A randomized trial conducted on 335 children aged 6 to 16 years with rhinitis and/or asthma evaluated the usefulness of the allergological intervention, reporting an overall improvement in symptoms and quality of life in terms of reduced use of health services, fewer sick days, and self-reported symptoms after 12 months [91]. In the attempt to improve the accuracy of allergic rhinoconjunctivitis diagnosis, several recommendations have proposed algorithms that include prick skin tests, specific IgE (sIgE) measurements, and molecular analyses [92]. However, these should be interpreted in the clinical context.

In an observational study conducted in a cohort of 758 children and adolescents aged 6 to 18 years with rhinitis, 18.4% had a negative skin test for sensitization and were initially classified as having non-allergic rhinitis (NAR). Of these, 25 patients were then subjected to nasal allergen challenge (NAC) with *Dermatophagoides pteronyssinus* and *Blomia tropicalis*, and 40% showed reactivity to at least one of the allergens, thus falling into the category of local allergic rhinitis (LAR) [93]. This distinction, difficult to clinically detect, underscores the importance of specialist evaluation to increase the likelihood of correct diagnostic classification.

A comparative study between the skin prick test (SPT) and sIgE in diagnosing sensitization to dust mites (*Dermatophagoides pteronyssinus*, *Dermatophagoides farinae*) and German cockroach (*Blattella germanica*) in 101 patients with asthma and/or allergic rhinitis showed that SPT had good specificity and positive predictive value for all allergens, but variable sensitivity, particularly low for dust mites, while sIgE showed good positive predictive value but lower sensitivity.

In a recent study evaluating the accuracy of SPT and sIgE levels in predicting dust mite-induced allergic rhinitis in 245 children with chronic rhinitis, a mean wheal diameter ≥ 6.6 mm on SPT and an sIgE value ≥ 17.0 kUA/L showed excellent specificity and positive predictive value. Nasal provocation (NPT) confirmed the diagnosis in 71.8% of patients [94]. The diagnosis and identification of the causative allergen must therefore rely on the expertise of an allergy specialist who can interpret the tests. A correct diagnosis can also help to prescribe targeted therapy to improve the patient’s quality of life, often compromised by persistent symptoms [95].

The guidelines of the European Academy of Allergy and Clinical Immunology (EAACI) emphasize that immunotherapy is an effective therapeutic option as it not only reduces allergic symptoms and the use of symptomatic drugs but also offers long-term benefits by helping to prevent progression to more severe conditions such as asthma [3].

A 2024 study conducted on 944 Italian patients showed that only 53.8% were aware of allergen-specific immunotherapy (ITS), and only a third had received this option as a therapeutic proposal. Among the patients who had completed ITS, all reported significant benefits, including reduced days of absence from work or school [96]. The benefits of ITS on allergic rhinitis symptoms have been widely demonstrated for both sublingual immunotherapy (SLIT) and subcutaneous immunotherapy (SCIT) [97].

Several studies have highlighted the impact of allergic rhinitis on quality of life (QoL) and the subsequent improvement after the introduction of immunotherapy. Using specific questionnaires such as the RQLQ (Rhinoconjunctivitis Quality of Life Questionnaire) for adults and the mini-RQLQ for children, nasal, ocular, sleep disturbances, emotional aspects, and difficulties in daily activities were evaluated [91]. On average, there is a 60% reduction in ocular and nasal symptoms with a high level of tolerance and adherence to pharmacological treatment [91,98,99]. In the 2019 ICARA prospective study, after 12 months of treatment, sleep quality increased by 56%, practical activities by 33.9%, general symptoms improved by 20.4%, and emotional state by 47.5% [100].

Proctor et al. reported an improvement in QoL of more than 50% within a year of therapy, whether it was for dust mite or seasonal pollen [101]. In Spain, patient satisfaction was assessed using the ESPIA scale and was estimated at around 69.2%, consistent with an efficacy rate of around 66% [102]. In a trial involving 399 patients with moderate/severe oculorhinitis, including 163 children, SLIT for grasses for three years showed a significant improvement in QoL both during the pollen season and in other seasons (24).

A 2023 prospective study analyzed 158 participants aged 5 to 16 years undergoing subcutaneous immunotherapy for pollens (birch and/or grasses). HRQoL (Health Related Quality of Life) after three years of therapy improved from a baseline score of 79.5 to 87.2. The symptom score decreased from 19.9 to 11.5 after just one year of treatment, maintaining this lower score in the second and third years (11.9 and 10.3, respectively). Severe symptoms decreased from 35.6% to 4.5% after one year of treatment [103,104]. In another trial evaluating specific sublingual immunotherapy for D. Pteronyssinus and B. Tropicalis versus placebo, despite the reduction in antihistamine (loratadine) use from 78.1% to 15.6% in the treated group compared to the placebo group, there were no changes in QoL [104]. The use of immunotherapy, as in the case of D. Pteronyssinus, causes a modification of the immune system with an increase in salivary-specific IgG4, IgA, and IgE, possibly correlating with symptom changes and QoL improvement [105].

Ren et al. evaluated the long-term efficacy of specific subcutaneous immunotherapy (SCIT) for mites with a follow-up ranging from 3 to 16 years. The group of children aged 5–14 years (*n* = 58) treated for three years with SCIT showed an improvement in symptoms and quality of life that persisted over time, even after discontinuation of therapy [106].

The use of sublingual immunotherapy has shown an improvement in symptoms and a better quality of life even outside pollen season, with less variability compared to the placebo group, and results maintained in the following pollen season [107]. A study on specific sublingual immunotherapy for grasses conducted in Italy demonstrated a 26.6% reduction in symptoms in the first year and 48.4% in the second year, with a significant improvement in the subject’s quality of life. A reduced response to conjunctival provocation was also reported, probably due to an increase in specific IgG4 and IgG1 [108].

In a trial on the efficacy of sublingual therapy for grass allergy conducted on 103 children under 15 years old, the therapy showed not only a reduction in symptoms during the pollen season but also a general improvement in quality of life [109].

An Italian study highlighted how daily therapy with SQ HDM SLIT tablet (ALK-Abellò A/S, Hørsholm, Denmark) improved QoL after just six months. A marked improvement in nasal symptoms, watery rhinorrhea, and sneezing was noted, along with immunological activation with an increase in sIgE for *D. pteronyssinus* and *D. farinae* and an increase in Der p1 and Der p 23, indicating immune activation [110]. Therapy with 12 SQ-HDM showed a 22% decrease in TCRS (Total Combined Rhinitis Score) and an improvement in daily symptoms, medication use, and quality of life. Only mild adverse effects were noted, with therapy discontinuation below 3% [111]. Symptom improvements are maintained even two to three years after the end of therapy [107,112,113,114,115]. These results have also been confirmed for sublingual therapy [116], as shown by Nolte et al., who reported a 17% decrease in TCRS one year after the start of therapy [117]. Considering SCIT (pre-seasonal or perennial) usage protocols, it was found that both show benefits on QoL, rhinoconjunctival symptoms, asthma control, medication use, and immune response (IgG4), with comparable safety [118].

Recommendation (Table 7). Due to the design of the research question, no quantitative analysis was performed and a consensus-based recommendation rather than an evidence-based one was framed and is shown in Table 7. In the presence of mild allergic rhinoconjunctivitis that does not respond to first-line therapies or moderate/severe allergic rhinoconjunctivitis, consultation with a pediatric allergy specialist allows for the identification of the causative agent, the initiation of specific therapies such as immunotherapy, and an improvement in quality of life.

##### PICO 2

In children and adolescents with allergic rhinoconjunctivitis, is performing a skin prick test for inhalant allergens preferred as first-line examination compared to measuring specific IgE to identify the causative agent of the symptoms?

Evidence (Narrative report). Allergic oculorhinitis is an IgE-mediated disease. Sensitization can be identified through a skin prick test (SPT), which assesses whether the suspected allergen elicits a local allergic response (also known as a “wheal”), or through a blood test to measure the presence of specific IgE (sIgE) directed against the allergen in question. Skin prick test (SPT) is usually performed in a specialist setting, as it requires specific preparation and proper handling of allergenic extracts [119]. However, the interpretation of the test can be influenced by factors such as age, body mass index, timing, instrument, and test methodology, making it less standardizable [120,121]. In most clinical studies, a positive sensitization is defined as a wheal 3–5 mm larger than the negative control. However, some practices consider any wheal equal to or larger than the positive control (histamine) as positive. The SPT remains the main in vivo test recommended by international guidelines, considered the most sensitive and specific for identifying allergic sensitization.

The measurement of specific IgE through a serological test is an in vitro test and is performed with a single blood draw, accompanied by the specific request of the allergen to be investigated. It is preferable to focus the investigation only on allergens suspected of having clinical relevance, avoiding overly broad tests (such as multiplex tests based on microarray techniques) that could highlight sensitizations without practical significance [122]. Unlike SPT, the test allows assessment of sensitization even in patients under antihistamine therapy [123].

A result is considered positive when the specific IgE value is equal to or greater than 0.35 IU/mL [119]. There is still no unanimous consensus within the scientific community on which method should be preferred as the first choice in diagnosing the allergen responsible for allergic rhinoconjunctivitis. Scientific evidence indicates moderate concordance, with agreement in approximately 50% of cases, between SPT and the measurement of specific IgE in serum (sIgE) in detecting sensitization to aeroallergens [124,125]. A factor to consider in evaluating both diagnostic methodologies is the possible cross-reactivity between allergens belonging to homologous groups [126]. Northern grass allergens such as timothy, vernal grass, ryegrass, meadow fescue, and Kentucky bluegrass are highly cross-reactive. Other relevant groups include cedar/cypress/juniper, ragweed/mugwort, and birch/oak/alder/hazel [126,127]. Regarding immunotherapy, the use of a single extract from a homologous group is sufficient to induce tolerance to other allergens within the same group [128].

A 2020 study conducted by Masrur et al. showed that 61.3% of patients (aged 10 to 60 years) diagnosed with allergic conjunctivitis had a negative skin prick test. Additionally, no correlation was found between the positivity of the skin test and the severity of symptoms [129]. However, other studies conducted exclusively on pediatric populations have shown the opposite: in these cases, a positive prick test for inhalant allergens such as birch and grass pollens, dust mites, or animal dander was associated with the onset of symptoms [130,131,132,133]. An element that aids in diagnosis in case of a negative SPT is the intradermal skin test (IDST). A study involving over 4200 patients with allergic rhinitis and/or bronchial asthma found a 44% positivity rate for IDST in patients who had a negative SPT despite presenting evident allergy symptoms [134].

Studies have estimated the sensitivity of the prick test to be around 85% and its specificity at 77% [135,136]. In a more recent study, a diagnostic panel for children aged 2 to 18 years old was developed in Turkey, where sensitization was identified in 95% of cases through the skin prick test alone [137].

The two tests find maximum concordance using a positivity threshold of ≥3 mm compared to the negative control for SPT (with a sensitivity of 74.1% and specificity of 76.0%) and values of 0.7 IU/mL or 3.5 IU/mL for sIgE (sensitivity of 84.5% and specificity of 57.2%) [124]. Numerous studies have investigated the role of serum and ocular IgE in improving diagnostic accuracy. Fernandes Polio et al., for example, have contributed to the creation of databases of relevant molecules in the field of allergology [138]. Currently, an ImmunoCAP test is being evaluated, aimed at diagnosing allergies based solely on the presence of specific IgE [130,139,140,141]. An Italian study analyzed the presence of sIgE for grass allergens (Phl p 1, 2, 4, 5b, 6, 7, 11, 12) and found that sensitization to Phl p 1, Phl p 7, and Phl p 12 offers clear diagnostic value, identifying primary sensitization and a higher risk of developing asthma and OSAS [142]. It was also found that the broader the sIgE response to grasses, the less intense the sensitization to dust mites. This suggests new perspectives on the immunological trajectory of allergies in pediatric age [143].

Some studies have highlighted greater specificity of the skin test for asthma and allergic rhinitis, with similar sensitivity [89,124]. Interestingly, an observational study conducted in Poland by Namyslowski et al. on over 4000 subjects found that some patients with negative prick tests still had specific IgE and symptoms compatible with allergic rhinitis [144]. However, positive results of SPT and specific serum IgE must always be interpreted in the context of the patient’s clinical history. A study conducted on the European population found that up to 40% of sensitizations to allergens were not clinically relevant, depending on the country and allergen considered [145].

A particular case is that of local allergic rhinitis (LAR), which is widely underdiagnosed in the pediatric population [146]. The pathology is a phenotype of rhinitis characterized by a type 2 inflammatory response, with local production of sIgE in the absence of positivity to SPT and serum sIgE [147]. In these cases, the nasal provocation test (performable from the age of 5) is useful: the measurement of local IgE in nasal lavage fluid is a useful tool to differentiate LAR from non-allergic rhinitis (NAR) and predict the response to classical allergic rhinitis therapy [148,149]. To support the diagnosis of LAR, the basophil activation test can be used, which has high sensitivity but is currently available only as a research tool [150]. New biomarkers such as circulating microRNAs and metabolites could contribute in the future to improving the diagnosis of allergic rhinitis [150,151,152].

Recommendation (Table 8). Due to the design of the research question, no quantitative analysis was performed and a consensus-based recommendation rather than an evidence-based one was framed and is reported in Table 8. The most effective diagnostic approach is the integration of the skin prick test with the measurement of serum IgE. Identifying the responsible allergen allows for the initiation of targeted immunotherapy, improving quality of life.

##### PICO 3

In children and adolescents with allergic rhinoconjunctivitis, is molecular diagnostics (component resolved diagnosis, CRD) more appropriate than the measurement of specific IgE to distinguish primary sensitizations from cross-reactivities and inform the selection of appropriate immunotherapy?

Evidence (Narrative report). Allergic Molecular Diagnostics, known as CRD (Component Resolved Diagnosis), represents a significant advancement in the evaluation of primary sensitizations and the selection of targeted immunotherapy. One of the most interesting aspects of CRD is its ability to distinguish between true primary sensitizations and cross-reactivities, particularly between inhalant and food allergens, which is crucial to avoid misdiagnoses in atopic individuals [130,153]. CRD allows the identification of specific “allergic endotypes” involved in the so-called pollen-food syndrome, including LTP (lipid transfer proteins), profilin, and PR010 proteins. These molecules are the basis of cross-reactions between pollens and certain foods [153].

CRD is also essential for the personalization of immunotherapy [90,154,155,156,157,158]. Several studies have reported that CRD is fundamental in increasing diagnostic accuracy (an increase of 50% compared to first-level tests) and in improving the appropriateness of immunotherapy prescription, particularly in polysensitized individuals [155,159,160]. Thanks to its precision, it allows identification of previously undiagnosed allergic patients; prescription of therapies targeted exclusively at the molecular component actually responsible for symptoms; modification of ongoing but ineffective immunotherapies; and improvement of patients’ quality of life by reducing both the use of symptomatic drugs and the intensity of daily symptoms.

Some relevant allergenic components, such as Ara h2, Ara h6 (peanuts), Phl p5 (grasses), Hev b5 (latex), and Amb a1 (ragweed), have proven useful for more accurately characterizing patients’ allergic profiles, particularly in relation to fruits and plants [161]. In 2023, Garriga Baraut et al. [158] used CRD to analyze the frequency of allergenic molecules such as Cup a1 and Cyn d1 in the Catalan population, thus expanding the knowledge of the main allergens present in the region. Another group focused on allergens related to dog dander, correlating the increase in specific IgE levels with the onset of clinical symptoms. The study by Knyziak et al. [162] demonstrated that some molecules are more implicated in the progression of the so-called ‘allergic march,’ which is the natural evolution of allergic diseases from the upper to the lower respiratory tract.

Furthermore, molecular diagnosis allows more accurate identification of patients most suitable for targeted immunotherapy, thus increasing the effectiveness of treatment. The creation of a molecular database would enable specialists to intervene early to mitigate the allergic march and prevent more severe symptoms in susceptible patients [154].

In a study on the use of molecular diagnosis (CRD) to analyze allergic sensitization in children and predict the development of asthma in adolescence, specific allergenic components associated with asthma, rhinitis, and eczema were identified. By including these allergenic components with a family history of asthma and early wheezing, it was possible to develop a predictive model of asthma at 13 years old with an accuracy of 82%, aimed at initiating preventive therapy early [163].

An observational study in 2018 reported that among 70 children with seasonal allergic rhinitis, after using CRD, immunotherapy was modified in 54.3% of cases. CRD also increased therapy indications from 18% to 51% of cases and suspension in 9.3% of cases, as the detected sensitization was not clinically relevant [155]. However, this technology still presents some limitations, including complex interpretation of results, high costs, and limited availability [163].

Recommendation. Due to the design of the research question, no quantitative analysis was performed and a consensus-based recommendation rather than an evidence-based one was framed (Table 9). CRD is currently used as a resource in allergy diagnosis. It provides information that can support more effective and individualized therapeutic decisions.

### 3.3. Vernal Keratoconjunctivitis

#### 3.3.1. Summary of the Literature Research

The detailed description of search strategies is reported in Table 10.

**Figure 4:**
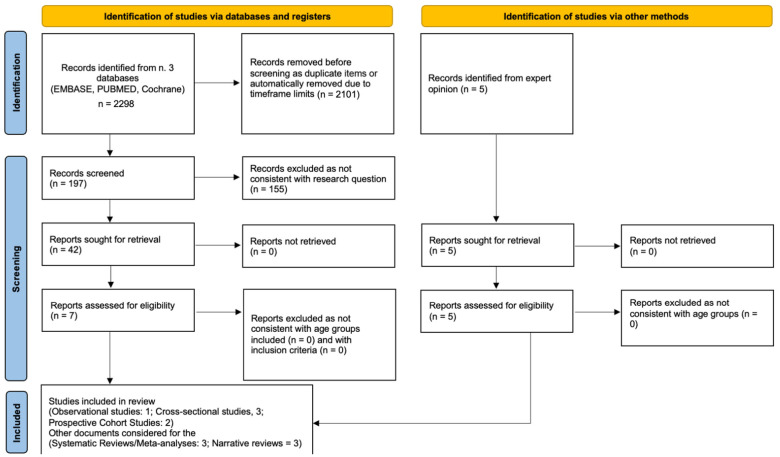
Flowchart of studies on vernal keratoconjunctivitis. See Appendix A Table A1 for the detailed search strategy [6].

As shown in Figure 4, the search on three databases (Embase, Medline, Cochrane) identified a total of 2298 articles. After the removal of duplicates and articles published before the timeframe limits (*n* = 2101, 91.4%), 197 studies were screened by title and abstract (8.6%). Most of them (i.e., *n* = 155, 6.7%) were removed as they were not consistent with research aims, leading to the retrieval of 42 articles that were reviewed by their full text (1.8%): 7 of them were included in the final analysis (0.01%). Based on expert opinion, 5 additional articles were added, for a total of 12 articles included.

Overall, the search strategy identified 1 observational study, 3 cross-sectional studies, 2 prospective cohort studies, 3 systematic reviews and 3 narrative reviews). Their detailed description is included in Appendix A Table A2, while their qualitative appraisal is provided by Appendix A Table A7.

#### 3.3.2. Clinical Questions

##### PICO 1

In children and adolescents with suspected VKC, is a pediatric ophthalmological/allergological consultation recommended over autonomous management by the primary care pediatrician to improve diagnosis and disease management?

Evidence (Narrative report). Vernal keratoconjunctivitis (VKC) is a chronic and bilateral eye disease that predominantly affects atopic children and adolescents, particularly males living in warm climates. Numerous scientific studies demonstrate the importance of sunlight exposure. Conversely, the role of allergic sensitization is more marginal and still debated [164,165]. Vernal keratoconjunctivitis can cause severe ocular complications if not adequately treated. Given its potential severity, accurate diagnosis and effective management are crucial [165]. Vernal keratoconjunctivitis is often associated with other allergic conditions such as asthma, atopic dermatitis, and allergic rhinitis. A systematic review with meta-analysis shows that approximately 57.7% of patients with VKC have allergic sensitization, particularly to inhalant allergens such as dust mites and pollens. Diagnosis typically requires a comprehensive evaluation, including clinical examination and allergy tests [166].

Clinically, VKC is characterized by intense ocular itching, photophobia, tearing, foreign body sensation, abundant mucous discharge, especially in the morning, ocular pain (especially if the cornea is involved), and blurred vision. Ophthalmological signs include the presence of: (a) giant papillae: hyperemic and hypertrophic papillae on the upper tarsal conjunctiva, with a “cobblestone” appearance; (b) Maxwell–Lyons sign: an accumulation of fibrin on the surface of the giant papillae of the upper palpebral conjunctiva, which occurs when mucous secretions are exposed to heat; (c) Horner–Trantas nodules: white gelatinous nodules at the corneal limbus, representing aggregates of epithelial cells and eosinophils [4,167,168].

A misdiagnosis can lead to inappropriate treatments, potentially worsening the condition. Effective management of VKC often requires a multidisciplinary approach. While primary care pediatricians are important for the initial evaluation and for raising suspicion of VKC through a thorough history and meticulous physical examination, ophthalmologists and allergists are essential for making a definitive diagnosis and managing moderate to severe VKC cases [164].

In addition to establishing a definitive diagnosis, specialist ophthalmological and allergological consultations are recommended for: performing allergy tests useful for identifying specific allergens and setting up targeted therapies [166,169]; immunotherapy in patients with significant allergic sensitization [170]; advanced pharmacological management beyond mast cell stabilizers and topical antihistamines, including the possible administration of corticosteroids and other immunomodulatory agents such as cyclosporine and tacrolimus or biological drugs like omalizumab [171,172].

Early diagnosis and regular ophthalmological monitoring of VKC are essential to assess severity and detect complications like keratitis, corneal ulcers, or keratoconus [167].

Recommendation. Due to the design of the research question, no quantitative analysis was performed and a consensus-based recommendation rather than an evidence-based one was framed (Table 11). Moreover, substantially few pediatric studies addressing diagnostic appropriateness and referral pathways were identified, particularly when compared with previous sections. In summary, while primary care pediatricians and general practitioners initiate the diagnostic process of suspected VKC, pediatric ophthalmological and allergological consultations are advised to confirm the diagnosis, determine specific allergens, and initiate appropriate treatment strategies.

##### PICO 2

In children and adolescents with suspected VKC, may allergy tests such as prick testing and specific IgE measurement provide additional information beyond clinical evaluation alone for identifying potential allergic triggers and guiding therapy?

Evidence (Narrative report). In VKC, the clinical history and ocular examination are fundamental components of the diagnostic work-up, but identifying underlying allergic triggers can also be useful to distinguish this form of conjunctivitis from others and to set up personalized therapy [165,166,173]. Although the role of allergens is debated, allergy tests, particularly prick tests and the measurement of specific IgE, play an important role in understanding the atopic profile of patients. About 30–50% of patients, however, do not show sensitization to any allergen. A study by Wang et al. [169] that analyzed serum levels of specific IgE in children with allergic conjunctivitis found a high prevalence of sensitization to common aeroallergens such as pollen and dust mites, with a significant presence of allergic comorbidities such as asthma and rhinitis. These results underline the importance of comprehensive allergy tests to identify an atopic condition and environmental triggering factors, which are not detectable through symptoms alone. Furthermore, evidence of elevated IgE levels may support, in selected cases, the initiation of therapy with biologics such as omalizumab [167]. Rasmussen et al., in a systematic review with meta-analysis on patients with VKC, reported that over 57% of subjects showed sensitization to at least one allergen, with the highest detection rates obtained through the determination of tear IgE. This supports the usefulness of allergy tests in revealing occult sensitizations that might escape clinical observation [166,171]. VKC significantly reduces health-related quality of life in children, highlighting the need for a more precise and targeted therapeutic approach [171]. Early identification of allergic triggers can allow for the adoption of personalized allergen avoidance strategies and, where indicated, the initiation of specific immunotherapy, thus improving long-term outcomes and quality of life [172,174]. A systematic review by Berger et al. further emphasized the importance of an integrated diagnostic approach, noting that the identification of allergic sensitization allows clinicians to refine therapeutic decisions, including the choice of antihistamines, mast cell stabilizers, corticosteroids, and, in selected cases, immunotherapy [167,172].

Recommendation. Due to the design of the research question, no quantitative analysis was performed and a consensus-based recommendation rather than an evidence-based one was framed (Table 12). Allergy tests, including skin prick tests and the measurement of specific IgE, provide valuable information for identifying the allergens responsible for exacerbating VKC symptoms and support a personalized treatment plan based on the etiology. Therefore, in children and adolescents with suspected VKC, it is recommended to perform allergy tests rather than relying solely on clinical evaluation.

## 4. Discussion

The present multidisciplinary initiative sought to develop evidence-informed clinical recommendations addressing the appropriateness of specialist consultations and diagnostic test prescriptions in pediatric allergology. Across the evaluated conditions—asthma, allergic rhinoconjunctivitis, and VKC—the synthesis of available evidence consistently supports the clinical value of structured diagnostic pathways integrating specialist expertise with targeted use of objective diagnostic tools. These findings are particularly relevant within contemporary healthcare systems, where variability in diagnostic practices, inappropriate testing, and delayed referrals continue to represent significant challenges affecting both patient outcomes and resource utilization.

A recurring theme emerging from the literature is that symptom-based diagnostic approaches alone often prove insufficient in pediatric allergic diseases. Clinical presentations in children are frequently nonspecific, overlapping across conditions, and influenced by developmental, environmental, and behavioral factors. In this context, the incorporation of objective measurements and specialist evaluation plays a pivotal role in improving diagnostic precision and guiding rational therapeutic decisions.

In asthma, for example, the integration of spirometry, bronchodilator reversibility testing, inflammatory biomarkers such as FeNO, and allergy testing contributes to a more accurate characterization of disease phenotypes. Specialist assessment further enhances disease management by identifying modifiable contributors to poor control, including incorrect inhalation technique, suboptimal adherence, persistent allergen exposure, and potential diagnostic misclassification. These dimensions, although clinically critical, may be difficult to systematically address within routine primary care settings due to structural and organizational constraints. The present findings reinforce guideline recommendations [2], which recognize that optimal asthma management extends beyond pharmacological treatment and requires a multidimensional assessment encompassing diagnostic confirmation, phenotypic characterization, inhaler technique, adherence, and environmental exposures. The greater diagnostic appropriateness observed following specialist evaluation may reduce the risk of diagnostic misclassification and inappropriate management decisions.

Similar considerations apply to allergic rhinoconjunctivitis, for which accurate etiological diagnosis is essential to optimize treatment, particularly when allergen-specific immunotherapy is considered. Specialist-led evaluation supports a more reliable interpretation of sensitization patterns, helps identify complex entities such as local allergic rhinitis, and distinguishes clinically relevant sensitizations from incidental findings. This is increasingly important as diagnostic technologies become more advanced, since over-testing or misinterpretation may lead to inappropriate management. These considerations are consistent with EAACI recommendations [3], which state that allergen-specific strategies, including immunotherapy, require integration of clinical history with evidence of clinically relevant sensitization. The same guideline also emphasizes the need to interpret sensitization profiles within the appropriate clinical context to avoid unnecessary testing and prevent inappropriate therapeutic decisions.

In VKC, the role of multidisciplinary specialist involvement is crucial given the chronicity, potential severity, and risk of complications associated with this condition. Early recognition, accurate diagnostic classification, and personalized therapeutic strategies may substantially influence disease trajectories and quality of life.

Our results further reinforce this patient-centered approach and the concept that diagnostic appropriateness extends beyond the mere selection of tests and represents a complex clinical process integrating evidence, clinical expertise, patient characteristics, and benefit–risk considerations.

The methodological framework adopted in this initiative represents a relevant strength, as it combines systematic evidence synthesis with structured expert consensus, allowing recommendations to be formulated even in areas characterized by limited or heterogeneous evidence. The application of GRADE methodology ensured transparency in evaluating the certainty of evidence and facilitated balanced clinical judgments. Moreover, the multidisciplinary composition of the expert panel enhanced the clinical relevance and pragmatic applicability of the recommendations by incorporating diverse professional perspectives reflective of real-world pediatric care.

Importantly, recommendation strength was determined through a structured Evidence-to-Decision process that considered not only certainty of evidence, but also the balance of benefits and harms, patient values and preferences, feasibility, equity, acceptability, and resource implications. Consequently, in selected cases, recommendation strength did not directly mirror the certainty of evidence alone.

Nevertheless, several limitations warrant consideration. The certainty of evidence underlying many research questions ranged from moderate to low, primarily due to heterogeneity in study designs, variability in outcome definitions, and the relative scarcity of pediatric-specific randomized controlled trials. The reliance on observational data, although appropriate in many contexts, inherently limits causal inference.

Furthermore, substantial heterogeneity in populations, interventions, outcome definitions, and study methodologies frequently precluded quantitative synthesis. For this reason, narrative evidence synthesis was preferred in several PICO questions to avoid potentially misleading pooled estimates.

Additionally, the absence of quantitative meta-analytic synthesis for several clinical questions may reduce the precision of estimated effects, though this limitation largely reflects the nature of the available literature rather than methodological shortcomings. As with most consensus-based initiatives, expert judgment inevitably contributed to recommendation development, introducing a degree of subjectivity that should be acknowledged. Finally, the present study did not evaluate real-world implementation outcomes, and future research should assess adherence to these recommendations, their impact on diagnostic practices, and their implications for healthcare utilization and patient outcomes.

Future research should prioritize high-quality pediatric randomized controlled trials, prospective implementation studies, and comparative evaluations of diagnostic pathways to strengthen the evidence base supporting appropriateness recommendations in pediatric allergology.

Despite these limitations, the convergence of evidence, clinical plausibility, and expert consensus provides a coherent framework supporting more rational, standardized, and patient-centered diagnostic strategies in pediatric allergology. Improved diagnostic appropriateness has the potential to enhance clinical outcomes, reduce unnecessary investigations, optimize therapeutic decisions, and contribute to the sustainability of healthcare systems.

## 5. Conclusions

This multidisciplinary initiative provides an evidence-informed framework for improving the appropriateness of specialist consultations and diagnostic test prescriptions in pediatric allergology. Across asthma, allergic rhinoconjunctivitis, and VKC, the integration of structured diagnostic pathways, objective testing, and specialist expertise consistently emerges as a clinically valuable strategy. These approaches enhance diagnostic precision, support more rational therapeutic decisions, and contribute to optimized disease management in pediatric patients, a population in which symptom-based evaluation alone often proves insufficient.

Although the certainty of evidence underlying several recommendations ranged from moderate to low, the convergence of available data, clinical plausibility, and expert consensus supports the adoption of targeted, clinically driven diagnostic strategies. Diagnostic appropriateness should be regarded not merely as a matter of test selection but as a comprehensive clinical process integrating patient characteristics, disease heterogeneity, benefit–risk considerations, and healthcare resource implications. Importantly, recommendation strength was determined through a structured Evidence-to-Decision process that incorporated certainty of evidence together with feasibility, acceptability, patient values and preferences, and resource implications.

The implementation of these recommendations has the potential to improve the quality of care, reduce variability in clinical practice, minimize unnecessary investigations, and promote more efficient utilization of healthcare resources. In addition to enhancing patient outcomes, more rational diagnostic pathways may contribute to healthcare system sustainability by reducing inappropriate testing and delayed diagnoses. Emerging models of care, including telemedicine and digital health tools, may further facilitate access to specialist expertise and support standardized diagnostic decision-making. The heterogeneity of the available literature highlights the need for greater methodological standardization in future studies evaluating diagnostic strategies in pediatric allergology.

Future research should prioritize high-quality pediatric studies evaluating diagnostic strategies, real-world implementation, and long-term clinical outcomes. Strengthening the evidence base will be essential for refining diagnostic algorithms and advancing precision medicine approaches in pediatric allergic diseases.

## Figures and Tables

**Table 1:** Summary of inquired PICOs on allergic asthma.

	PICO 1	PICO 2	PICO 3	PICO 4
Population	children and adolescents with uncontrolled allergic asthma	children and adolescents with suspected allergic asthma	children and adolescents with allergic asthma	in children and adolescents with suspected allergic asthma
(age)	<18 years	<18 years	<18 years	<18 years
Intervention	pediatric allergy or pediatric pulmonology visit	allergy tests (e.g., skin prick test and measurement of specific IgE)	spirometry with bronchodilator reversibility test	spirometry combined with the measurement of exhaled nitric oxide (bronchial FeNO)
Comparator	only management by the primary care pediatrician	clinical assessment alone	clinical evaluation alone	spirometry alone
Outcome	improvement of therapy adherence and disease control	identification of triggers and improving disease control	improvement of diagnostic accuracy and asthma control	improvement of diagnostic accuracy and therapeutic decisions

**Table 2:** Summary of quality of evidence on the PICO 1 (i.e., “In children and adolescents with uncontrolled allergic asthma, is the allergy or pulmonology visit recommended compared to the only management by the primary care pediatrician to improve therapy adherence and disease control?”). Note: GRADE = Grading of Recommendations Assessment, Development and Evaluation.

Quality of Evidence (GRADE)	Strength	Balance of Benefits/Risks	Values and Preferences	Impact on Resources/Implementation
⨁⨁◯◯	Conditional recommendation favors specialist evaluation (pediatric pulmonology/allergology).	Benefits Improved diagnostic accuracy Optimization of maintenance therapy Identification of modifiable causes of poor control Improved adherence and asthma control Potential reduction in exacerbations and healthcare utilization Risks Increased healthcare resource utilization Potential delays in access where specialist services are limited	Families and caregivers generally value accurate diagnosis, individualized management, and access to specialist expertise. Acceptability is expected to be high, particularly in patients with persistent poor asthma control despite primary care management.	Requires access to specialist services and may increase short-term healthcare costs; however, these costs may be partially offset by improved disease control and a reduction in exacerbations, emergency visits, and hospitalizations.

Note: ⨁⨁⨁⨁ = high certainty of evidence; ⨁⨁⨁◯ = moderate certainty of evidence; ⨁⨁◯◯ = low certainty of evidence; ⨁◯◯◯ = very low certainty of evidence; ◯◯◯◯ = absence of evidence answering the PICO.

**Table 3:** Summary of quality of evidence on the PICO 2 (i.e., “In children and adolescents with suspected allergic asthma, do allergy tests (such as the skin prick test and measurement of specific IgE) offer greater effectiveness than clinical assessment alone in identifying triggers and improving disease control?”). Note: GRADE = Grading of Recommendations Assessment, Development and Evaluation.

Quality of Evidence (GRADE)	Strength	Balance of Benefits/Risks	Values and Preferences	Impact on Resources/Implementation
⨁⨁◯◯	Strong recommendation in favor of allergy testing (skin prick testing and allergen-specific IgE assessment).	Benefits Supports diagnostic assessment Identifies clinically relevant sensitizations Facilitates phenotyping and risk stratification Guides allergen avoidance measures Supports selection of targeted therapies (e.g., immunotherapy, biologics) Risks Additional costs and resource utilization Time required for testing Limited diagnostic value when interpreted without clinical context	Families and caregivers generally value diagnostic clarification and identification of potential triggers. Allergy testing is widely accepted in pediatric practice and may facilitate shared decision-making regarding environmental control measures and targeted therapies.	Requires trained personnel and dedicated diagnostic resources. Although allergy testing increases short-term healthcare utilization, it is already incorporated into routine specialist assessment in many healthcare settings and is generally considered feasible to implement.

Note: ⨁⨁⨁⨁ = high certainty of evidence; ⨁⨁⨁◯ = moderate certainty of evidence; ⨁⨁◯◯ = low certainty of evidence; ⨁◯◯◯ = very low certainty of evidence; ◯◯◯◯ = absence of evidence answering the PICO.

**Table 4:** Summary of quality of evidence on the PICO 3 (i.e., “For children and adolescents with allergic asthma, does spirometry with bronchodilator reversibility improves diagnostic accuracy and asthma control compared to clinical evaluation alone”). Note: GRADE = Grading of Recommendations Assessment, Development and Evaluation.

Quality of Evidence (GRADE)	Strength	Balance of Benefits/Risks	Values and Preferences	Impact on Resources/Implementation
⨁⨁⨁◯	Strong recommendation in favor of spirometry with bronchodilator reversibility testing	Benefits Improved diagnostic accuracy Improved assessment of disease severity Support for therapeutic decision-making Risks Additional costs and time demands, Patient cooperation required	High acceptance among patients, caregivers, and pediatricians, as spirometry is considered a standard diagnostic procedure.	Requires spirometry equipment and trained personnel; however, implementation is feasible as spirometry already represents standard practice in many specialist settings.

Note: ⨁⨁⨁⨁ = high certainty of evidence; ⨁⨁⨁◯ = moderate certainty of evidence; ⨁⨁◯◯ = low certainty of evidence; ⨁◯◯◯ = very low certainty of evidence; ◯◯◯◯ = absence of evidence answering the PICO.

**Table 5:** Summary of quality of evidence on the PICO 4 (i.e., “In children and adolescents with suspected allergic asthma, spirometry combined with the measurement of exhaled nitric oxide (bronchial FeNO) is more useful than spirometry alone to improve diagnostic accuracy and guide therapeutic decisions”). Note: GRADE = Grading of Recommendations Assessment, Development and Evaluation.

Quality of Evidence (GRADE)	Strength	Balance of Benefits/Risks	Values and Preferences	Impact on Resources/Implementation
⨁⨁⨁◯	Strong recommendation in favor of integrating spirometry with FeNO measurement.	Benefits Improved diagnostic accuracy Improved assessment of eosinophilic airway inflammation Support for treatment optimization and monitoring Risks Additional costs; potential influence of underlying conditions on FeNO values (diagnostic imprecision)	Clinicians value biomarkers that support personalized treatment decisions. Acceptability among families is generally high, provided that the impact on daily activities remains limited, with a favorable balance between benefits and family burden.	Requires spirometry equipment and trained personnel; the overall impact on healthcare resources is moderate and may lead to long-term savings through improved disease management.

Note: ⨁⨁⨁⨁ = high certainty of evidence; ⨁⨁⨁◯ = moderate certainty of evidence; ⨁⨁◯◯ = low certainty of evidence; ⨁◯◯◯ = very low certainty of evidence; ◯◯◯◯ = absence of evidence answering the PICO.

**Table 6:** Summary of inquired PICOs on rhinoconjunctivitis.

	PICO 1	PICO 2	PICO 3
Population	children and adolescents with mild allergic rhinoconjunctivitis not responding to not respond to first-line therapy (topical nasal corticosteroids and/or oral antihistamines) OR moderate/severe allergic rhinoconjunctivitis	children and adolescents with allergic rhinoconjunctivitis	children and adolescents with allergic rhinoconjunctivitis
(age)	<18 years	<18 years	<18 years
Intervention	consultation with a pediatric allergy specialist	skin prick test for inhalant allergens	molecular diagnostics (component resolved diagnosis, CRD)
Comparator	routine follow-up with a general pediatrician	measurement of specific IgE	measurement of specific IgE
Outcome	identification of the causative agent, AND/OR initiate specific immunotherapy, AND/OR improve the quality of life	identification of the causative agent of the symptoms	distinguish primary sensitizations from cross-reactivities and inform the selection of appropriate immunotherapy

**Table 7:** Summary of quality of evidence on the PICO 1 (i.e., “In children and adolescents with mild allergic rhinoconjunctivitis that does not respond to first-line therapy (topical nasal corticosteroids and/or oral antihistamines) or moderate/severe allergic rhinoconjunctivitis, is a consultation with a pediatric allergy specialist recommended over routine follow-up with a general pediatrician to identify the causative agent, initiate specific immunotherapy, and improve the quality of life?”) Note: GRADE = Grading of Recommendations Assessment, Development and Evaluation.

Quality of Evidence (GRADE)	Strength	Balance of Benefits/Risks	Values and Preferences	Impact on Resources/Implementation
⨁⨁⨁◯	Strong recommendation in favor of integrating routine follow-up with consultation by a pediatric allergy specialist.	Greater diagnostic precision and optimization of diagnostic accuracy may be achieved; however, the potential impact on specialist service availability should be taken into account.	Families are generally supportive of specialist evaluation; however, coordination with the primary care pediatrician is advisable to optimize continuity of care and minimize the overall impact on healthcare delivery.	Implementation requires access to specialist allergy services and appropriate referral pathways. Resource utilization is expected to be sustainable provided that appropriate patient selection is ensured.

Note: ⨁⨁⨁⨁ = high certainty of evidence; ⨁⨁⨁◯ = moderate certainty of evidence; ⨁⨁◯◯ = low certainty of evidence; ⨁◯◯◯ = very low certainty of evidence; ◯◯◯◯ = absence of evidence answering the PICO.

**Table 8:** Summary of quality of evidence on the PICO 2 (i.e., “In children and adolescents with allergic rhinoconjunctivitis, is performing a skin prick test for inhalant allergens preferred as first0line examination compared to measuring specific IgE to identify the causative agent of the symptoms?”). Note: GRADE = Grading of Recommendations Assessment, Development and Evaluation.

Quality of Evidence (GRADE)	Strength	Balance of Benefits/Risks	Values and Preferences	Impact on Resources/Implementation
⨁⨁⨁◯	Strong recommendation in favor of integrating specialist evaluation with skin prick testing for inhalant allergens.	Benefits include greater diagnostic accuracy, lower cost, and rapid results; risks are generally limited to mild local skin discomfort.	Patients and caregivers are generally supportive of specialist evaluation, particularly in severe or persistent cases requiring access to specialist care.	Skin prick testing requires trained personnel and allergen extracts but is generally more cost-effective than routine measurement of serum-specific IgE.

Note: ⨁⨁⨁⨁ = high certainty of evidence; ⨁⨁⨁◯ = moderate certainty of evidence; ⨁⨁◯◯ = low certainty of evidence; ⨁◯◯◯ = very low certainty of evidence; ◯◯◯◯ = absence of evidence answering the PICO.

**Table 9:** Summary of quality of evidence on the PICO 3 (i.e., “In children and adolescents with allergic rhinoconjunctivitis, is molecular diagnostics (component resolved diagnosis, CRD) more appropriate than the measurement of specific IgE to distinguish primary sensitizations from cross-reactivities and inform the selection of appropriate immunotherapy?”). Note: GRADE = Grading of Recommendations Assessment, Development and Evaluation.

Quality of Evidence (GRADE)	Strength	Balance of Benefits/Risks	Values and Preferences	Impact on Resources/Implementation
⨁⨁⨁◯	Strong recommendation in favor of integrating molecular diagnostics (Component-Resolved Diagnosis, CRD) into specialist follow-up.	Benefits are likely to outweigh risks, including improved etiological diagnosis, more targeted therapy, and potential symptom reduction, compared with potential drawbacks such as delayed or duplicated consultations.	Clinicians are generally supportive of this approach; families tend to prefer diagnostic tests that are less invasive and provide rapid results.	Resource utilization may increase because of the costs and infrastructure required for molecular diagnostics; however, targeted implementation in selected patients may lead to long-term improvements in diagnostic efficiency and resource allocation.

Note: ⨁⨁⨁⨁ = high certainty of evidence; ⨁⨁⨁◯ = moderate certainty of evidence; ⨁⨁◯◯ = low certainty of evidence; ⨁◯◯◯ = very low certainty of evidence; ◯◯◯◯ = absence of evidence answering the PICO.

**Table 10:** Summary of inquired PICOs on vernal keratoconjunctivitis (VKC).

	PICO 1	PICO 2
Population	children and adolescents with suspected vernal keratoconjunctivitis (VKC),	children and adolescents with suspected vernal keratoconjunctivitis (VKC),
(age)	<18 years	<18 years
Intervention	pediatric ophthalmological/allergological consultation	allergy tests such as prick testing and specific IgE measurement
Comparator	autonomous management by the primary care pediatrician	clinical evaluation
Outcome	improve diagnosis and disease management	identification of triggers and improving disease control

**Table 11:** Summary of quality of evidence on the PICO 1 (i.e., “In children and adolescents with suspected vernal keratoconjunctivitis (VKC), is a specialist ophthalmological/allergological consultation recommended over autonomous management by the primary care pediatrician to improve diagnosis and disease management?”). Note: GRADE = Grading of Recommendations Assessment, Development and Evaluation.

Quality of Evidence (GRADE)	Strength	Balance of Benefits/Risks	Values and Preferences	Impact on Resources/Implementation
◯◯◯◯	Conditional recommendation in favor of specialist assessment (ophthalmological/allergological).	Probable benefit (early diagnosis, prevention of corneal complications) with minimal risks.	Families and pediatricians in favor of specialist consultation.	Requires access to specialist services and may increase healthcare resource utilization; however, early diagnosis may help prevent sight-threatening complications.

Note: ⨁⨁⨁⨁ = high certainty of evidence; ⨁⨁⨁◯ = moderate certainty of evidence; ⨁⨁◯◯ = low certainty of evidence; ⨁◯◯◯ = very low certainty of evidence; ◯◯◯◯ = absence of evidence answering the PICO.

**Table 12:** Summary of quality of evidence on the PICO 1 (i.e., “In children and adolescents with suspected vernal keratoconjunctivitis (VKC), may allergy tests such as prick testing and specific IgE measurement provide additional information beyond clinical evaluation alone for identifying potential allergic triggers and guiding therapy?”). Note: GRADE = Grading of Recommendations Assessment, Development and Evaluation.

Quality of Evidence (GRADE)	Strength	Balance of Benefits/Risks	Values and Preferences	Impact on Resources/Implementation
◯◯◯◯	Conditional recommendation in favor of allergy testing to support diagnosis and individualized therapy.	Potential benefit in identifying relevant triggers; minimal risks associated with skin prick testing and blood sampling.	Families are generally supportive; clinicians may differ regarding the usefulness of allergy testing in all forms of VKC.	Available in most allergy centers, with relatively low cost; however, testing may not be necessary in all patients.

Note: ⨁⨁⨁⨁ = high certainty of evidence; ⨁⨁⨁◯ = moderate certainty of evidence; ⨁⨁◯◯ = low certainty of evidence; ⨁◯◯◯ = very low certainty of evidence; ◯◯◯◯ = absence of evidence answering the PICO.

## Data Availability

The original contributions presented in this study are included in the article/Supplementary Material. Further inquiries can be directed to the corresponding author(s).

## Supplementary Materials

The following supporting information can be downloaded at: https://www.mdpi.com/article/10.3390/jcm15124848/s1. Supplementary Materials Table S1. PRISMA 2020 Checklist.

## Appendix A

**Table A1:** Summary of search strategies.

TOPIC	PUBMED	EMBASE	Cochrane
Allergic Asthma, PICO 1	(“Asthma”[MeSH] OR “Allergic asthma” OR “allergic asthma”[tiab] OR “asthma control”[tiab] OR “uncontrolled asthma”[tiab] OR “mild persistent asthma”[tiab]) AND (“Referral and Consultation”[MeSH] OR specialist[tiab] OR “asthma specialist”[tiab] OR allergolog+1[tiab] OR allergist[tiab] OR pneumolog+1[tiab] OR pulmonolog+1[tiab]) AND (“Pediatrics”[MeSH] OR pediatric[tiab] OR paediatric[tiab] OR child[tiab] OR adolescent[tiab]) AND (“Medication Adherence”[MeSH] OR adherence[tiab] OR “treatment adherence”[tiab] OR “therapy compliance”[tiab] OR compliance[tiab]) AND (“asthma control”[tiab] OR “controlled asthma”[tiab] OR “asthma control test”[tiab] OR “ACT score”[tiab])	(‘asthma’/exp OR ‘allergic asthma’/exp OR ‘allergic asthma’:ti,ab OR ‘asthma control’:ti,ab OR ‘uncontrolled asthma’:ti,ab OR ‘mild persistent asthma’:ti,ab) AND (‘referral’/exp OR ‘consultation’/exp OR specialist:ti,ab OR ‘asthma specialist’:ti,ab OR allergolog+1:ti,ab OR allergist+1:ti,ab OR pneumolog+1:ti,ab OR pulmonolog+1:ti,ab) AND (‘pediatrics’/exp OR pediatric+1:ti,ab OR paediatric+1:ti,ab OR child+1:ti,ab OR adolescent+1:ti,ab) AND (‘medication compliance’/exp OR adherence:ti,ab OR ‘treatment adherence’:ti,ab OR ‘therapy compliance’:ti,ab OR compliance:ti,ab) AND (‘asthma control’:ti,ab OR ‘controlled asthma’:ti,ab OR ‘asthma control test’:ti,ab OR ‘act score’:ti,ab)	((MeSH descriptor: [Asthma] explode all trees) OR “Allergic asthma”:ti,ab,kw OR “allergic asthma”:ti,ab,kw OR “asthma control”:ti,ab,kw OR “uncontrolled asthma”:ti,ab,kw OR “mild persistent asthma”:ti,ab,kw) AND ((MeSH descriptor: [Referral and Consultation] explode all trees) OR specialist:ti,ab,kw OR “asthma specialist”:ti,ab,kw OR allergolog+1:ti,ab,kw OR allergist:ti,ab,kw OR pneumolog+1:ti,ab,kw OR pulmonolog+1:ti,ab,kw) AND ((MeSH descriptor: [Pediatrics] explode all trees) OR pediatric:ti,ab,kw OR paediatric:ti,ab,kw OR child:ti,ab,kw OR adolescent:ti,ab,kw) AND ((MeSH descriptor: [Medication Adherence] explode all trees) OR adherence:ti,ab,kw OR “treatment adherence”:ti,ab,kw OR “therapy compliance”:ti,ab,kw OR compliance:ti,ab,kw)
Allergic Asthma, PICO 2	(“Asthma”[MeSH] OR “Allergic asthma” OR “asthma allergic”[tiab] OR “suspected asthma”) AND (“Skin Tests”[MeSH] OR “Prick test”[tiab] OR “Skin prick test”[tiab] OR “Allergy testing”[tiab] OR “Allergen skin test”[tiab] OR “IgE” OR “specific IgE”[tiab] OR “sIgE”[tiab] OR “RAST”[tiab] OR “ImmunoCAP”[tiab]) AND (“Diagnosis”[MeSH] OR diagnos+1[tiab] OR “trigger”[tiab] OR “triggers”[tiab] OR “allergen identification”[tiab] OR “allergen exposure”[tiab]) AND (“Pediatrics”[MeSH] OR child[tiab] OR adolescent[tiab] OR pediatric[tiab] OR paediatric[tiab])	(‘asthma’/exp OR ‘allergic asthma’/exp OR ‘asthma allergic’:ti,ab OR ‘suspected asthma’:ti,ab) AND (‘skin test’/exp OR ‘skin prick test’/exp OR ‘prick test’:ti,ab OR ‘skin prick test’:ti,ab OR ‘allergy testing’:ti,ab OR ‘allergen skin test’:ti,ab OR ‘immunoglobulin e’/exp OR ‘specific immunoglobulin e’:ti,ab OR sige:ti,ab OR rast:ti,ab OR immunocap:ti,ab) AND (‘diagnosis’/exp OR diagnos+1:ti,ab OR trigger+1:ti,ab OR ‘allergen identification’:ti,ab OR ‘allergen exposure’:ti,ab) AND (‘pediatrics’/exp OR child+1:ti,ab OR adolescent+1:ti,ab OR pediatric+1:ti,ab OR paediatric+1:ti,ab)	((MeSH descriptor: [Asthma] explode all trees) OR “Allergic asthma”:ti,ab,kw OR “asthma allergic”:ti,ab,kw OR “suspected asthma”:ti,ab,kw) AND ((MeSH descriptor: [Skin Tests] explode all trees) OR “Prick test”:ti,ab,kw OR “Skin prick test”:ti,ab,kw OR “Allergy testing”:ti,ab,kw OR “Allergen skin test”:ti,ab,kw OR IgE:ti,ab,kw OR “specific IgE”:ti,ab,kw OR sIgE:ti,ab,kw OR RAST:ti,ab,kw OR ImmunoCAP:ti,ab,kw) AND ((MeSH descriptor: [Diagnosis] explode all trees) OR diagnos+1:ti,ab,kw OR trigger:ti,ab,kw OR triggers:ti,ab,kw OR “allergen identification”:ti,ab,kw OR “allergen exposure”:ti,ab,kw) AND ((MeSH descriptor: [Pediatrics] explode all trees) OR child:ti,ab,kw OR adolescent:ti,ab,kw OR pediatric:ti,ab,kw OR paediatric:ti,ab,kw)
Allergic Asthma, PICO 3	(“Asthma”[MeSH] OR “ Allergic asthma” OR “asthma allergic”[tiab] OR “suspected asthma”[tiab]) AND (“Spirometry”[MeSH] OR spirometr+1[tiab] OR “Pulmonary Function Test+1” OR “bronchodilator test”[tiab] OR “bronchodilator response”[tiab] OR “post0bronchodilator”[tiab] OR “BDR test”[tiab]) AND (diagnosis[MeSH Terms] OR diagnos+1[tiab] OR “diagnostic accuracy”[tiab] OR “disease management”[MeSH] OR “clinical management”[tiab] OR “healthcare utilization”[tiab] OR “emergency department”[tiab] OR “emergency service, hospital”[MeSH] OR “primary care visits”[tiab]) AND (“Pediatrics”[MeSH] OR child[tiab] OR adolescent[tiab] OR pediatric[tiab] OR paediatric[tiab])	(‘asthma’/exp OR ‘allergic asthma’/exp OR ‘asthma allergic’:ti,ab OR ‘suspected asthma’:ti,ab) AND (‘spirometry’/exp OR spirometr+1:ti,ab OR ‘pulmonary function test’/exp OR ‘pulmonary function test+1’:ti,ab OR ‘bronchodilator test’:ti,ab OR ‘bronchodilator response’:ti,ab OR ‘post0bronchodilator’:ti,ab OR ‘bdr test’:ti,ab) AND (‘diagnosis’/exp OR diagnos+1:ti,ab OR ‘diagnostic accuracy’:ti,ab OR ‘disease management’/exp OR ‘clinical management’:ti,ab OR ‘health care utilization’/exp OR ‘emergency department’/exp OR ‘primary care’/exp OR ‘primary care visit+1’:ti,ab)	((MeSH descriptor: [Asthma] explode all trees) OR “Allergic asthma”:ti,ab,kw OR “asthma allergic”:ti,ab,kw OR “suspected asthma”:ti,ab,kw) AND ((MeSH descriptor: [Spirometry] explode all trees) OR spirometr+1:ti,ab,kw OR “Pulmonary Function Test+1”:ti,ab,kw OR “bronchodilator test”:ti,ab,kw OR “bronchodilator response”:ti,ab,kw OR post0bronchodilator:ti,ab,kw OR “BDR test”:ti,ab,kw) AND ((MeSH descriptor: [Diagnosis] explode all trees) OR diagnos+1:ti,ab,kw OR “diagnostic accuracy”:ti,ab,kw OR (MeSH descriptor: [Disease Management] explode all trees) OR “clinical management”:ti,ab,kw OR “healthcare utilization”:ti,ab,kw OR “emergency department”:ti,ab,kw OR (MeSH descriptor: [Emergency Service, Hospital] explode all trees) OR “primary care visits”:ti,ab,kw) AND ((MeSH descriptor: [Pediatrics] explode all trees) OR child:ti,ab,kw OR adolescent:ti,ab,kw OR pediatric:ti,ab,kw OR paediatric:ti,ab,kw)
Allergic Asthma, PICO 4	(“Asthma”[MeSH] OR “Allergic asthma” OR allergic asthma[tiab] OR “wheezing”[tiab] OR “suspected asthma”[tiab]) AND (“Spirometry”[MeSH] OR spirometr+1[tiab] OR “Forced Expiratory Volume”[MeSH] OR FEV1[tiab] OR FEV1/FVC[tiab]) AND (“Nitric Oxide”[MeSH] OR “Exhaled Nitric Oxide”[tiab] OR FeNO[tiab] OR “fractional exhaled nitric oxide”[tiab]) AND (diagnosis[MeSH] OR diagnos+1[tiab] OR “diagnostic accuracy”[tiab] OR sensitivity[tiab] OR specificity[tiab] OR ROC[tiab] OR “predictive value”[tiab]) AND (therapy[tiab] OR “treatment decision”[tiab] OR “decision0making”[tiab] OR “controller therapy”[tiab] OR “maintenance therapy”[tiab] OR “as0needed therapy”[tiab] OR “ICS”[tiab]) AND (“Pediatrics”[MeSH] OR child[tiab] OR adolescent[tiab] OR pediatric[tiab] OR paediatric[tiab])	(‘asthma’/exp OR ‘allergic asthma’/exp OR ‘allergic asthma’:ti,ab OR wheezing:ti,ab OR ‘suspected asthma’:ti,ab) AND (‘spirometry’/exp OR spirometr+1:ti,ab OR ‘forced expiratory volume’/exp OR fev1:ti,ab OR ‘fev1/fvc’:ti,ab) AND (‘nitric oxide’/exp OR ‘exhaled nitric oxide’/exp OR ‘exhaled nitric oxide’:ti,ab OR feno:ti,ab OR ‘fractional exhaled nitric oxide’:ti,ab) AND (‘diagnosis’/exp OR diagnos+1:ti,ab OR ‘diagnostic accuracy’:ti,ab OR sensitivity:ti,ab OR specificity:ti,ab OR roc:ti,ab OR ‘predictive value’:ti,ab) AND (therapy:ti,ab OR ‘treatment decision’:ti,ab OR ‘decision making’/exp OR ‘decision0making’:ti,ab OR ‘controller therapy’:ti,ab OR ‘maintenance therapy’:ti,ab OR ‘as0needed therapy’:ti,ab OR ics:ti,ab)	((MeSH descriptor: [Asthma] explode all trees) OR “Allergic asthma”:ti,ab,kw OR allergic asthma:ti,ab,kw OR wheezing:ti,ab,kw OR “suspected asthma”:ti,ab,kw) AND ((MeSH descriptor: [Spirometry] explode all trees) OR spirometr+1:ti,ab,kw OR (MeSH descriptor: [Forced Expiratory Volume] explode all trees) OR FEV1:ti,ab,kw OR FEV1/FVC:ti,ab,kw) AND #3 (MeSH descriptor: [Nitric Oxide] explode all trees) OR “Exhaled Nitric Oxide”:ti,ab,kw OR FeNO:ti,ab,kw OR “fractional exhaled nitric oxide”:ti,ab,kw) AND ((MeSH descriptor: [Diagnosis] explode all trees) OR diagnos+1:ti,ab,kw OR “diagnostic accuracy”:ti,ab,kw OR sensitivity:ti,ab,kw OR specificity:ti,ab,kw OR ROC:ti,ab,kw OR “predictive value”:ti,ab,kw)
Rhinoconjunctivitis, PICO 1	(“Rhinitis, Allergic”[MeSH] OR “Rhinitis, Allergic, Seasonal”[MeSH] OR “Rhinitis, Allergic, Perennial”[MeSH] OR “Conjunctivitis, Allergic”[MeSH] OR rhinoconjunctivitis[tiab] OR “allergic rhinoconjunctivitis”[tiab] OR “allergic rhinitis”[tiab] OR “allergic rhinitis” OR “seasonal rhinitis” OR “rhinoconjunctivitis” OR “pollen allergy”) AND (“Allergy and Immunology”[MeSH] OR “Allergists”[MeSH] OR allergist+1[tiab] OR allergolog+1[tiab] OR specialist+1[tiab]) AND (“Allergens”[MeSH] OR allergen+1[tiab] OR trigger+1[tiab] OR “causative agent”[tiab]) AND (“Desensitization, Immunologic”[MeSH] OR “Allergen Immunotherapy”[tiab] OR “allergen0specific immunotherapy”[tiab] OR “specific immunotherapy”[tiab] OR AIT[tiab]) AND (“Quality of Life”[MeSH] OR “quality of life”[tiab] OR HRQoL[tiab] OR “quality of life”) AND (“Child”[MeSH] OR “Adolescent”[MeSH] OR child+1[tiab] OR adolescent+1[tiab] OR pediatric+1[tiab] OR paediatric+1[tiab])	(‘rhinoconjunctivitis’/exp OR ‘rhinoconjunctivitis’ OR ‘allergic rhinitis’ OR ‘pollen allergy’) AND (‘allergy’ OR ‘immunologist’ OR ‘immunology’ OR (allergy AND ‘immunology’)) AND (‘allergen’ OR ‘trigger’ OR) AND (‘desensitization’ OR ‘specific immunotherapy’ OR ‘immunotherapy’) AND (‘quality of life’ OR ‘quality of life assessment’ OR ‘Quality of Life Enjoyment and Satisfaction Questionnaire’) AND ([adolescent]/lim OR [child]/lim OR [infant]/lim)	((MeSH descriptor: [Rhinitis, Allergic] explode all trees) OR (MeSH descriptor: [Rhinitis, Allergic, Seasonal] explode all trees) OR (MeSH descriptor: [Rhinitis, Allergic, Perennial] explode all trees) OR (MeSH descriptor: [Conjunctivitis, Allergic] explode all trees) OR rhinoconjunctivitis:ti,ab,kw OR “allergic rhinoconjunctivitis”:ti,ab,kw OR “allergic rhinitis”:ti,ab,kw OR “seasonal rhinitis”:ti,ab,kw OR “pollen allergy”:ti,ab,kw) AND ((MeSH descriptor: [Allergy and Immunology] explode all trees) OR (MeSH descriptor: [Allergists] explode all trees) OR allergist+1:ti,ab,kw OR allergolog+1:ti,ab,kw OR specialist+1:ti,ab,kw) AND ((MeSH descriptor: [Allergens] explode all trees) OR allergen+1:ti,ab,kw OR trigger+1:ti,ab,kw OR “causative agent”:ti,ab,kw) AND ((MeSH descriptor: [Desensitization, Immunologic] explode all trees) OR “Allergen Immunotherapy”:ti,ab,kw OR “allergen0specific immunotherapy”:ti,ab,kw OR “specific immunotherapy”:ti,ab,kw OR AIT:ti,ab,kw) AND ((MeSH descriptor: [Child] explode all trees) OR (MeSH descriptor: [Adolescent] explode all trees) OR child+1:ti,ab,kw OR adolescent+1:ti,ab,kw OR pediatric+1:ti,ab,kw OR paediatric+1:ti,ab,kw)
Rhinoconjunctivitis, PICO 2	(“Rhinitis, Allergic”[MeSH] OR “Rhinitis, Allergic, Seasonal”[MeSH] OR “Rhinitis, Allergic, Perennial”[MeSH] OR “Conjunctivitis, Allergic”[MeSH] OR rhinoconjunctivitis[tiab] OR “allergic rhinoconjunctivitis”[tiab] OR “allergic rhinitis”[tiab] OR “allergic rhinitis” OR “seasonal rhinitis” OR “rhinoconjunctivitis” OR “pollen allergy”) AND (“Skin Tests”[MeSH] OR “skin prick test”[tiab] OR “prick test”[tiab] OR “skin prick”[tiab]) AND (“Immunoglobulin E”[MeSH] OR “specific IgE”[tiab] OR sIgE[tiab] OR RAST[tiab] OR ImmunoCAP[tiab]) AND (“Allergens”[MeSH] OR allergen+1[tiab] OR trigger+1[tiab] OR “causative allergen”[tiab]) AND (“Desensitization, Immunologic”[MeSH] OR “Allergen Immunotherapy”[tiab] OR “allergen0specific immunotherapy”[tiab] OR “specific immunotherapy”[tiab] OR AIT[tiab]) AND (“Child”[MeSH] OR “Adolescent”[MeSH] OR child+1[tiab] OR adolescent+1[tiab] OR pediatric+1[tiab] OR paediatric+1[tiab])	(‘rhinoconjunctivitis’/exp OR ‘rhinoconjunctivitis’ OR ‘allergic rhinitis’ OR ‘pollen allergy’) AND (‘skin test’ OR ‘patch test’ OR ‘prick test’) AND (‘immunoglobulin e’ OR ‘radioallergosorbent test’ OR ‘allergy rapid test’ OR ‘component resolved diagnosis’)AND (‘allergen’ OR ‘trigger’) AND (‘desensitization’ OR ‘specific immunotherapy’ OR ‘immunotherapy’) AND (‘quality of life’ OR ‘quality of life assessment’ OR ‘Quality of Life Enjoyment and Satisfaction Questionnaire’) AND ([adolescent]/lim OR [child]/lim OR [infant]/lim)	((MeSH descriptor: [Rhinitis, Allergic] explode all trees) OR (MeSH descriptor: [Rhinitis, Allergic, Seasonal] explode all trees) OR (MeSH descriptor: [Rhinitis, Allergic, Perennial] explode all trees) OR (MeSH descriptor: [Conjunctivitis, Allergic] explode all trees) OR rhinoconjunctivitis:ti,ab,kw OR “allergic rhinoconjunctivitis”:ti,ab,kw OR “allergic rhinitis”:ti,ab,kw OR “seasonal rhinitis”:ti,ab,kw OR “pollen allergy”:ti,ab,kw) AND ((MeSH descriptor: [Skin Tests] explode all trees) OR “skin prick test”:ti,ab,kw OR “prick test”:ti,ab,kw OR “skin prick”:ti,ab,kw) AND ((MeSH descriptor: [Immunoglobulin E] explode all trees) OR “specific IgE”:ti,ab,kw OR sIgE:ti,ab,kw OR RAST:ti,ab,kw OR ImmunoCAP:ti,ab,kw) AND ((MeSH descriptor: [Allergens] explode all trees) OR allergen+1:ti,ab,kw OR trigger+1:ti,ab,kw OR “causative allergen”:ti,ab,kw) AND ((MeSH descriptor: [Desensitization, Immunologic] explode all trees) OR “Allergen Immunotherapy”:ti,ab,kw OR “allergen0specific immunotherapy”:ti,ab,kw OR “specific immunotherapy”:ti,ab,kw OR AIT:ti,ab,kw) AND ((MeSH descriptor: [Child] explode all trees) OR (MeSH descriptor: [Adolescent] explode all trees) OR child+1:ti,ab,kw OR adolescent+1:ti,ab,kw OR pediatric+1:ti,ab,kw OR paediatric+1:ti,ab,kw)
Rhinoconjunctivitis, PICO 3	(“Rhinitis, Allergic”[MeSH] OR “Rhinitis, Allergic, Seasonal”[MeSH] OR “Rhinitis, Allergic, Perennial”[MeSH] OR “Conjunctivitis, Allergic”[MeSH] OR rhinoconjunctivitis[tiab] OR “allergic rhinoconjunctivitis”[tiab] OR “allergic rhinitis”[tiab] OR “allergic rhinitis” OR “seasonal rhinitis” OR “rhinoconjunctivitis” OR “pollen allergy”) AND (“Immunoglobulin E”[MeSH] OR “Allergens”[MeSH] OR “allergen components”[tiab] OR “molecular allergy diagnosis”[tiab] OR “component0resolved diagnosis”[tiab] OR “component resolved diagnosis”[tiab] OR “CRD”[tiab]) AND (“Immunoglobulin E”[MeSH] OR “specific IgE”[tiab] OR sIgE[tiab] OR RAST[tiab] OR ImmunoCAP[tiab]) AND (“Cross Reactions”[MeSH] OR cross0react+1[tiab] OR “cross reactivity”[tiab] OR “cross0reactive”[tiab] OR “cross0sensitization”[tiab]) AND (“Desensitization, Immunologic”[MeSH] OR “Allergen Immunotherapy”[tiab] OR “allergen0specific immunotherapy”[tiab] OR “specific immunotherapy”[tiab] OR AIT[tiab]) AND(“Child”[MeSH] OR “Adolescent”[MeSH] OR child+1[tiab] OR adolescent+1[tiab] OR pediatric+1[tiab] OR paediatric+1[tiab])	(‘rhinoconjunctivitis’/exp OR ‘rhinoconjunctivitis’ OR ‘allergic rhinitis’ OR ‘pollen allergy’) AND (‘skin test’ OR ‘patch test’ OR ‘prick test’) AND (‘immunoglobulin e’ OR ‘radioallergosorbent test’ OR ‘allergy rapid test’ OR ‘component resolved diagnosis’) AND (‘allergen’ OR ‘trigger’) AND (‘cross reaction’ OR ‘cross allergy’) AND (‘desensitization’ OR ‘specific immunotherapy’ OR ‘immunotherapy’) AND (‘quality of life’ OR ‘quality of life assessment’ OR ‘Quality of Life Enjoyment and Satisfaction Questionnaire’) AND ([adolescent]/lim OR [child]/lim OR [infant]/lim)	((MeSH descriptor: [Rhinitis, Allergic] explode all trees) OR (MeSH descriptor: [Rhinitis, Allergic, Seasonal] explode all trees) OR (MeSH descriptor: [Rhinitis, Allergic, Perennial] explode all trees) OR (MeSH descriptor: [Conjunctivitis, Allergic] explode all trees) OR rhinoconjunctivitis:ti,ab,kw OR “allergic rhinoconjunctivitis”:ti,ab,kw OR “allergic rhinitis”:ti,ab,kw OR “seasonal rhinitis”:ti,ab,kw OR “pollen allergy”:ti,ab,kw) AND ((MeSH descriptor: [Immunoglobulin E] explode all trees) OR (MeSH descriptor: [Allergens] explode all trees) OR “allergen components”:ti,ab,kw OR “molecular allergy diagnosis”:ti,ab,kw OR “component0resolved diagnosis”:ti,ab,kw OR “component resolved diagnosis”:ti,ab,kw OR CRD:ti,ab,kw) AND ((MeSH descriptor: [Immunoglobulin E] explode all trees) OR “specific IgE”:ti,ab,kw OR sIgE:ti,ab,kw OR RAST:ti,ab,kw OR ImmunoCAP:ti,ab,kw) AND ((MeSH descriptor: [Cross Reactions] explode all trees) OR cross0react+1:ti,ab,kw OR “cross reactivity”:ti,ab,kw OR “cross0reactive”:ti,ab,kw OR “cross0sensitization”:ti,ab,kw) AND ((MeSH descriptor: [Desensitization, Immunologic] explode all trees) OR “Allergen Immunotherapy”:ti,ab,kw OR “allergen0specific immunotherapy”:ti,ab,kw OR “specific immunotherapy”:ti,ab,kw OR AIT:ti,ab,kw) AND ((MeSH descriptor: [Child] explode all trees) OR (MeSH descriptor: [Adolescent] explode all trees) OR child+1:ti,ab,kw OR adolescent+1:ti,ab,kw OR pediatric+1:ti,ab,kw OR paediatric+1:ti,ab,kw)
Vernal Keratoconjunctivitis, PICO 1	(“vernal keratoconjunctivitis” OR “vernal” OR “Conjunctivitis, Allergic”[MeSH] OR “Keratoconjunctivitis”[MeSH] OR “vernal keratoconjunctivitis”[tiab] OR “VKC”[tiab] OR “allergic keratoconjunctivitis”[tiab] OR “atopic keratoconjunctivitis”[tiab]) AND (“Ophthalmology”[MeSH] OR “Ophthalmologists”[MeSH] OR ophthalmolog*[tiab] OR “Allergy and Immunology”[MeSH] OR allergolog*[tiab] OR specialist*[tiab]) AND (“primary care” OR “primary health” OR “Primary Health Care”[MeSH] OR “pediatrician”[tiab] OR “primary care”[tiab] OR “general pediatric practice”[tiab] OR “clinical assessment”[tiab]) AND (“Child”[MeSH] OR “Adolescent”[MeSH] OR child*[tiab] OR adolescent*[tiab] OR pediatric[tiab] OR paediatric[tiab])	(‘vernal conjunctivitis’/exp OR ‘vernal conjunctivitis’ OR ‘allergic conjunctivitis’ OR ‘keratoconjunctivitis’ OR “’atopic conjunctivitis’) AND (‘ophthalmology’ OR ‘ophthalmologist’) AND (‘primary medical care’ OR ‘primary health care’ OR ‘pediatrician’ OR ‘clinical assessment’ OR ‘therapy’ OR ‘drug therapy’) AND ([adolescent]/lim OR [child]/lim)	((MeSH descriptor: [Conjunctivitis, Allergic] explode all trees) OR (MeSH descriptor: [Keratoconjunctivitis] explode all trees) OR “vernal keratoconjunctivitis”:ti,ab,kw OR VKC:ti,ab,kw OR vernal:ti,ab,kw OR “allergic keratoconjunctivitis”:ti,ab,kw OR “atopic keratoconjunctivitis”:ti,ab,kw) AND ((MeSH descriptor: [Ophthalmology] explode all trees) OR (MeSH descriptor: [Ophthalmologists] explode all trees) OR ophthalmolog*:ti,ab,kw OR (MeSH descriptor: [Allergy and Immunology] explode all trees) OR allergolog*:ti,ab,kw OR specialist*:ti,ab,kw) AND ((MeSH descriptor: [Primary Health Care] explode all trees) OR “primary care”:ti,ab,kw OR “primary health”:ti,ab,kw OR pediatrician:ti,ab,kw OR “general pediatric practice”:ti,ab,kw OR “clinical assessment”:ti,ab,kw) AND ((MeSH descriptor: [Child] explode all trees) OR (MeSH descriptor: [Adolescent] explode all trees) OR child*:ti,ab,kw OR adolescent*:ti,ab,kw OR pediatric:ti,ab,kw OR paediatric:ti,ab,kw)
Vernal Keratoconjunctivitis, PICO 1	(“Conjunctivitis, Allergic”[MeSH] OR “Keratoconjunctivitis”[MeSH] OR vernal keratoconjunctivitis[tiab] OR VKC[tiab] OR “vernal keratoconjunctivitis”[tiab]) AND (“Skin Tests”[MeSH] OR “Prick Test”[tiab] OR “Skin prick”[tiab] OR prick test[tiab] OR intradermal[tiab] OR “intradermal test”[tiab]) AND (“Immunoglobulin E”[MeSH] OR “specific IgE”[tiab] OR sIgE[tiab] OR “RAST”[tiab] OR “ImmunoCAP”[tiab]) AND (trigger*[tiab] OR allergen*[tiab] OR “allergen identification”[tiab] OR diagnost*[tiab] OR diagnosis[MeSH] OR management[tiab] OR therap*[tiab]) AND (“Child”[MeSH] OR “Adolescent”[MeSH] OR child[tiab] OR adolescent[tiab] OR pediatric[tiab] OR paediatric[tiab])	(‘vernal conjunctivitis’/exp OR ‘vernal conjunctivitis’ OR ‘allergic conjunctivitis’ OR ‘keratoconjunctivitis’ OR “’atopic conjunctivitis’) AND (‘skin test’ OR ‘patch test’ OR ‘prick test’ OR ‘allergy skin test device’) AND (‘immunoglobulin’ OR ‘immunoglobulin e’ OR ‘radioallergosorbent test’ OR ‘allergy rapid test’) AND (‘trigger’ OR ‘allergen’ OR ‘diagnosis’ OR ‘management’ OR ‘diagnostic procedure’ OR) AND ([adolescent]/lim OR [child]/lim)	((MeSH descriptor: [Conjunctivitis, Allergic] explode all trees) OR (MeSH descriptor: [Keratoconjunctivitis] explode all trees) OR “vernal keratoconjunctivitis”:ti,ab,kw OR VKC:ti,ab,kw) AND ((MeSH descriptor: [Skin Tests] explode all trees) OR “prick test”:ti,ab,kw OR “skin prick”:ti,ab,kw OR intradermal:ti,ab,kw OR “intradermal test”:ti,ab,kw) AND ((MeSH descriptor: [Immunoglobulin E] explode all trees) OR “specific IgE”:ti,ab,kw OR sIgE:ti,ab,kw OR RAST:ti,ab,kw OR ImmunoCAP:ti,ab,kw) AND (trigger*:ti,ab,kw OR allergen*:ti,ab,kw OR “allergen identification”:ti,ab,kw OR diagnost*:ti,ab,kw OR (MeSH descriptor: [Diagnosis] explode all trees) OR management:ti,ab,kw OR therap*:ti,ab,kw) AND ((MeSH descriptor: [Child] explode all trees) OR (MeSH descriptor: [Adolescent] explode all trees) OR child:ti,ab,kw OR adolescent:ti,ab,kw OR pediatric:ti,ab,kw OR paediatric:ti,ab,kw)

* stands for singular or plural.

**Table A2:** Individual characteristics of included studies. Note: C = cohort study, CC = case–control study, M = multicenter study, P = prospective design, R = retrospective design, S = single-center study, RCT = randomized controlled trial.

**Original Studies**										
**AUTHOR**	**YEAR**	**Type of Study**	**OBJECTIVES**	**POPULATION (GENERAL CHARACTERISTICS)**	**SAMPLE SIZE**	**OUTCOME**	**RESULTS** **(Quantitative Elements)**	**RESULTS (Description)**	**LIMITATIONS**	**PICO**
Allergic Asthma										
Hansen S et al. [22]	2023	C, R, M	Examine the prevalence of severe asthma and the proportion of severe asthma in patients followed by specialist centers.	Children and adults with severe asthma (6–17 years and ≥18 years)	598,242 children and adults	Percentage of children with severe asthma and two or more asthma-related exacerbations/emergency department visits and admissions. Percentage of children with severe asthma/mild-moderate asthma/uncontrolled asthma. Percentage of children with severe asthma and two or more exacerbations and contact with specialist care.	- The proportion of children with severe asthma who had two or more exacerbations ranged from 14% in Norway to 37% in Sweden and Finland. - In children with severe asthma, 2–10% and 4–5%, respectively, had asthma-related emergency department visits and admissions. - Uncontrolled asthma (defined as at least two OCS dispensations/asthma-related emergency department visits and/or one asthma hospitalization and/or high SABA use) in children with severe asthma is 70% in Sweden, 53% in Finland and 55% in Norway. - In patients with mild to moderate asthma, uncontrolled asthma was between 12 and 24% in children. - In children with severe asthma and two or more exacerbations, the percentages of patients with specialist contact were 56% and 41%, respectively, in Sweden and Finland.	- The proportion of children with severe asthma and two or more exacerbations ranged from 14% in Norway to 37% in Sweden and Finland. - In children with severe asthma, less than 10% had asthma-related emergency room visits and admissions. - Uncontrolled asthma in children with severe asthma is almost 2/3 in Sweden and just over half in Finland and Norway. - In patients with mild-moderate asthma, uncontrolled asthma involves about 1/4 of children. - In children with severe asthma and two or more exacerbations, more than half have contacted a specialist in Sweden and less than 50% in Finland.	Heterogeneous population Unclear conduction follow-up	1
Nwaru BI et al. [18]	2019	C, R, M	To describe the use of SABA over an 8-year period in a national cohort of asthmatic patients in Sweden.	Patients aged 12–45 years who have received at least two prescriptions for asthma medication within 12 months.	365,324 patients	To evaluate the demographic and clinical determinants of SABA abuse, and investigate associations between SABA abuse and risk of exacerbation, all-cause mortality, and respiratory-related death.	- 30% of the total made excessive use of SABA - in the population 12–17 years: 27.2% 0–2 cans, 32% 3–5 cans, 24.9% 6–10 cans and 13.9% ≥11 cans.	Increasing the number of SABA containers collected was associated with an increased risk of exacerbations in a dose-dependent manner. The mortality rate increased with the increase in the number of SABA containers collected.	Use of medication collection for obstructive pulmonary disease in pharmacies as an indicator for the diagnosis and treatment of asthma, as well as the lack of access to primary care data, from which the diagnosis of asthma can usually be gleaned. The use of SABA was based on collected prescriptions, which may not fully reflect the actual use of the drugs by patients.	1
Hakizimana A et al. [27]	2025	C, P, S	Evaluate the feasibility and effectiveness of hubs in providing post-acute attack reassessments, provide asthma training, the opportunity to perform diagnostic pulmonary function tests, and optimize treatment.	Children aged 4 to 17 years referred to the hub with uncontrolled asthma or post-acute attack.	312 patients	To assess the number of critically ill children who need further emergency care and the proportion of children diagnosed with asthma according to NICE guidelines.	- 241/266 (90.6%) with acceptable spirometry, of which 27.3% basal spirometry was abnormal: 60% low FEV 1, 87.9% low FEV 1/FVC ratio and 39.4% low FEV 1 and FEV 1/FVC. - 125/266 (46.9%) with acceptable FeNO measurements; Median FeNO was 20 ppb; 40% FeNO ≥25 ppb and 29.6% FeNO ≥35 ppb. - 231 (96%) performed post-bronchodilator spirometry and 31.6% with BDR ≥12%. These coincide with 27.4% of all CYPs who visited the asthma center and in whom asthma was confirmed in a single visit.	Pediatric asthma centers represent a viable model of care for providing children with post-asthma attack check-ups and identifying high-risk patients who need further treatment. Spirometry, BDR and FeNO tests have allowed diagnostic confirmation in a significant percentage of children.	Possible variability in the methodology for evaluating functional parameters.	1
Paskaradevan J et al. [26]	2022	C, R, S	To describe the clinical care of children with critical asthma admitted to a single PICU center and determine whether pulmonary counseling during hospitalization had an impact on outcomes.	Children with critical asthma admitted to PICU (age 4–18 years).	179 patients	Patients were stratified into two groups: patients who received specialist asthma counseling during hospitalization and patients who did not. The primary outcomes were: total hospital stay, total pediatric ICU stay, treatment modality, and control drug dose escalation prior to PICU discharge. Secondary outcomes were: scheduled follow-up with the pulmonologist, participation in scheduled follow-up, emergency department visits, hospitalizations, and systemic steroid cycles in the 12 months following discharge.	- 80 patients (44.7%) had pulmonary counseling. - In the group that received pulmonary counseling compared to those who did not, there was a significant difference in hospital stay (4.16 vs. 2.86 days, *p* < 0.0001) and PICU stay (2.00 vs. 1.00 days, *p* < 0.0001), in the increase in control drug therapy (66% vs. 21%, *p* < 0.0001), in scheduled outpatient follow-up at the pulmonary clinic (87.5% vs. 45.4%, *p* < 0.0001) and in the administration of ≥3 cycles of systemic steroids in the 12 months following discharge (32.2% vs. 14.7%, *p* = 0.02).	In the pulmonology counseling group compared to the non-pulmonology counseling group, there was a significant difference in hospital length of stay and length of PICU stay, an increase in control medication dose, scheduled outpatient pulmonary follow-up, and administration of ≥3 cycles of systemic steroids in the 12 months following discharge.	Single center, small study population size, short follow-up duration after discharge, and difficulty in monitoring follow-up and care outside the health care system.	1
Rosman Y et al. [28]	2021	C, R, S	To evaluate the effect of visiting an asthma specialist on asthma control in children.	Children (5–16 years) who have received a diagnosis of asthma during the study period (2000–2016) and who have had at least one visit to an asthma specialist.	13,533 patients	Comparison of asthma outcomes 2 years before and 2 years after a specialist visit. These included systemic steroid prescriptions, inhaled steroids (single or combined inhalers), and short-acting beta-agonists. Data on medical visits (general practitioners and emergency rooms) and hospital admissions were also compared.	- Higher number of visits to the general practitioner in the period prior to the specialist visit (respectively 4.4 ± 4.4 vs. 3.16 ± 3.9 visits; *p* < 0.01). After specialist visit: - Lower average number of visits to the emergency department (0.52 ± 1.3 vs. 0.45 ± 1, *p* < 0.01). - Lower number of hospital admissions for all causes (0.13 ± 0.45 vs. 0.08 ± 0.4, *p* < 0.01). - Lower number of hospital admissions for asthma exacerbations (0.08 ± 0.345 vs. 0.05 ± 0.3, *p* < 0.01). - Lower prescription of short-acting beta-agonists (2.85 ± 3.6 vs. 2.2 ± 3.7, *p* < 0.01). - Higher prescriptions of inhaled steroids (1.9 ± 2.9 vs. 2.7 ± 3.7, *p* < 0.01). - Reduction in corticosteroid prescription (0.81 ± 1.9 vs. 0.43 ± 1.4, *p* < 0.01).	After the specialist’s visit, the average number of visits to emergency departments, all-cause hospital admissions, and hospital admissions for asthma exacerbations decreased. The prescription of short-acting beta-agonists decreased and the prescriptions of inhaled steroids increased.	- Diagnosis of asthma based on the information reported in the medical record and not according to the guidelines—failure to divide the population according to asthma severity—not all asthmatic patients have been referred to the specialist.	1
Bardach NS et al. [29]	2022	C, R, S	To evaluate the association between follow-up after an asthma-related emergency department (ED) visit and the likelihood of subsequent asthma-related emergency department use.	Patients between 3 and 12 years of age with an asthma-related emergency department visit in the year under consideration.	90,267 patients	The outcome of interest was asthma-related subsequent emergency department visits after the index emergency department visit. Three binary indicators were used (any subsequent emergency department visit within 60 days, 365 days, or until the end of available follow-up) and as a continuous indicator (number of subsequent emergency department visits during follow-up/100 days/child). Subsequent visits within 14 days following the index ER visit were not included in any of the outcome definitions.	- 93.2% with at least 365 days of follow-up data. - Follow-up within 14 days with a primary care physician or specialist in 22.6% (*n* = 20,384). - Among follow-up visits: 70.9% with a pediatrician, 17.0% with a general practitioner, 9.2% with an internist, and 3.0% with a pulmonologist or allergist/immunologist. Patients with follow-up visits within 14 days: - Lower frequency of subsequent asthma-related emergency department visits both within 60 days (5.7% vs. 6.4%, *p* < 0.001) and within 365 days (25.0% vs. 28.3%, *p* < 0.001). - Lower rates of subsequent asthma-related emergency department visits over time (adjusted analysis: 66.7 visits/100 child-years versus 77.3 visits/100 child-years for those without a follow-up visit, *p* < 0.001).	14-day follow-up was associated with a reduction in asthma-related follow-up visits to the emergency department at 60 days and at 365 days. Similarly, a 14-day follow-up was associated with a reduction in the rate of repeat subsequent visits to the emergency department.	- No causality attributed to the association. - No difference in asthma severity. - Potential bias that patients with more severe asthma were more likely to receive a follow-up visit.	1
Bender B et al. [31]	2000	C, R, S	Compare four methods of assessing adherence: infant report, mother’s report, can weight, and electronic measurements of measured dose inhaler activation (MDI).	27 children with mild-moderate asthma, prospectively followed for 6 months; each child was using a multi-dose inhaler (MDI) equipped with an electronic dispenser connected to their inhaled steroid.	27 children	At each bimonthly follow-up visit, data on the weight of the dispenser and the can were recorded, while the child and mother were interviewed separately about the use of the drug.	- Adherence to inhaled steroid > to 80% reported by children and mothers. - The weight of the can revealed, on average, an adherence of 69%. - The average adherence recorded by the electronic dispenser was 50%.	Electronic monitoring of therapeutic adherence was significantly more accurate than self-reported data or container weight measurements.	Heterogeneity in evaluation parameters. Doubtful inter-operator consistency and homogeneity of the studied population.	1
Butz AM et al. [30]	2015	DC, P, S	To examine factors associated with high SABA use in asthmatic children living in urban centers.	Children (3–12 years) living in urban areas with persistent asthma were enrolled during an emergency department visit for the treatment of an acute asthma exacerbation.	100 children	The primary outcome: frequency of use of SABA during the 12 months prior to the emergency department enrollment visit and was based on pharmacy records. All children underwent allergen-specific IgE serology testing and saliva testing for cotinine during the ED enrollment visit. Medical records for the last 12 months were obtained. The number of SABA refills over the past 12 months has been divided into low-moderate use and high-use groups. The SABA groups were compared based on the number of days and nights of symptoms, allergen sensitization and exposures.	- 27% of patients reported daily use of SABA in the past 2 weeks. - 3.12 (SD 2.8) is the average number of SABA recharges in 12 months, with a range of 0–18 recharges. - A quarter of children had 4 or more SABA refills in 12 months and 16% had 6 or more SABA refills in the same time period. - An average of emergency department visits in the last 3 months of 1.37 (SD 1.0) and an average of hospital admissions of 0.12 (SD 0.4) in the same period of time. - 31% had a confirmed hospitalization for asthma in intensive care over the course of their lifetime. - The majority of caregivers rated their child’s asthma control as well controlled (51%). - SABA users with a high number of doses were 5 times more likely to be hospitalized for asthma, almost 3 times more likely to be admitted to the ICU for asthma, and more than 3 times more likely to have received prior specialist asthma care or to have positive cockroach sensitization compared to SABA users with a low to moderate number.	- Positive sensitization to cockroaches was associated with an almost seven-fold increased risk of high SABA use. - Excessive use of SABA is associated with increased use of healthcare. 15 A 5-fold higher hospitalization rate was observed in the last 3 months and an almost 3-fold higher rate of prior ICU admission was observed in children with high SABA use compared to children with low to moderate SABA use.	- The use of pharmacy claims data for SABA refills is not a direct measure of SABA use, as leakage and/or sharing of dispensed containers may occur. - A high number of SABA refills may reflect the sharing of medicines among family members and may have led to an overestimation of SABA refills. - The sample of children with severe asthma may have greater uncontrolled asthma than the general pediatric population with asthma.	1
Erickson S et al. [37]	2005	C, P, S	To evaluate the longitudinal impact of asthma specialist care on the risk of emergency department (ED) visits and asthma hospitalization.	A cohort of adults belonging to a health care organization, hospitalized for asthma.	4742 patients	Two groups of subjects were identified: adults admitted to the intensive care unit (ICU) for asthma and patients admitted without ICU admission. For each subject, the use of drugs and health care in the 12 months prior to hospitalization was retrospectively ascertained. After hospital discharge, a subset of adults with asthma was recruited to undergo structured telephone interviews that assessed sociodemographic characteristics, asthma history, and health status.	- 728 adults were treated by an asthma specialist during the year prior to initial hospital admission (15.4%; 95% confidence interval [CI] 14.3–16.4%); of these, they were allergists (9.5%; 95% CI 8.7–10.4%), pulmonologists (5.0%; 95% CI 4.4–5.6%) and both allergists and pulmonologists (0.86%; 95% CI 0.62–1.18%). - Greater severity of asthma among subjects who had undergone both allergy and pneumological examinations (mean score 15.7 points), followed by those who had only pneumological examinations (mean score 14.7 points) and only allergy examinations (mean score 13.9 points) (*p* = 0.04). Asthma severity scores were lower among those who had not consulted an asthma specialist (mean score 13.1 points). - Specialist visits for asthma were associated with a higher likelihood of dispensing all classes of asthma medication (*p* < 0.001).	Most of the specialists contacted by patients were pulmonologists, allergists and in a few cases both. The severity of asthma was more severe among those who had contacted both allergists and pulmonologists. Specialist visits were associated with a higher prescription of asthma drugs of any category.	Questionable congruity of inter-operator observations. Heterogeneity of assessment by specialists.	1
Frey SM et al. [36]	2016	C, P, M	Describe the actions taken by clinicians at primary care visits to promote daily use of preventive asthma medications and determine if patient- or encounter-related variables are associated with receiving asthma medication training.	Children (2–12 years) with asthma.	319	Caregivers were contacted by telephone shortly after the meeting (within 14 days) and were asked to characterize asthma-specific actions that occurred during the meeting by answering questions from a structured interview tool. They reported whether the healthcare provider asked targeted questions to assess the severity or control of the child’s asthma (e.g., frequency of daytime symptoms, nighttime symptoms, and use of rescue medications) and also whether the provider asked about current asthma medications or provided other information and preventive care for asthma recommended by the guidelines. Information regarding updates or changes to asthma medications and the child’s treatment plan was also collected.	- 42% of caregivers reported correct explanation of medications by the physician - 21.5% of caregivers received written plan for asthma - “Ideal teaching” 16.7% of all encounters; “Minimum expected teaching” 46.2% of visits—ideal teaching was greater during asthma visits than during follow-up visits (23.9% vs. 11.4%; *p* = 0.021) - Children seen during check-ups and for asthma, compared to children seen for other diseases, are more likely to. (a) receive a recommendation for an asthma-specific follow-up visit (42% vs. 23%, *p* = 0.007), (b) be questioned about the frequency of daytime symptoms (53.9% vs. 34.4%, *p* = 0.01), the frequency of nighttime symptoms (58.3% vs. 32.9%, *p* = 0.001), the use of rescue medication (60.5% vs. 41.4%, *p* = 0.015), and the asthma medications currently being taken (80.9% vs. 55.7%, *p* ≤ 0.001).	Less than half of all caregivers reported that their child’s doctor explained how to use asthma medication correctly. Fewer than one in four caregivers reported receiving a written asthma action plan. Caregivers reported “Ideal Teaching” (education on the correct use of medication and receiving an asthma action plan) in only 16.7% of all meetings; “Minimal expected teaching” (education on the proper use of medication or receiving an asthma action plan) was reported in 46.2% of visits. A comparison of caregivers’ responses to these items revealed no statistically significant differences between primary care and asthma visits compared to visits for other diseases. Although the limited sample size prevented an in-depth analysis of the subgroups, we nevertheless found that the ideal teaching occurred more commonly during asthma visits, even when compared to follow-up visits (23.9% vs. 11.4%, respectively; *p* = 0.021). Children seen during follow-up and asthma visits, compared to children seen for other conditions, were significantly more likely to receive a recommendation for an asthma-specific follow-up visit (42% vs. 23%, *p* = 0.007) and to be questioned about the frequency of daytime symptoms (53.9% vs. 34.4%, *p* = 0.01), the frequency of nighttime symptoms (58.3% vs. 32.9%, *p* = 0.001), the use of rescue medication (60.5% vs. 41.4%, *p* = 0.015) and asthma medication currently being taken (80.9% vs. 55.7%, *p* ≤ 0.001). However, more than one-third of caregivers seen during follow-up and asthma visits were not asked to report recent symptoms. In addition, less than half of these caregivers reported receiving a recommendation to schedule an asthma-specific follow-up visit. No significant differences were identified between the types of meetings with regard to the discussion of the correct use of drugs and the drafting of written action plans.	- results that are not widely generalizable. - Results evaluated only on the basis of the caregiver’s report.	1
Beck AF et al. [35]	2015	C, P, S	To describe allergen sensitization profiles and the distribution of self-reported home exposures among children hospitalized for asthma. To assess how sensitization profiles varied based on sociodemographic and clinical factors.	children, aged 4–16 years, hospitalized for an asthma exacerbation.	478 patients	The primary outcomes were sensitization to common indoor allergens, assessed using ImmunoCap (ARUP, Salt Lake City, UT, USA), a measure of allergen-specific serum IgE. Allergens tested included Alternaria alternata/A. tenuis, Aspergillus fumigatius, American cockroach, mouse epithelium, *Dermatophagoides pteronyssinus*, *Dermatophagoides farinae*, cat dandruff, and dog dandruff. By convention, tests were considered positive when IgE was at least 0.35 kU/L. Secondary outcomes included self-reported household environmental exposure.	- >50% sensitization to Alternaria, Aspergillus, D. pteronyssinus, D. farinae, cat dandruff and dog dandruff; 28% sensitized to cockroaches; 18.2% sensitized to mice - 91% sensitized to more than 1 allergen; 68% to three or more allergens - 17% presence of mold or fungi in your home; 12% presence of cockroaches. - 40% reported one or more adverse household exposures; 12% reported three or more. - 70% reported > presence of carpet in the home and 51% of furry pets.	More than 50% of the children were sensitized to each of Alternaria, Aspergillus, D. pteronyssinus, D. farinae, cat dandruff, and dog dandruff. Almost 30% were sensitized to cockroaches, while 18% were sensitized to mice. More than 91% of children were sensitized to one or more allergens tested; 68% to three or more. A total of 17% of respondents reported the presence of mold or fungi in their home; 12% reported the presence of cockroaches. More than 40% reported one or more adverse household exposures; 12% reported three or more. Over 70% reported the presence of carpet in the home and 51% of furry pets. The reported history of hospitalization was significantly associated with sensitization to Alternaria, Aspergillus, cockroaches, and dust mites (each *p* < 0.05).	Definition of asthma based on medical assessment Allergens tested mainly typical of indoor environments Difficult to generalize exposures measured by self-reporting.	1, 3
Sheares BJ et al. [34]	2015	RCT, P, M	Evaluate the effectiveness of written instructions in the form of a written asthma action plan given by medical specialists as part of regular asthma care during office visits.	Children and adults with persistent asthma.	407 subjects	Primary outcomes included (1) frequency of asthma symptoms, (2) emergency department visits, and (3) quality of life (QOL) of asthma. Using a 2-week booster period, the frequency of asthma symptoms was measured in three ways: (a) the average number of days with asthma symptoms, (b) the average number of nights with symptoms, and (c) the average number of days of use of short-acting bronchodilators. Asthma emergency department (ED) visits were assessed using a 3-month booster. Asthma QOL was measured at 6- and 12-month follow-ups using the Juniper Mini Asthma QOL Questionnaire (MiniAQLQ) (14) for adult participants and the Pediatric Asthma Caregivers QOL Questionnaire (PACQLQ).	In both groups, similar and significant reductions in the frequency of asthma symptoms (daytime symptoms [*p* < 0.0001], nocturnal symptoms [*p* < 0.0001], use of β-agonists [*p* < 0.0001]). Significant reduction in ED visits was found in the intervention (*p* < 0.0001) and control (*p* < 0.0006) groups. Significant improvement in quality of life scores for asthma was found in adults (*p* < 0.0001) and pediatric caregivers (*p* < 0.0001).	Both groups showed similar and significant reductions in the frequency of asthma symptoms (daytime symptoms [*p* < 0.0001], nocturnal symptoms [*p* < 0.0001], use of β-agonists [*p* < 0.0001]). A significant reduction in emergency department visits was found in the intervention (*p* < 0.0001) and control (*p* < 0.0006) groups. Significant improvement in quality of life scores for asthma was found in adults (*p* < 0.0001) and pediatric caregivers (*p* < 0.0001).	Involvement of specialist doctors only. The population does not represent the majority of those involved in allergy or pulmonology studies.	1
Diette GB et al. [33]	2001	C, P, M	To determine whether child care was more consistent with national asthma guidelines when the usual source of asthma care was a specialist rather than a general practitioner.	Parents of children with asthma (adults).	260 subjects	To define the usual source of care, parental data was used about the doctor who was primarily responsible for asthma care (specialist, general practitioner, or both) and who they would contact (specialist or general practitioner) with questions about asthma care. The consistency of care with the guidelines of the National Asthma Education and Prevention Program of 1997 was assessed, using 11 indicators in 4 areas of asthma care: patient education, control of factors contributing to asthma symptoms, periodic physiological assessment and monitoring, and correct use of medications.	the use of control drugs was greater in patients treated primarily by specialists than in general practitioners (94% vs. 72%, *p* < 0.01), as well as having written instructions for the management of asthma attacks (69% vs. 46%, *p* < 0.05), having been instructed in the use of inhalers (89% vs. 69%, *p* < 0.05) and having undergone pulmonary function tests (86% vs. 48%, *p* < 0.05). Daily use of control medications was reported in 68% of children whose parents always called a specialist, but only in 36% (P, 0.02) of cases where parents always called a general practitioner. Similarly, patients were more likely to have received instructions on how to use an inhaler (92% vs. 69%, P, 0.05) or to have had a lung function test performed (88% vs. 47%, P, 0.0001) when parents called a specialist rather than a general practitioner.	In all 4 domains, care was more likely consistent with guidelines when specialists were the usual source of care. The biggest differences between specialist and general practitioner management were the use of background medications, performing pulmonary function tests, and being informed about asthma triggers and how to avoid them.	Not all factors were considered that determine whether patients consulted an asthma specialist.	1
Yee AB et al. [38]	2013	C, P, S	Describe what National Heart Lung and Blood Institute preventive actions are taken for children with persistent asthma symptoms at the time of a primary care visit and determine how care delivery varies based on the severity of asthma symptoms.	Children (2–12 years) with critical asthma admitted to PICU.	171 children	Caregivers were interviewed by telephone within 2 weeks of the first visit about the specific preventive actions taken. Bivariate and regression analyses assessed the relationship between the severity of asthma symptoms and the actions taken during the visit.	Children with more severe asthma were significantly more likely (*p* = 0.01) to visit the doctor’s office for asthma than those with less severe symptoms (12% vs. 24% vs. 36% for persistent mild, moderate persistent, and severe persistent symptoms, respectively). Children with more severe asthma symptoms received more preventive actions during office visits, including administering preventive medications, receiving an asthma action plan, and recommending an asthma-specific follow-up visit, compared to those with milder symptoms (*p* < 0.05 for all). Discussion of medications, triggers, and how smoking worsens asthma was also significantly correlated with greater symptom severity (*p* < 0.05 for all). Children with mild persistent asthma symptoms were significantly less likely than those with moderate to severe persistent symptoms to receive preventive drug action (OR 0.34 [95%CI 0.14–0.84]), discussion of reduction in triggers (0.39 [0.19–0.82]), recommendation of follow-up for asthma (0.40 [0.19–0.87]), and receipt of an action plan (0.37 [0.16–0.86]).	Children with mild and persistent asthma symptoms were less likely to receive measures of preventive drug therapy, discussions about reducing triggers, follow-up recommendations, and receiving an action plan than those with more severe symptoms.	Limited sample. The evaluation of the outcomes was based on the caregiver’s report or on the documentation in the medical record.	1
Hauerslev, M. et al. [44]	2022	C, R, S	To investigate risk factors for loss of asthma control in well-controlled children with mild to moderate disease.	146 school-aged children with mild to moderate well-controlled asthma.	146 children	Exacerbations and hospital admissions.	27 children (18%) experienced 56 acute events defined as hospital admissions, emergency department accesses or outpatient management. The risk of acute events was increased in women (adjusted odds ratio, aOR = 2.4 (1.0–5.9), *p* = 0.047) and with a higher level of treatment according to GINA at baseline (aOR = 1.6 (1.1–2.5), *p* = 0.03). In addition, female sex (aOR = 6.1 (1.4–42.2), *p* = 0.01) and higher FeNO values (aOR = 1.8 (1.0–3.2), *p* = 0.04) were associated with prescription oral corticosteroids (OCS).	Female sex, higher FeNO levels, and a higher GINA treatment step increase the risk of long-term loss of control, including acute events and OCS use in apparently well-controlled children with mild to moderate asthma. These findings are important for primary care physicians and clinicians in outpatient asthma clinics to identify children at risk and plan for more frequent follow-ups.	Follow-up period: 5 years Well-controlled asthma.	2
Kallis et al. [43]	2023	CS, R, M	Determine factors associated with poor control and referral to secondary specialty care.	Patients between 6 and 17 years of age with poorly controlled asthma.	155,270 children		About 1 in 6 children or adolescents with active asthma do not achieve adequate symptom control.	Among children and adolescents with poorly controlled asthma, referral to specialist care is rare, especially in cases of mild asthma, although these patients are still at significant risk of adverse outcomes.		2
Keemink, Y.S et al. [46]	2015	CS, R, S	To investigate changes in adherence to inhaled corticosteroids before and after a scheduled office visit in young children included in a comprehensive asthma management program.	Asthmatic children (average age 4.8 years).	104 children		An increase in adherence >10% prior to the visit was observed in 17 patients (22%), but the median change in adherence around the visit was 0. No significant difference was found in the days before or after the visit (*p* > 0.2). The median change in adherence was 9%, with no significant differences between children with and without pre-visit increase (*p* = 0.12). Twelve patients (15.4%) showed an increase in adherence in the month following the visit.	“White coat adherence” was not observed in children included in a comprehensive asthma management program with high overall adherence. This suggests that WCA in chronic pediatric patients occurs primarily when overall adherence is low.		2
Schoo s T. et al. [89]	2020	CS, P, S	Explore the language used by pediatricians to encourage patient adherence to daily therapy.	7 outpatients (4–13 years) with chronic asthma and their caregivers.	7 children	n.a.	n.a.	Pediatricians with documented success in obtaining good adherence begin consultation with the patient’s agenda, answer questions, reassure about the effectiveness of treatment and seek explicit agreement on the therapeutic plan.	Language study on a few patients.	2
Nyenhuis et al. [47]	2020	CS, R, M	to analyze rates and relative contributions of comorbidities and care settings with respect to asthma severity and control in a large national cohort.	information de-identified from 28,508 unique encounters documented in the database “Asthma Specialist Tool to Help Manage Asthma and Improve Quality” children.	362,800	Access to pediatric asthma specialists.	Patients assessed by the general practitioner were on average older, with more severe asthma and less controlled asthma (*p* < 0.05). Patients followed by the specialist were also suffering from more frequent comorbidities (reflux disease, rhinosinusitis, obstructive apnea).	Smoke exposure (in children), rhinosinusitis, and the African American race were associated with severe illness and uncontrolled asthma.	Heterogeneity in the population studied and in the outcomes applied.	2
Håkansson, KEJ et al. [59]	2024	C, P, S	To quantify the reliability of risk factors associated with T2 and non-T2 phenotypes in a national cohort of children aged 2–17 years undergoing active asthma management.	Danish children and adolescents (2–17 years) with asthma on active treatment belonging to the REASSESS cohort.	29,851	Estimation of the prevalence of phenotypes and their association with disease control, risk of exacerbation and severity of asthma.	- Excessive use of SABA in 16% of children (25% in 2–5 years, 6.4% in 12+ years). - High IgE: 78% (among those with available data). Association with exacerbations: OR 1.41 (1.20–1.66). Association with severe asthma: OR 1.31 (1.08–1.60).	Elevated IgE levels were more frequent in children with severe asthma and those with exacerbations, and were associated with a higher likelihood of each of these outcomes, when adjusted for age and sex. Results were consistent for both elevated total IgE and positive specific IgE/RAST, which were analyzed separately in sensitivity analyses, except for the odds of severe asthma for children with elevated total IgE, which did not reach statistical significance.	Children who did not take ICS were excluded from the cohort, decreasing generalizability and limiting validity only for children treated with ICS. Possible selection bias due to the high rate of positive RAST in the present cohort.	3
Lejeune S et al. [75]	2024	C, R, M	To determine if sensitization patterns can discriminate between children with NSRW (non-severe recurrent wheezing)/NSA (non-severe asthma) and those with SRW (severe recurrent wheezing)/SA (severe asthma).	Population: children (3–12 years).	295 children (3–6 years nr. 125, 7–12 years nr. 170)	Children assigned to four groups: preschool non-severe recurrent wheezing (NSRW), severe preschool recurrent wheezing (SRW), non-severe school-age asthmatic children (NSA), and severe school-age asthmatic (AS) children. Atopy: at least one positive skin prick test and/or specific IgE (sIgE) levels (≥0.35 kuA/L) against airborne and/or food allergens. 112-component allergen IgE measured using an ImmunoCAP Immuno Solid-Phase Allergen Chip (ISAC).	Among 295 children: 47 NSRW, 78 SRW, 108 NSA, and 62 individual c-sIgE sensibilization (at least one positive c-sIgE ≥0.30 ISU) for 51.4% of preschool children and 75.3% of school-age children. Multisensitization rates were comparable between children with NSA and AS (62% vs. 61.3%, *p* = 0.92). No difference was found in airborne awareness profiles. Sensitization to lipid transfer protein (nsLTP) was more frequent among children with AS than those with NSA (16.1% vs. 3.7%, *p* = 0.005), We observed an increase in the number and levels of c-sIgE sensitization with age, both among non-severe and severe patients. Among school-age children with positive c-sIgE (*n* = 128), four clusters (clusters 4–7) were generated. Cluster 4 (*n* = 4, 3.1%), with “multiple sensitizations, mainly to grass pollens, HDM, PR-10, and nsLTP,”; Cluster 5 (*n* = 6, 4.7%) with “multiple sensitizations, mainly to airborne substances, including grass pollens and HDM”; Cluster 6 (*n* = 24, 18.8%), with “multiple sensitizations, mainly to grass pollens, HDM and PR-10”; Cluster 7 (*n* = 94, 73.4%) with “little sensitization, mainly to HDM”. All four patients in Cluster 4 had AS, compared to 33% in Cluster 5, 25% in Cluster 6, and 34% in Cluster 7 (*p* = 0.036). In school age, sensitization to Gal d 1, hazelnut globulin 2S, canine salivary lipocalin proteins, and nsLTP was more frequent among children with AS and sensitization to nsLTP was associated with impaired LF. Only a small cluster with multiple airborne sensitization and nsLTP was associated with school-age asthma severity (FEV1/FCV Z-score −1.69 [−2.1, −1.4]).	Multisensitization rates were comparable between children with NSA and AS, and no difference in airborne sensitization profiles was found. Sensitization to lipid transfer protein (nsLTP) was more frequent among children with AS than those with NSAAincreased number and levels of c-sIgE sensitization with age, both among non-severe and severe patients. All four patients in Cluster 4 had AS, with significant differences from Cluster 5–6–7. In school age, sensitization to Gal d 1, hazelnut globulin 2S, canine salivary lipocalin proteins, and nsLTP was more frequent among children with AS and sensitization to nsLTP was associated with impaired LF. Only a small cluster with multiple airborne sensitization and nsLTP was associated with school-age asthma severity.	Heterogeneity of population and effect estimates. Incorrect statistical analysis for multiple tests resulting in overestimation of error.	2, 3
De Jong CCM et al. [62]	2020	C, P, M	Evaluate the diagnostic accuracy of symptoms, tests, and diagnostic algorithms by calculating sensitivity, specificity, positive predictive value (VPP), negative predictive value (VPN), and area under the curve (AUC).	Population: children of the Swiss Pediatric Airway Cohort (SPAC), average age 9 years (7–12).	514	Sensitivity, specificity, positive predictive value (VPP), negative predictive value (VPN), Youden Index (sensitivity+specificity–1), area under the curve (AUC), and 95% confidence interval (CI) were calculated for reported symptoms and diagnostic tests to diagnose asthma. Diagnostic accuracy was assessed with the highest Youden index value. Skin tests and specific immunoglobulin E (IgE) assays were used to measure atopy. A wheal size ≥3 mm was considered positive, whereas the positive control (histamine) had a wheal size ≥3 mm and the negative control (saline) had a wheal size <3 mm. Specific IgE levels were considered positive at the detection threshold (≥0.35 kU·L^−1^).	The combined sensitivity and specificity to diagnose asthma were higher for any wheezing (sensitivity = 74%, specificity = 65%), and diagnostic accuracy (AUC) was higher for sRtot (0.74), a positive allergy test (0.70), FEF50% (0.69), FeNO (0.68), and the BDR test in children with FEV1/FVC <80% (0.75).	The combined sensitivity and specificity to diagnose asthma were higher for any wheezing, and the diagnostic accuracy (AUC) was higher for sRtot, at least one positive allergy test, FEF50%, FeNO, and the BDR test in children with FEV1/FVC <80%.	The gold standard for diagnosing asthma, the doctor’s diagnosis, is based on the patient’s medical history and the results of diagnostic tests that are evaluated for accuracy. Some tests were not performed on all children, which could have introduced selection bias, as the children tested could differ from those not tested. However, the percentage of children not tested was low, limiting potential bias as only tests performed on more than 70% of children were evaluated.	3
de Jong CCM et al. [74]	2019	C, P, M	Evaluate the diagnostic accuracy of symptoms and tests by calculating sensitivity, specificity, positive and negative predictive values, and area under the curve (AUC). Evaluate the contribution of a detailed history and various diagnostic tests in the diagnosis of asthma in children.	Children and adolescents with a possible diagnosis of asthma (6–16 years).	111 children enrolled, 80 diagnosed with asthma	At the first visit, all children underwent clinical evaluation, skin tests (or skin test evaluated within the last 2 years), FeNO measurement, spirometry, bronchial stress provocation (BPT) test, BPT with methacholine and reversibility test to the bronchodilator test. For reported respiratory symptoms and different tests, sensitivity, specificity, positive predictive value and negative predictive value, Youden index (sensitivity + specificity −1), area under the curve (AUC) and the related 95% confidence intervals were calculated to diagnose asthma, using the physician’s final diagnosis (post-BPT) as the reference standard.	Wheezing reported within the past 12 months showed the highest sensitivity (80%) for asthma diagnosed by the physician. Specificity was highest for frequent wheezing (more than three attacks per year) (90%), waking due to wheezing (90%), and wheezing triggered by pollen (83%), house dust (93%), or pets (99%). Combined sensitivity and specificity were higher for frequent wheezing over the past 12 months (Youden index 0.34), waking due to wheezing (0.31), and wheezing triggered by pollen (0.29) or pets (0.28). Lower accuracy for skin prick testing (AUC ∼0.70).	Wheezing reported in the past 12 months showed the highest sensitivity for asthma diagnosed by the physician. Specificity was higher for frequent wheezing (more than three attacks per year), waking due to wheezing, and wheezing triggered by pollen, house dust, or pets. Combined sensitivity and specificity were higher for frequent wheezing over the past 12 months, arousal due to wheezing, and wheezing triggered by pollen or pets. Poor accuracy emerged for skin prick tests.	No sIgE level analysis.	3
Lehmann et al. [60]	2017	C, R, S	To investigate the prevalence and impact of Alternaria sensitization on pediatric asthma.	Participants: children with asthma (1–17 years).	207	Data collected included laboratory results [e.g., fluorescence enzyme immunoenzyme (FEIA) values for total immunoglobulin E (tIgE) and allergen-specific immunoglobulin E (sIgE), skin prick test (SPT) results], information on concomitant atopic diseases, family history of atopic, and previous hospital admissions due to asthma exacerbations. Classification of asthma severity was carried out in accordance with the 2002 GINA guidelines regarding documented symptoms and lung function outcomes at the first appointment within the time period mentioned above. Only patients with mild, moderate, or severe persistent asthma were included in the study, while children with intermittent asthma and patients with incomplete data sets were excluded from the study. A total of 207 patients, aged 1 to 17 years, were included in the study and divided into three age groups. A total of 22/207 patients (10.6%) were less than 5 years old and 4 of these (1.9%) were unable to perform spirometry. However, these patients were analyzed and classified, according to GINA 2002, as early childhood asthmatics, since all other study parameters correlated with clear atopy and early childhood asthma. All other patients had a clear diagnosis with diagnostic insights for asthma.	Significant co-sensitization (*p* < 0.01): Alternaria with Penicillium, Cladosporium, Aspergillus, tree pollens, grass pollens; with cat epithelium *p* = 0.04. Hospitalizations and Alternaria: 27% of hospitalized vs. 73% of non-hospitalized (*p* = 0.359, not significant). Total IgE and Alternaria: mean 717 kU/L in sensitized patients vs. 520 kU/L in non-sensitized patients (*p* = 0.282).	- Patients with moderate or severe asthma who are no more frequently sensitized (sIgE) to inhalant allergens than patients with low-grade asthma. - Significant co-sensitization (sIgE) between Alternaria and other molds, - Significant co-sensitization (sIgE) between Alternaria and tree pollen, grass pollen, and cat epithelia. - Alternaria sensitization (sIgE) increases with age (9.1% < 5 years, 15.4% 5–10 years, 24.1% > 10 years).	No mild exacerbations were identified without the need for hospitalization.	3
Castanhinha S et al. [61]	2015	C, P, S	To determine whether fungal sensitization in children with therapy-resistant severe asthma is mediated by IL-33.	Children with severe therapy-resistant asthma (STRA) (6–16 years).	82	Determination of IL-33 levels Overall frequency of diagnoses of severe resistant asthma.	Total IgE levels: SAFS had significantly higher total IgE, with means of 637 IU/mL vs. 177 IU/mL in non-SAFS (*p* = 0.002). Maintenance oral steroid therapy: More frequently prescribed in SAFS (42%) than in non-SAFS (14%) (*p* = 0.02). Airway IL-33 levels: significantly higher in SAFS (no exact numerical measurement is given in the summary, but the qualitative data are clear). Steroid resistance in mouse models: During exposure to A. alternate, even with inhaled budesonide, lung IL-33 levels, ILC2 IL-13^+^ number, TH2 cells, IL-13 levels, and AHR remained elevated.	Pediatric SAFS has been associated with higher oral steroid therapy intake and higher levels of IL-33. Alternative exposure led to an increase in ILC2 numbers mediated by IL-33, TH2 cell numbers and steroid-resistant AHR.	Heterogeneity of the analyzed population Possible heterogeneity of the diagnostic pathway of the included patients.	3
Mingotti et al. [70]	2020	CS, R, M	To assess whether the FEV1/FVC ratio in the lower range of normal is associated with worse outcomes in asthmatic subjects without airway obstruction.	308 subjects aged 6 to 18 years, at eight public and private outpatient pediatric clinics, from August 2017 to July 2019.	308 children	Hospital admissions and frequency of asthmatic symptoms.	The rate of hospital admissions was higher in the lower range of normal FEV1/FVC ratio group than in the other two groups, but the frequency of uncontrolled asthmatic symptoms and the need for moderate-to-high dose inhaled maintenance therapy were similar.	The FEV1/FVC ratio in the lower range of normal is a marker of worse clinical outcomes in asthmatic subjects without airway obstruction.	Heterogeneous definition of clinical outcomes, possible heterogeneity in the strategy underlying the hospital admissions considered.	4
Boonjindasup W et al. [73]	2023	RCT, R, S	To determine whether routine use of spirometry alters clinical decisions and patient-related outcomes in children treated by pediatric pulmonologists.	136 children (aged 4–18) in a specialist center in Australia.	136 children	Change in patients’ clinical management orientation.	The intervention group showed a significantly higher proportion of children with “any change in clinical decisions” (*n* = 54/54 (100%) vs. *n* = 34/52 (65.4%), *p* < 0.001) and a higher “change in clinical decisions” score (median = 2 (IQR 1–4) vs. 1 (0–2), *p* < 0.001).	Routine use of spirometry in children evaluated for respiratory problems during outpatient visits is useful for optimizing clinical management and improving the psychological well-being of parents.	Complex applicability of the concept of clinical guidance, subjectivity of parents in the definition of the concept of quality of life.	4
Yang C L et al. [67]	2017	DC, R, M	Determine the accuracy of asthma diagnosis based on parental report in children selected from a community cohort.	102 asthma cases, 52 controls with respiratory symptoms but no asthma diagnosis, and 49 asymptomatic controls from a community-based sample (age 9–12 years).	203 children	Frequency of symptoms complained of.	The agreement between physicians for the diagnosis of asthma was moderate (kappa 0.46–0.81). Compared to the reference standard, 45% of asthma cases were overdiagnosed and 10% of symptomatic controls were underdiagnosed. The parents’ report on the medical diagnosis of asthma had a sensitivity of 75% and a specificity of 92% in correctly identifying asthma.	There is a significant misclassification of childhood asthma when the diagnosis is based solely on clinical history. This study highlights the importance of objective testing to confirm the diagnosis of asthma.	Possible heterogeneous classification of the included cases.	4
Tran, L.C. et al. [71]	2024	C, P, S	Identify patients at high risk of recurrence of exacerbations using spirometry parameters.	Asthmatic patients aged 6–15 years were studied at the children’s hospital from June 2020 to June 2022 (mean age 9.5 years).	84 children	Frequency of exacerbations.	FEV1, FVC, FEV1/FVC, FEF25–75, FEF25–75/FVC, and PEF gradually decreased with increasing severity of exacerbations (*p* < 0.01). All patients showed a positive bronchodilator response (BDR), with a mean value of 16.85% (3.00), which was significantly different between the severe and non-severe asthma groups (20.53 [2.83] versus 16.00 [2.35], *p* < 0.001).	A high BDR may be a predictor of recurrence of acute asthma exacerbations.	Heterogeneous evaluation of the non-instrumental clinical component.	4
Yimlamai S. et al. [72]	2024	C, R, M	To investigate the prevalence of spirometry abnormalities in a selected population of pediatric asthmatics and their effect on longitudinal outcomes.	203 asthmatic patients (mean age 10.9 ± 2.6 years) who had been receiving asthma treatment for at least 3 months between January 2011 and June 2022.	203 children	Spirometry changes (any).	Impaired spirometry was found in 58.1% of patients under treatment. Altered spirometry results were not associated with worse outcomes during the 12-month follow-up.	Both symptom-based and spirometry-based adjustments from asthma controllers resulted in similar symptom control during a 12-month follow-up in the selected population.	Heterogeneous nature and significance of included outcomes.	4
Barański et al. [175]	2022	CS, R, M	To evaluate the accuracy of FeNO in identifying children with asthma, participants in a population-based respiratory survey.	449 children, 224 (49.9%) males and 225 (50.1%) females aged between 6 and 10 years.	449 children	FeNO alterations.	FeNO was significantly higher in asthmatic children (*n* = 22): 27.3 ± 21.3 ppb; in children with allergic rhinitis (*n* = 106): 9.9 ± 21.6 ppb; in children with atopic dermatitis (*n* = 67): 20.8 ± 25.0 ppb; In children with asthmatic tendency (*n* = 27): 19.8 ± 16.0 ppb compared to children without respiratory or atopic symptoms. The highest diagnostic probability ratio and area under the curve were found in any treated asthma or asthma without atopic symptoms in relation to the FeNO cut-off value > 35 ppb; DOR 4.85 and 8.37; AUC 0.615 and 0.795, respectively.	The best diagnostic accuracy of FeNO was for isolated asthma without atopias compared to children without coexisting respiratory or allergic diseases. Sensitivity and specificity did not reach the values required for an effective screening tool.	Possible non-asthmatic causes of FeNO alteration were not considered Doubtful clinical significance in the absence of accurate comorbidity analysis.	5
Lo DK et al. [81]	2020	C, P, M	To investigate the feasibility, acceptability, training, and ability requirements in carrying out spirometry and FeNO testing in children treated for asthma in UK primary care.	612 children aged 5–16.	612 children	Acceptance and applicability of the use of Spirometry + FeNo.	Spirometry and FeNO are achievable in most children aged 5–16 years after appropriate training of primary care staff.	After training, primary care staff obtained quality spirometry and FeNO data from the majority of children tested. The tests were acceptable to staff and families. Most primary care staff said spirometry helped them better manage asthma in children.	Questionable reproducibility of the concept of applicability.	5
Lo DK et al. [80]	2020	C, P, M	To study the prevalence of impaired spirometry and FENO in children with asthma treated in primary care and explore their relationship to asthma control and unscheduled visits.	612 children aged 5–16.	612 children	FeNO Parameter Alterations in Children Treated in the Primary Care Setting.	23.5% of the children had impaired spirometry, 36.0% had elevated FeNO ≥35 ppb, and 41.8% reported poor symptom control. 54% of children with good asthma control had impaired spirometry and/or elevated FeNO.	Altered lung function and FeNO are common in children who participate in asthma controls in primary care and correlate poorly with symptoms. A symptom-based approach to asthma monitoring without objective testing is likely not to assess children at high risk of future severe asthma attacks.	Doubtful reproducibility (see previous study limits).	5
Chen L. et al. [82]	2023	C, R, M	The association between initial and follow-up measurements of FeNO and disease burden in minority children with persistent asthma.	352 African American and Hispanic children with persistent asthma aged 7 to 20 years.	352 children	Respiratory complications and their association with FeNO variations.	A greater proportion of children with intermediate (54%) and high (58%) levels of FeNO had lower airway obstruction than those with low FeNO levels (33%). Children with intermediate FeNO levels had more annual hospital admissions (2.8 ± 6.2) than those with low and high FeNO levels (1.3 ± 2.8 and 1.3 ± 2.5).	Intermediate and high levels of FeNO are associated with lower airway obstruction and hospital admissions. These associations did not vary between ethnicities.	Children belonging to urban minorities. Incomplete control for covariates. Not assessed the possible different accessibility to care as a reason for the heterogeneity of hospitalization.	5
Smith C. J. et al. [91]	2020	C, P, S	Compare the diagnostic accuracy of upper airway caliper tests (FEV1, FEV1/FVC, R20), peripheral zone properties (X5, VH), and airway inflammation (FeNO) as predictors of poor control in asthmatic children.	54 children with severe asthma (mean age 10.7 years).	54 children	Sensitivity and specificity of respiratory + FeNo functional tests in the associated subclinical state with respect to symptom control.	FEV1% had the highest area under the ROC curve, with low sensitivity but excellent specificity. Among measures of peripheral lung function, X5 and VH in the conductive zone had fair area curves with higher sensitivity but lower specificity than FEV1%. VH in the acinary zone and FeNO both had low accuracy.	Tests of the upper airway and peripheral area showed variable performance as predictors of poor control in a sample of children with severe asthma. Further studies in a larger sample with different phenotypic characteristics are needed to validate this preliminary conclusion.	Doubtful reproducibility Small sample size.	5
Zhu H. et al. [87]	2019	DC, R, M	To investigate the diagnostic value of FeNO combined with small airway function parameters in cough variant asthma.	136 children with asthma were divided into a group with variant cough asthma (CVA) (*n* = 57; mean age 8.03 ± 2.1 years) and a group without variant cough (nCVA) (*n* = 79; mean age 8.61 ± 1.7 years).	136 children	Changes in the FeNO parameter with respect to the evolution of the clinical picture.	FeNO values were significantly higher in the CVA group than in the nCVA group (Z = 6.890, *p* < 0.001). The values of MMEF, MEF25, MEF50, and MEF75 were significantly lower in the CVA group than in the nCVA group (*p* = 0.000, *p* = 0.014, *p* = 0.000, and *p* = 0.000, respectively). FeNO values were negatively correlated with MEF25, MEF50, and MMEF (r = −0.334, r = −0.257, and r = −0.276, respectively).	FeNO combined with lung function parameters of MMEF/MEF50 showed increased sensitivity and specificity for the diagnosis of cough-variant asthma.	Division between asthma variants with and without cough—doubtful reproducibility and heterogeneous interpretation. Relatively small sample size.	5
Dabbaghzadeh, A. et al. [88]	2019	CS, R, S	Compare FeNO, the asthma control test (ACT), and the pulmonary function test (spirometry) in asthmatic children.	76 asthmatic children (ages 8–15 years), who were referred to the Department of Immunology and Allergy (ages 8–15 years).	76 children	Changes in FeNO parameters compared to the clinical picture.	FeNO was significantly correlated with forced expiratory volume (FEV1) (r = 0.232; *p* = 0.049) or maximum expiratory flow at 25–75% (MEF 25–75) (r = −0.304; *p* = 0.009). FeNO did not show a significant correlation with ACT score or FEV1/forced vital capacity (FVC) ratio (*p* > 0.05). In addition, there was no significant correlation between FeNO and changes in FEV1 and MEF 25–75% before and after bronchodilator administration (*p* > 0.05).	To improve asthma control, the ACT, FeNO, and spirometry tests can be used as complementary tools in clinical practice to identify children with poorly controlled asthma.	Patients referred to the Department of Immunology and Allergology in the period 2012–2013, in the context of small overall numbers, leading to doubtful reproducibility.	5
Gao, S. et al. [85]	2018	RCT, R, M	To investigate the correlation between fractionated exhaled nitric oxide (FeNO) levels and the efficacy of inhaled corticosteroids in children with bronchial asthma.	133 cases of children with bronchial asthma were randomly divided into two groups: the glucocorticoid group (*n* = 67) and the non-glucocorticoid group (*n* = 66); and 72 cases were included as a control group.	133 children	Corticosteroid Consumption Versus Change in FeNO.	FeNO levels, percentage of eosinophils in induced phlegm (EOS%), total IgE, and percentage of serum eosinophils (EOS%) were significantly lower, and FEV1/FVC ratio and C-ACT scores were visibly improved in the glucocorticoid group 6 months post-treatment compared to pre-treatment (*p* < 0.05).	FeNO levels could be correlated with the efficacy of inhaled corticosteroids in children with bronchial asthma.	Selected sample from whose doubtful reproducibility of clinical results. Heterogeneous representation of the underlying clinical stage.	5
Yin S.-S. et al. [86]	2017	DC, R, M	To explore the correlation between fractionated exhaled nitric oxide (FeNO) levels and the efficacy of inhaled corticosteroids (ICS) in childhood bronchial asthma.	247 children with bronchial asthma were divided into 3 treatment groups based on drug therapy. 90 patients as a control group.	337 children	Correlation between FeNO, corticosteroids and clinical symptoms.	Compared to the pre-treatment period, 6 months after treatment, FeNO levels, percentage of eosinophils in induced phlegm (EOS%) and inflammatory indices decreased (all with *p* < 0.05), while lung function indices and asthma control test scores in children (C-ACT) increased in the treatment groups.	Inhaled corticosteroids (ICS) are effective in the treatment of bronchial asthma, and FeNO levels associated with the effectiveness of ICS are a useful indicator for intervention in bronchial asthma. The association between FeNO and lung function indices had a predictive value in response to bronchial asthma.	Heterogeneity of the clinical picture found and thus of the monitoring.	5
Eom S et al. [69]	2020	CS, R, S	To investigate the utility of the combination of spirometry indices and fractionated exhaled nitric oxide (FeNO) measured during the initial visit for asthma diagnosis.	Children aged 8 to 16 years, referred for evaluation of suspected asthma.	275 children	Change in respiratory parameters compared to FeNO.	The predicted FEF25–75 percent showed a diagnostic performance with an area under the ROC curve (AUC) of 0.81 (95% CI: 0.76–0.87), which was significantly higher than the predicted percent FEV1 and FEV1/Forced Vital Capacity (FVC) values.	The combined use of predicted FEF25–75 percent and FeNO improved AUC to 0.90 (95% CI: 0.86–0.93). The predicted FEF25–75 percent showed better diagnostic value in detecting childhood asthma than other standard spirometry indices, and its combination with FeNO improves diagnostic accuracy for asthma in children.	Doubtful control for covariates, heterogeneous sample composition. Doubtful duration of follow-up and possible loss of diagnostic detail.	5
Allergic rhinoconjunctivitis										
Cuesta et al. [100]	2019	CS, P, M	To evaluate changes in the quality of life of patients with allergic rhinoconjunctivitis (RA), with or without asthma, after one year of treatment with specific immunotherapy (AIT).	Patients with allergic rhinoconjunctivitis with or without asthma (12–17 years: 22; >18 years: 98).	127	Change in quality of life as measured via the Rhinoconjunctivitis Quality of Life Questionnaire (RQLQ); Patient satisfaction with the treatment; Incidence of adverse reactions.	The mean RQLQ score decreased from 2.61 to 1.34, indicating a statistically and clinically significant improvement (*p* < 0.01). The proportion of asthmatic patients decreased significantly (*p* < 0.01). The mean patient satisfaction score was 7.24 (SD = 1.90). Only 11 patients (9.17%) had systemic reactions, none of which were serious.	One-year AIT treatment significantly increases the quality of life in RA patients. In addition, high patient satisfaction values have been reported, along with an adequate safety profile.	Observational design, which can introduce bias. Lack of a control group. Potential variability in treatment protocols between centers.	1
Matsumoto et al. [93]	2024	CS, R, M	Identify and characterize local allergic rhinitis (LAR) in children and adolescents previously diagnosed with non-allergic rhinitis (NAR) in a specialized clinic.	Patients diagnosed with rhinitis who tested negative on skin test for allergens (6–18 years).	758	Diagnosis of LAR: identification of patients with a previous diagnosis of NAR and positive on NAC test; Clinical features: review of symptoms and clinical features of patients with LAR; Safety of NAC: Evaluation of the Safety of NAC in Pediatric Patients.	95 (18.4%) had negative skin test results and were included in the study; 25 patients underwent NAC; of these, 10 (40%) showed a positive response.	Carried out a new classification from NAR to LAR; It was not possible to clinically differentiate LAR from NAR based only on clinical features. Multi-allergen NAC has been found to be safe and feasible in the pediatric population.	Relatively small sample (25 patients undergoing NAC); study conducted in a single specialty clinic, limiting the generalizability of results; lack of long-term follow-up to assess the persistence of LAR.	1
Smith et al. [91]	2016	DC, P, M	To determine whether structured allergy assessment and personalized counseling in general practice improve outcomes in children aged 6–16 years with asthma and/or rhinitis.	Children with asthma and/or rhinitis (6–16 years).	335	Symptom scores and disease-related quality of life (QoL); utilization of health care, days when normal activities cannot be performed, and self-assessed improvement.	(mean difference −3.14, 95% CI −6.01 to −0.81) and improvement in quality of life (mean difference −0.50, 95% CI 0.32 to 0.68) compared to the usual care group.	The allergy intervention group showed significantly fewer symptoms of rhinitis; No significant changes were observed in asthma symptoms, health care utilization, or days when normal activities could not be performed.	The study found no significant changes in asthma symptoms or health care utilization, indicating that the intervention may be more effective for rhinitis symptoms. The study was conducted in a general practice setting, which may limit the generalizability of the findings to other healthcare settings.	1
Cicek et al. [92]	2023	CS, R, S	Investigate the role of hematological parameters and systemic inflammatory biomarkers in the diagnosis of allergic conjunctivitis (AC) in children.	Children diagnosed with allergic conjunctivitis and a similar control group by sex and age (11.3 ± 3.7 years for patients and 10.9 ± 3.9 for controls).	142	Comparison of hematological parameters; diagnosis of AC.	Eosinophils and total IgE: both significantly higher in patients with AC than in controls (*p* < 0.001); Distribution of AC types: 70.4% with seasonal allergic conjunctivitis (SAC), 29.6% with perennial allergic conjunctivitis (PAC); Sensitivity to allergens; Each 100-cell increase in eosinophil count increased the risk of AC by 1.3-fold; Each 100-unit increase in total IgE levels increased the risk of AC by 1.2-fold.	Although CA is a clinical diagnosis, laboratory tests support the diagnosis. Cytological examination, skin prick tests, and detection of total IgE antibodies in serum are important in the diagnosis of CA.	Retrospective and single-center study; limited number of patients; lack of long-term follow-up; need for multicenter and prospective studies to confirm results.	1
Garrido Fernandez et al. [99]	2024	CS, R, M	To evaluate the impact of sublingual immunotherapy (SLIT) on symptom control, quality of life, patient satisfaction, and adherence to therapy in individuals with allergic rhinitis and/or house dust mite-induced asthma and/or pollen.	Patients (approximately 50% of whom are children and adolescents) suffering from allergic rhinitis and/or house dust mite-induced asthma and/or pollen, who have undergone SLIT for at least 6 months or a pre-season for pollen (mean age 24.9 SD 17.6).	859	Control of symptoms (nasal, ocular and bronchial); use of medications; health-related quality of life (HRQoL); Patient satisfaction; adherence to SLIT.	Improvement in global health-related quality of life (HRQoL) scores, both in the overall sample (+44.2%) and in the subgroup of patients with asthma, as reflected by the assessment of patients (+31.0%, *p* ≤ 0.005).	Significant improvement in the control of symptoms of allergic rhinoconjunctivitis and asthma, resulting in reduced medication use; significant improvement in health-related quality of life (HRQoL), with improvements in nasal, ocular, and bronchial symptoms and daily activities.	Retrospective design, which could introduce bias; Potential recall bias in patient-reported data; lack of long-term follow-up data; Single-arm study without control group.	1
Katsimpris et al. [98]	2022	CS, P, S	To develop and validate the Greek versions of the Quality of Life Questionnaire in Rhinoconjunctivitis (RQLQ) and its modified short version (Mini-RQLQ) for both adult and pediatric populations, and to assess the impact of age and gender on quality of life outcomes.	Patients with allergic rhinitis (9–79 years).	98	Internal consistency and test–retest reliability of RQLQ and Mini-RQLQ; convergent validity with Short Form-36 (SF-36) and Beck Depression Inventory; discriminative validity in the evaluation of changes after treatment; impact of age and gender on quality of life scores.	Cronbach’s alpha coefficient was 0.92 for the Rhinoconjunctivitis Quality of Life Questionnaire as a whole.	The Greek versions of the Rhinoconjunctivitis Quality of Life Questionnaire and its short version are reliable and valid methods to explore well-being in relation to allergic rhinitis, with high specificity and sensitivity for both the pediatric population and adolescents and adults.	Single-center study, which limits generalizability; potential cultural differences in questionnaire responses; lack of long-term follow-up data	1
Proctor et al. [101]	2020	CS, R, S	To evaluate and compare the impact on quality of life (QoL) and safety of three types of specific immunotherapy in children with severe allergic rhinoconjunctivitis due to house dust mites (HDM) or pollen: subcutaneous anti-pollen immunotherapy (SCIT), sublingual anti-pollen immunotherapy (SLIT) HDM SLIT.	Children (4–17 years) with severe rhinoconjunctivitis.	249	Change in Rhinoconjunctivitis Quality of Life Questionnaire (RQLQ) scores; Visual Analog Scale (VAS) symptom scores at 1 and 3 years. Safety outcomes: incidence of anaphylaxis and asthma exacerbations after immunotherapy; treatment discontinuation rates.	QLQ improved by > 50% from baseline in all groups. A significant improvement in quality of life was evident by the end of the first year of therapy (41–47% for RQLQ; 29–32% for VAS). A further improvement in RQLQ scores was observed between the first and third years (36–72%).	All therapies showed a 50% improvement in RQLQ over > 3 years of treatment, and the efficacy of no therapy was significantly better than any other.	Retrospective design: subject to selection and reporting bias; single-center study: limits generalizability; no placebo or untreated control group.	1
Martinez et al. [118]	2021	C, P, M	To compare the efficacy, safety, and immunological effects of pre-co-season versus perennial programs of a depigmented-polymerized grass and olive (Olea europaea) SCIT therapy in children with allergic rhinitis, with or without controlled asthma.	Children and adolescents (5–16 years) with moderate or severe rhinoconjunctivitis.	40	Evaluation of the Quality of Life Questionnaire in Pediatric/Adolescent Rhinoconjunctivitis (PRQLQ/AdolRQLQ) During Pollen Season With and Without Immunotherapy.	Changes in PRQLQ/AdolRQLQ scores showed a significant improvement of 1.70 in the group with the perennial program (*p* < 0.0001) and 1.91.	An improvement in quality of life was observed after the start of treatment during the pollen season.	Small sample size (*n* = 40), which limits statistical power and the ability to detect small intergroup differences; uneven distribution between groups (29 vs. 11), which could influence comparisons; seasonal comparison design rather than parallel RCT structure: potential seasonal confounders; short-term concentration on a post-treatment pollen season, without long-term follow-up.	1
Nolte et al. [117]	2016	RCT	To evaluate the efficacy and safety of a 12SQ-HDM sublingual immunotherapy tablet compared to placebo in North American patients (aged ≥12 years) with house dust mite-induced allergic rhinitis/conjunctivitis, with or without asthma.	Patients (Patients 12–77; Placebo 12–85) with allergic oculorinitis with more or less severe symptoms.	1482	Assessment of Combined Mean Total Rhinitis Score (DSS + DMS) During the Last 8 Weeks of Treatment, Daily Symptom Score (DSS), Medication Score (AMD), Combined Total Rhinoconjunctivitis Score (TCRS), Visual Analogue Scale (VAS) for Allergic Symptoms.	TCRS: 17% improvement compared to placebo (95% CI: 10–25%; *p* < 0.001), DSS, VAS, and overall TCRS improved by approximately 16–18%, all statistically significant (*p* < 0.001), except for the drug score, which showed a trend (*p* = 0.15).	The efficacy of the drug related to dust mites and its safety in use were highlighted.	Duration limited to about 1 year; prolonged post-treatment efficacy remains undefined; Asthma status not explored in detail; includes patients with and without asthma, but without subgroup analysis.	1
Okamoto et al. [116]	2017	RCT	To evaluate the efficacy and safety of two doses (300IR and 500IR) of HDM-SLIT tablets compared to placebo in adolescents and adults (12–64 years) with HDM-induced allergic rhinitis, with or without intermittent asthma.	Children and adults (12–64 years) with rhinitis allergic to dust mites.	968	Assessment of the adjusted mean symptom score (AASS) during the last 8 weeks of treatment; individual nasal/eye symptom scores, rescue medication use, and quality of life via the Japanese RQLQ.	The difference between the 300 IR and 500 IR groups was not statistically significant. Compared to placebo, AASS in the 300 IR group improved significantly at week 8–10 (*p* = 0.0012); it was also observed in the 500 IR group at week 8–10 (*p* = 0.0448).	The use of the sublingual drug led to an overall improvement in the symptoms of allergic rhinitis from mites.	Study duration of 1 year; therefore, long-term efficacy or sustained post-treatment benefits are unknown; subgroup of patients with asthma not defined in the results; efficacy in intermittent co-treatment of asthma remains exploratory; comparison of doses, but no subgroup analysis (e.g., adolescents vs. adults) was detailed.	1
Soh et al. [115]	2016	CS, P, S	To evaluate the clinical efficacy and safety of SLIT against house dust mites (HDM), including the allergen *Blomia tropicalis*, in children and adults with allergic rhinitis from HDM over a two-year period.	Adult and child patients (7–18 years) with dust mite allergy and positive SPT for D.Pteronyssinus, D Farinae and B Tropicalis.	24	Assessment of Nasal Symptom Total Score (TNSS), Mini Rhinoconjunctivitis Quality of Life Questionnaire (Mini RQLQ), and Medication Use Score.	With a baseline score of 8.38, there was a significant reduction in scores at all time points, with a lower score of 4.80 at two years (*p* = 0.02).	Sublingual immunotherapy is effective in providing symptomatic relief and improving quality of life in patients with allergic rhinitis from house dust mites.	Small sample size; lack of a control group; limited data security.	1
Duan et al. [114]	2016	C, R, S	To evaluate and compare the clinical efficacy and safety of sublingual immunotherapy (SLIT) and subcutaneous immunotherapy (SCIT) in children with house dust mite-induced allergic rhinitis, over a 2-year treatment period.	Children with dust mite-induced allergic rhinitis.	186	Evaluation of the clinical score through validated questionnaires, inflammatory markers and safety.	TRSS, VAS, RQLQ, SI and ECP (all *p* < 0.05) after 2 years; No significant change in sIgE levels in either group (*p* > 0.05); Safety: Adverse reaction rates were low and similar: 3.85% for SLIT versus 2.44% for SCIT; No serious systemic events have been reported.	Immunotherapy has an effect on the reduction in the clinical score and absolute safety in its use.	“Non-random assignment; No placebo control arm; Lack of detailed layering.	1
Lou et al. [113]	2018	CS, P, S	To evaluate the long-term (5-year) efficacy and safety of a 3-year standardized HDM-SCIT regimen in pediatric and adult patients with allergic rhinitis.	Children and adults (5–51 years) with dust mite allergy or house dust without asthma.	48	Clinical evaluations at the beginning, at 3 years and at 5 years; safety assessed by adverse event monitoring.	3-year efficacy (TNSS 6.75 to 5.09; TCS from 4.63 to 3.66) at 5 years (TNSS 6.63 to 4.38; TCS 4.56 to 3.34).	Both children and adults showed significant improvement in TNSS, TCS, and RQLQ after 3 years, which was also maintained in the 5-year assessment; Pediatric patients demonstrated greater long-term efficacy than adults at both 3 and 5 years; The safety profile was comparable across age groups, with no long-term emerging risks reported.		1
Lin et al. [114]	2016	RCT	To evaluate whether the clinical efficacy of SLIT on *Dermatophagoides farinae* (HDM) in allergic rhinitis improves with longer treatment durations (1, 2, or 3 years) and to evaluate whether the benefits persist after discontinuation.	Children and adults (4–69 years) with a clinical history of RA 1.5 years; allergic symptoms and signs such as runny nose, itchy nose, sneezing, nasal crease, pale and swollen nasal mucous membranes, and watery discharge; positive clinical history of allergy to HDM; and positive skin test (SPT) for *Dermatophagoides farinae*.	500	Evaluation of efficacy, safety and use of drugs with the use of specific immunotherapy.	All SLIT groups showed significant improvement from baseline in TNSS, VAS, and RQLQ scores (*p* < 0.05). The SLIT group had the highest drug discontinuation rate at 3 years (*p* < 0.05), indicating superior clinical benefit.	The efficacy, safety and decrease of drugs in the acute phase have been confirmed thanks to the use of immunotherapy.	Lack of long-term follow-up beyond 2 years”.	1
Schuster et al. [111]	2025	RCT	To evaluate the efficacy and safety of daily sublingual immunotherapy 12SQ-HDM in children aged 5–11 years with house dust mite-induced allergic rhinitis/rhinoconjunctivitis, with or without asthma, for approximately 1 year of treatment.	Children (5–11 years) with a positive diagnostic test; FEV1 of at least 70%; DSS ≥ 6; were taking medication for more than 8 of the 14 days.	1460	To evaluate the efficacy and safety of daily sublingual immunotherapy 12SQ-HDM in children aged 5–11 years with house dust mite-induced allergic rhinitis/rhinoconjunctivitis, with or without asthma, for approximately 1 year of treatment.	The mean daily TCRS for SQ HDM SLIT tablet compared to placebo was 1.0 (95% CI: 0.5, 1.4; *p* < 0.0001), which corresponds to a relative reduction of 22.0% (95% CI: 12.0, 31.1); higher rate of treatment-related adverse events, mostly mild to moderate (mainly oral/local); only 2.5% discontinued treatment due to adverse events.	Demonstration of the Efficacy and Safety of Long-Term Immunotherapy for Dust Mite Allergy.	Small sample size; no randomized or double-blind procedures, limited demographics and baseline data available, lack of immunological biomarkers that could elucidate the mechanism of action.	1
Catamerò et al. [110]	2025	CS, P, S	To evaluate the impact of sublingual immunotherapy (SLIT) tablets against house dust mites (HDM) on quality of life (QoL) in Italian adolescents suffering from HDM-induced allergic rhinitis.	Italian adolescents (13–14 years) who have been taking immunotherapy for 52 weeks.	15	Assessment of quality of life measured through validated questionnaires (e.g., Rhinoconjunctivitis Quality of Life Questionnaire, RQLQ); symptom scores and medication use; Safety and Tolerability of SQ HDM SLIT Tablets.	The median values of RQLQ(s) at T2 (0.8570, IQR = [0.2855, 1.240]) and T1 (1.1070, IQR = [0.5445, 1.5172]) were significantly lower than the median value at T0 (2.042, IQR = [1.268, 2.893]).	Improved Quality of Life in Adolescents Who Used Immunotherapy.	Absence of a placebo group; self-reported results, limited follow-up; do not report the size of the subgroups.	1
Yuta et al. [109]	2020	CS, P, S	To evaluate the efficacy and safety of pediatric sublingual immunotherapy (SLIT) with Cedarcure^®^ in children with Japanese cedar pollinosis during the first pollen season following initiation of treatment.	Children and adults (6–66 years) with cedar allergy and a history of bowel disease.	491	Evaluation of clinical efficacy, drug use and safety of sublingual therapy.	Blood sIgE differences (Children 46.2 ± 40.4 AU/mL; adults 11.1 ± 23.2 AU/mL); reduction of cica 40–50% of all allergic symptoms according to the VAS and JRQLQ scale.	Improvement of symptoms, better allergic profiles, and validated safety.	Conducted at specific sites; not specific for asthma-related, possible conflict of interest.	1
Caffarelli et al. [108]	2000	RCT	To evaluate the efficacy and safety of pre-season local immunotherapy with allergoids in children with grass pollen allergy.	Children (4–14 years) with pollen allergy.	48	Symptom control during pollen season, medication use during pollen season, and adverse events.	Total symptom scores during the pollen season (as a weekly average) were lower in the treated group than in the placebo group; the difference was statistically significant (mean [SD] 9.5 [7.2] vs. 14.5 [8.3]; *p* < 0.05); the symptom-drug score in the immunotherapy group was lower than in the placebo group during the pollen season in all weeks. This difference was statistically significant at week 3 (*p* < 0.05), week 7 (*p* < 0.05), and week 8 (*p* < 0.03).	A decrease in symptoms during the pollen season and in the use of acute drugs.	Observational study design without a control group; moderately sized sample; the results may not be generalizable outside the Italian adolescent population.	1
Horn et al. [107]	2016	CS, P, M	To evaluate changes in health-related quality of life (HRQoL) in patients with grass pollen allergy during routine treatment with the SQ standardized grass allergy immunotherapy tablet.	Patients (12–68 years old) with pollen allergy and candidates for the use of GRAX2.	576	Health-related quality of life assessed via the Quality of Life in Rhinoconjunctivitis (RQLQ) questionnaire; Safety and Tolerability of Standardized Grass Allergy Immunotherapy Tablet With SQ During Routine Use.	Overall, the data were analyzed on 576 patients. The mean (±SD) differences in overall scores for HRQoL3 versus HRQoL1 (186 patients) for SF-12 were ≥11.4 ± 16.8 (slit tablet) and −3.4 ± 15.7 (symptomatic drug), (*p* < 0.0001), and for RQLQ −1.31 ± 1.07 and ≥0.10 ± 0.74 (*p* < 0.001), and for HRQoL3 versus HRQoL2 (238 patients) of SF-12 −1.6 ± 15.3 and −10.0 ± 14.1 (*p* = 0.0003), and RQLQ ≥0.22 ± 1.29 and ≥1.24 ± 1.30 (*p* < 0.0001).	Routine treatment with SQ herb SLIT tablet resulted in clear improvements in disease-specific and overall health-related quality of life (HRQoL) compared to non-specific treatment with symptomatic therapy alone.	Small sample size; short follow-up.	1
Ren et al. [106]	2023	CS, P, S	To evaluate and compare the long-term efficacy (up to three years of treatment followed by more than three years of post-treatment follow-up) of cluster-based HDM subcutaneous immunotherapy (SCIT) in children versus adults with perennial allergic rhinitis.	Patients (5–60 years) with rhinitis allargica due to dust mites.	161	Assessment of Total Nasal Symptom Score (TNSS); Combined Symptom and Medication Score (CSMS); Quality of Life Questionnaire in Rhinoconjunctivitis (RQLQ); Sustained efficacy after 3 years of treatment and follow-up of more than 3 years.	(MED [IQR]; TNSS, 4.00 [1.00, 8.00] vs. 3.00 [−2.00, 6.00], *p* = 0.005; TOSS, 1.00 [0.00, 3.00] vs. 1.00 [−1.00, 2.00]; CSMS, 1.50 [0.50, 2.67] vs. 1.00 [−0.21, 2.17], *p* = 0.024).	SCIT is an effective and well-validated alternative for patients with HDM-induced RA, but it is less practical than SLIT; children and adults with HDM-induced perennial RA have achieved sustainable efficacy of a three-year SCIT for at least 3 years (up to 13 years) post-treatment.	Small sample size; study focused only on pre-season treatment; limitedly generalizable; not long follow-up.	1
Liu et al. [105]	2018	CS, P, M	Measure specific salivary immunoglobulins (DpIgA, DpIgE, DpIgG4) in allergic rhinitis (RA) patients undergoing subcutaneous immunotherapy (SCIT), during the dosage escalation and maintenance phases, compared to untreated RA patients and healthy controls, and correlate them with clinical symptoms and quality of life scores.	Patients positive only for Dp, as determined by a skin prick test, with serum anti-Dp IgE levels >0.70 kU/L (4–70 years).	82	Study of IgA, IgE and IgG4 specific levels for Dp in saliva (quantified by ELISA); clinical outcomes: symptom scores, medication use, and Rhinoconjunctivitis Quality of Life Questionnaire (RQLQ) scores; correlation between salivary immunoglobulin levels and clinical endpoints.	IgG4 levels were significantly lower in RA patients who had not undergone SCIT (*p* < 0.001 for both), while salivary IgE levels were significantly higher (*p* < 0.001). Dp-IgA, Dp-IgE, and Dp-IgG4 levels were significantly higher in dose-escalation patients compared to patients not treated with SCIT (*p* < 0.001, P 1/4 0.004, *p* < 0.001).	Allergen-specific IgA and IgG4 in saliva may protect against allergic disease and may serve as objective indicators for assessing response to SCIT treatment.	Non-interventional design; potential selection bias as patients were treated in routine clinical settings; health-related quality of life assessed only through patient-reported outcomes; lack of long-term follow-up beyond the treatment period.	1
Macedo et al. [104]	2025	RCT	To evaluate the efficacy and safety of a combined SLIT *Dermatophagoides pteronyssinus* + *Blomia tropicalis* in adolescents and young adults (12–16 years) with allergic rhinitis sensitized to both mites.	Patients (12–60 years) with moderate/severe persistent allergic rhinitis, rhinitis symptoms for 2 years or more, and sensitized to D. pteronyssinus and B. tropicalis, defined by positive skin tests (3 mm) and/or presence of serum immunoglobulin E (IgE) specific for D. pteronyssinus and B. tropicalis as measured by ImmunoCAP (>0.35 kU/L).	118	Analysis of Rhinoconjunctivitis Quality of Life Questionnaire (RQLQ) scores, medication use (antihistamines, nasal steroids, etc.), and immunological markers (total IgE, specific IgE, IgG4 for Der p 1, and Blo t) at baseline, 6, and 12 months; Total Nasal Symptom Score (TNSS).	Significant reduction in antihistamine use (loratadine) in the SLIT group (~78% to ~16%) compared to placebo (~82% to ~73%) at 12 months (*p* < 0.0001); There was no significant difference in serum immunoglobulin levels between groups at baseline (*p* > 0.05, Chi-square test).	After 1 year, SLIT drops containing 1 mcg of Der p 1/day and 753 UBE of Blo t/day promoted a reduction in oral antihistamine intake, indicating better control of rhinitis exacerbations for the placebo group.	No placebo control group present; high drop-out rate; single-center; based on SCIT protocols.	1
Agenas et al. [103]	2023	CS, P, S	To evaluate the long-term effect of subcutaneous pollen immunotherapy (SCIT) on health-related quality of life (HRQoL) and symptom burden in children and adolescents with allergic rhinoconjunctivitis over a 3-year period.	Children (5–16 years) with rhinoconjunctivitis and asthma.	246	Analysis of health-related quality of life measured using the 37-item DISABKIDS questionnaire (scale from 0 to 100); Nasal, ocular, and pulmonary symptom score (combined score from 0 to 36); Serum levels of allergen-specific IgE and IgG4 (birch and/or grass pollen).	Improvement in quality of life: The mean DISABKIDS score increased from 79.5 at baseline to 85.1 after 1 year (*p* < 0.001), stabilizing at 84.8 (2 years) and 87.2 (3 years); Symptom reduction: The mean symptom score decreased from 19.9 to 11.5 after 1 year (*p* < 0.001), remaining low at 11.9 (2 years) and 10.3 (3 years); Prevalence of severe symptoms: drastically reduced from 35.6% at baseline to 4.5% after 1 year.; Immunological changes: allergen-specific IgE (birch) decreased from 151.0 to 76.8 kU/L; Increased IgG4 (birch: 2.2 → 17.6 g/L; grass: 0.5 → 14.3 g/L); Association: Higher symptom scores correlate with lower health-related quality of life (HRQoL) throughout the study (*p* = 0.001–0.031).	Immunotherapy has an effect on increasing the quality of life, modifying the therapy in progress and causing immunological changes.	Study not fully longitudinal for the individual, salivary biomarkers only, lack of clinical correlation, monosensitized population, moderate sample size.	1
Kiotseridis et al. [176]	2018	CS, P, M	To evaluate the adherence, quality of life (QoL), safety, and tolerability of 3 years of GRAZAX^®^ (SSLIT grass pollen tablet) in children (≥5 years) and adults under routine care conditions.	Children and adults (5–67 years) with rhinoconjunctivitis symptoms.	399	Analysis of the 3-year adherence rate (measured by patient-reported tablet intake); completion rates (completed vs. interrupted vs. lost at follow-up); symptom score via VAS during peak pollen season; health-related quality of life assessed via RQLQ (adults) and PADQLQ (children).	Completion rate: 220/399 (55%) completed the three-year treatment; children showed a higher completion rate (69%) than adults (45%). Adherence levels: 98% first month; 93.7% year-end 1; 93.2% year-end 2; 88.9% year-end 3 (group by protocol); Symptom improvement: mean VAS decreased from ~7.7 at baseline to ~2.7 after 3 years (*p* < 0.01).	Adherence and quality of life with the use of immunotherapy are very high.	Single-center; limited sample size; doses and immunological markers could be too low to create a response; short duration of the study.	1
Yang et al. [102]	2018	CS, P, S	To evaluate the impact of sublingual immunotherapy (SLIT) on quality of life (QoL) in children diagnosed with allergic rhinitis.	Children (6–12 years) diagnosed with allergic rhinoconjunctivitis.	50	Evaluation of efficacy, increased quality of life and response to treatment.	20 children in complete remission, 13 with partial remission; VAS scores showed significant improvement at 0.5, 1, and 2 years from baseline (*p* < 0.05) RQLQ scores: significant improvements (*p* < 0.05) in all domains of quality of life (nasal symptoms, ocular symptoms, non-nasal symptoms, emotional status, behavior) at all follow-up points (0.5, 1, and 2 years).	Patients have partly gone into remission and, in general, have found benefit with the use of immunotherapy.	“No control/placebo group due to ethical constraints, which limit causal conclusions; subjectivity in quality of life assessments and potential influence of parental care in younger children; symptom scoring tool not formally validated.	1
Paoletti et al. [96]	2025	CS, P, S	To evaluate usage patterns, patient awareness and the impact of specific immunotherapy (AIT) in Italy, focusing on respiratory allergies and the role of patient associations in promoting AIT.	Patients (35 ± 16.8 years old) affiliated with the patient association Respiriamo Insieme APS, interviewed between May and October 2023.	944	Know patients’ awareness of AIT; percentage of patients who were offered AIT; reasons for refusal or termination of the IWA; perceived efficacy and safety of AIT; impact of AIT on school/work absenteeism.	81.1% of patients reported allergic rhinitis, 45.4% allergic asthma and 41.2% allergic conjunctivitis; many had a combination of these conditions; 53.8% were aware of the AIT; 33.1% of patients with allergic rhinitis and/or asthma were offered AIT; 29.2% of those offered the IWA refused, citing indecision (26.5%), costs (22.9%) and skepticism (19.3%); 70.8% accepted the IWA; 21.4% discontinued treatment after an average of 18.26 months; 20.4% were advised to continue AIT for less than 3 years.	Many eligible patients chose not to start AIT, and many patients discontinued treatment early. A high degree of heterogeneity in prescribing patterns was also observed.	Lack of data on concomitant medication use, which could affect symptoms and quality of life outcomes”.	1
Nyirazeva et al. [95]	2020	CS, R, S	Describe the impact of allergic rhinitis on quality of life (QoL) in a cohort of Rwandan children and adolescents.	Children (6–17 years) diagnosed through the Score for AllergicRhinitis (SFAR).	115	Assess the impact of symptoms on quality of life: identify the most distressing symptoms for children and adolescents, assess the intensity of the impact on quality of life, and the differences between age groups.	Limitation to physical activity 2.70; difficulty sleeping 3.25, difficulty in attention 2.45; serous rhinitis in 3.57 according to the AdolRQLQ and PRQLQ scales.	Allergic rhinitis has a major impact on the everyday activities of positive subjects.	No control group present, adherence is reported by the patient with subjective data, variability between clinics and non-characterization of the reasons for discontinuation.	1
Klangkalya et al. [94]	2024	CS, R, S	Establish limit values for the mean wheal diameter (MWD) of the skin test (SPT) for house dust mites (HDM) and HDM-specific immunoglobulin E (sIgE) levels to identify children with HDM-induced allergic rhinitis (RA) diagnosed via nasal provocation test (NPT).	All children (5–18 years) with chronic rhinitis who underwent evaluation for SPT, sIgE for HDM and HDM NPT at the pediatric allergy clinic of Ramathibodi Hospital from 2017 to 2020 were included in the current retrospective study.	245	To evaluate the diagnostic accuracy of HDM SPT and sIgE levels in predicting positive NPT responses.	Positive HDM SPT in 160 children (65.3%); HDM positive NPT in 176 children (71.8%). Among the 160 children with HDM-positive SPT, 153 (95.6%) confirmed RA via NPT; threshold values: SPT MWD ≥6.6 mm: 100% specificity, 100% positive predictive value; sIgE ≥17.0 kUA/L: 98.6% specificity, 99.2% positive predictive value.	Established limit values for the HDM SPT MWD test and for the sIgE level to predict HDM-induced RA according to the results of the HDM NPT for children with chronic rhinitis.	Single center; moderate sample, limited follow-up.	1
Alimuddin et al. [177]	2018	CS, R, S	To compare the diagnostic accuracy of serum-specific IgE assay using the Immunoblot method (Euroline^®^) with the skin test (SPT) to detect sensitization to house dust mites (*Dermatophagoides pteronyssinus*, *Dermatophagoides farinae*, *Blomia tropicalis*) and cockroach (Blatella germanica) in patients with asthma and/or allergic rhinitis.	Patients (19–59 years old) diagnosed with asthma and/or allergic rhinitis who have signed informed consent, agreed to undergo skin testing, and have not taken antihistamines and corticosteroids within 7 days.	101	Establish diagnostic performance parameters, including sensitivity, specificity, positive predictive value (VPP), negative predictive value (VPN), positive likelihood ratio (LR+), and negative likelihood ratio (LR−).	With regard to PTS, the highest sensitization rates were observed for *Blomia tropicalis* (76.2%), followed by *Dermatophagoides pteronyssinus* (70.3%), *Dermatophagoides farinae* (69.3%) and Blatella germanica (41.6%); with regard to serum-specific IgE testing, the highest sensitization rates were observed for *Dermatophagoides farinae* (52.9%), followed by *Dermatophagoides pteronyssinus* (38.2%), *Blomia tropicalis* (33.3%) and Blatella germanica (10.8%).	Serum-specific IgE testing can replace skin testing to determine allergen sensitization in a population with high respiratory allergy (asthma and/or allergic rhinitis), individuals with dermographism, individuals with generalized skin disorders, and patients requiring continuous antihistamine treatment or treatment that affects the diagnostic test.	Self-reported data can introduce bias; no assessment of long-term clinical outcomes; lack of data on other potential allergens.	1
Alessandri et al. [55]	2018	CS, R, M	Investigate the different IgE sensitization profiles in six countries (Croatia, Hong Kong, Iran, Italy, Poland, Romania) using the FABER test, a multiplex diagnostic tool with 244 allergens.	Atopic patients	1186	To investigate the prevalence of IgE sensitization to specific food and inhalant allergens in different countries.	Homogeneous sensitization to dust mite allergens in all countries; significant differences in sensitization to specific allergens, such as Der p 2 (66% in Hong Kong vs. 2% in Romania) and Der p 23 (46% in Hong Kong vs. 5% in Romania); changes in sensitization to pollen allergens, with Bet v 1 being more prevalent in PL (33%) and lower in IR (3%); higher prevalence of lipid transfer protein (LTP) sensitization in IT (19%) and PL (11%); Differences in sensitization to animal allergens such as Can f 1 and Fel d 1, with PL patients showing a higher prevalence (20% and 42%, respectively) than IR patients (2% and 13%, respectively).	The FABER test allows you to detect the prevalence of sensitization to inhalant and food allergens using routine diagnosis data.	Cross-sectional study that does not allow to establish a causal link; sample limited to a single hospital in Kigali, reducing the generalizability of the results; lack of a healthy control group to compare data.	1
Masrur et al. [129]	2020	CS, R, S	To explore the relationship between the severity of allergic conjunctivitis, assessed using the “5–5–5” severity scale, and skin test results in patients diagnosed with allergic conjunctivitis.	Subjects (10–60 years old) diagnosed with allergic rhinoconjunctivitis who have already undergone SPT.	150	To evaluate the relationship between skin test results and the severity of allergic conjunctivitis, and analyze the distribution of skin test results in relation to the severity of the condition.	Based on the 5–5–5 rating scale, a total of 18.7% (*n* = 28) of cases had mild allergic conjunctivitis, 51.3% (*n* = 77) had moderate conjunctivitis, and 30% (*n* = 45) had severe conjunctivitis. The average score of these categories, based on the result of the skin test, is shown in Table 2. There was no statistically significant difference in the mean score of patients with mild (*p* = 0.44), moderate (*p* = 0.89), or severe (*p* = 0.73) allergic conjunctivitis with either a positive or negative skin test.	In each of the three categories of allergic conjunctivitis (mild, moderate, and severe), the mean score on the 5–5–5 rating scale was greater in patients with a positive skin prick test than in those with a negative prick test.	Single-center study, potentially limiting generalizability; lack of long-term follow-up data to assess clinical outcomes; no evaluation for other potential allergens.	2
Arasi et al. [160]	2021	CS, R, M	To develop and validate a novel algorithm to guide the prescription of specific immunotherapy (AIT) in patients with seasonal allergic rhinitis, with the aim of improving patient selection and treatment outcomes.	Participants were recruited from among physicians who regularly collaborate with each center.	76	Accuracy and effectiveness of the algorithm in guiding the prescription of AIT; clinical characteristics of patients selected by the algorithm; improved therapeutic decision-making.	Based on the available choices (*n* = 110 and *n* = 70 respectively for Rome and Pordenone), only 54% (Rome) and 59% (Pordenone) of the AIT prescribed by AS at the first step of the CDSS corresponded to the gold standard choice of AIT. These percentages increased to 66% (Rome) and 83% (Pordenone) in the second step, and further to 86% (Rome) and 87% (Pordenone) in the third step.	The algorithm effectively stratified patients, improving the accuracy of AIT prescribing; It helped identify candidates most likely to benefit from AIT.	The sample size; the study did not evaluate the clinical relevance of the sensitizations detected.	2
Douladiris et al. [130]	2019	CS, R, M	To evaluate the efficacy of a single recombinant hybrid containing the four major phleol pollen allergens in the skin assay for in vivo diagnosis of genuine grass pollen sensitization in children with sheep fever.	Children (6–17 years) with symptoms of sheep fever and sensitivization to pollen.	64	To evaluate genuine sensitization to grass pollen using the recombinant hybrid in the skin test; compare skin test results with those obtained from individual recombinant allergens and grass pollen extracts; analyze the allergen-specific IgE reactivity of grass pollen using ISAC ImmunoCAP technology.	94% of children with positive grass pollen skin tests showed true grass pollen sensitization by recombinant hybrid skin tests and hybrid IgE reactivity; Sixty-one of the 64 children developed a positive skin reaction to the herbal mix (mean wheal diameter 6.2 mm ± 2.6) and 57 of the 64 children had a positive SPT to the fleolus pollen extract (mean wheal diameter 5.2 mm ± 2.2).	The result of the recombinant hybrid skin test correctly identified true grass pollen sensitization in children with complex sensitization profiles.	The immunoblot method used for serum specific IgE testing may not be widely available in all clinical settings.	2
Peveri et al. [159]	2019	CS, R, M	To evaluate the clinical and economic impact of molecular allergy diagnostics (component-resolved diagnosis, CRD) in improving diagnosis and guiding treatment of respiratory and food allergies.	Patients (10 months-83 years) with suspected respiratory or food allergy	462	Calculation of improvement in diagnostic accuracy; optimization of the prescription of specific immunotherapy; economic evaluation, including cost savings related to diagnosis and treatment.	Taking into account only the 88 patients who experienced a change in treatment decision, in 51 of them (58%) the prescription of AIT changed from trees to grasses and from trees to cypresses based on the positive response to genuine molecules, while in 42% of cases AIT became the treatment of choice (instead of drugs) in 17 patients (19%), while in 20 patients (23%) the opposite occurred, i.e., drugs were prescribed instead of AIT.	The implementation of CRD brought about a change in diagnosis and therapy, i.e., in the potential prescription and composition of AIT in 88 patients (32%).	In some countries, the sample size is small, which could lead to bias; climate and dietary differences between countries can influence awareness profiles; the study was observational and did not establish causal relationships.	1
Miranda-Machado et al. [131]	2018	CS, R, M	Estimate Positive Skin Reactivity to Various Allergens in Patients Diagnosed with Allergic Conjunctivitis.	Patients (3–74 years) with allergic conjunctivitis.	92	Assess allergen prevalence by skin testing; identify the most common allergens responsible for sensitization; to examine the correlation between skin sensitization and the presence of other associated allergic diseases.	70.65% of patients showed positive reactivity to at least one allergen; the most frequently associated allergic diseases were: allergic rhinitis 51%, asthma 23%, dermatitis 10%.	The majority of patients showed positive skin reactivity to at least one allergen. About one-third of patients with allergic conjunctivitis showed skin reactivity to two or more types of allergens.	Lack of a healthy control group; limited selection of allergens tested in the skin test; possible bias due to subjective assessment of clinical severity	2
Farraia et al. [163]	2023	CS, R, S	Develop and evaluate a personalized predictive algorithm for asthma that integrates allergic sensitization information using component-resolved diagnosis (CRD).	Patients (0–13 years) with allergic conjunctivitis.	1125	Identification of latent components (LCs) associated with asthma, rhinitis and eczema; Development of a predictive model for asthma at 13 years of age; Evaluating Model Accuracy Using Receiver Operating Characteristic (ROC) Curve Statistics.	Among the 112 allergenic components, 32 were not positive in any child at the age of 10 years; thus, 80 components were included in this analysis; sensitivity and specificity reached 75% and 81%, while positive predictive value (VPP) and negative predictive value (VPN) were 28% and 97%, respectively.	Different late-childhood allergic sensitization profiles assessed using component-resolved diagnosis could be useful for discrimination of children with a higher risk of developing asthma.	The algorithm requires further testing on larger and more diverse populations; Clinical outcomes after algorithm-driven AIT were not comprehensively evaluated in this study.	1
Fernandes Polido et al. [138]	2015	CS, R, S	To evaluate correlations between allergen-specific serum IgE levels and the presence of ocular allergies.	Patients (3–63 years old) with a clinical diagnosis of conjunctivitis/allergic keratoconjunctivitis.	61	Identify the main allergens involved in eye allergies; To evaluate the correlation between allergen-specific IgE levels and the presence of eye allergies.	Patients were diagnosed with seasonal allergic conjunctivitis (26.2%) and perennial allergic conjunctivitis (37.7%); the most frequently positive allergens were mixed dust mites (94.9%).	The diagnosis of allergic conjunctivitis is mainly based on the patient’s clinical history and clinical examination. Laboratory tests, such as serum IgE levels, may be useful to confirm the clinical diagnosis.	Limited sample of children; lack of a healthy control group; need for further studies to validate the use of the recombinant hybrid in different pediatric populations.	2
Pesonem et al. [132]	2015	CS, P, S	To evaluate the reproducibility of the skin test and its predictive ability of allergic symptoms up to early adulthood.	Healthy unselected infants were followed prospectively from birth until age 20 years	200	Evaluate the reproducibility of a positive skin test at 5 years through adulthood; determine the predictive ability of the 5-year skin test for allergic symptoms at 11 and 20 years.	Skin positivity at 5 years was fully reproducible at 11 and 20 years (100%), without positive subjects becoming negative at follow-up; skin positivity at 5 years predicted allergic symptoms at 11 years (sensitivity 28%, specificity 94%) and 20 years (sens 28%, spec 91%), but not atopic dermatitis	SPT is a well-established method for assessing immunoglobulin E-mediated sensitization and is widely used in the diagnosis of allergic conditions; however, long-term reproducibility and predictive value are limited.	Lack of a randomized control group.	2
Schoos et al. [89]	2015	CS, P, S	To evaluate the concordance between the measurement of SPT and SIgE in young children to diagnose allergic sensitization.	Children (0–6 years old) born to mother with history of asthma.	389	Prevalence of sensitization to inhalant and food allergens, measured by SPT and sIgE, and degree of agreement between the two methods.	Finding moderate agreement between SPT and sIgE results at ages 1 1/2 years (j = 0.48), 4 years (j = 0.45), and 6 years (j = 0.55) (at 1 1/2 years, agreement was poor (j = 0.0011), but only 7 children had a positive test).	There is substantial disagreement between the level of SPT and sIgE for the diagnosis of allergic sensitization to common inhalant and food allergens in young children, with increasing disagreement with age for food sensitization.	Convenience sampling, which can introduce selection bias; lack of a healthy control group to compare results; limited generalizability of the results to populations other than the one studied.	1 and 2
Mastrorilli et al. [152]	2016	CS, R, M	Classify the different endotypes of PFA in children with SAR according to IgE sensitization to panallergens.	Children (4–18 years) with seasonal allergic rhinoconjunctivitis.	1271	Identification of PFA endotypes by clustering analysis based on IgE sensitization to panallergens.	Three hundred patients (23.6%) (180 males, mean age 10.9 0.4 years (95% CI)) reported experiencing typical PFS manifesting with symptoms limited to the oral cavity (immediate oral itching, with or without angioedema of the lips and/or tongue) following ingestion of at least one plant-based food. Two hundred and twenty-nine of them (73.6%) reported symptoms in the previous 12 months. Nine hundred and seventy-one (685 males, mean age 10.2 0.2 years (95% CI)) reported no PFS; PFS was observed starting in preschool children (26/108 at 5 years of age, 24%) and its frequency increased progressively with age.	Food allergy manifesting as OAS is, in children with pollen allergy, a frequent and complex syndrome that can be classified into five disease endotypes, characterized by sufficiently distinct characteristics	The generalizability of the predictive model to other populations is uncertain; The study did not evaluate the long-term effectiveness of the predictive algorithm.	3
Araujo et al. [90]	2016	CS, R, S	To evaluate sensitization to specific allergenic components in children with allergic respiratory diseases in Brazil using a panel of recombinant molecules to identify sensitization at the molecular level.	Children (6–15 years) with positive SPT for common inhalant allergens.	101	Identification of specific molecular sensitization and their correlation with clinical symptoms and skin tests.	100% of patients were sensitized to one or more inhalant allergens: Der p 1 (74.2%), Der p 2 (73.3%), Der f 1 (74.2%) and Der f 2 (72.3%). Seventy-one (70.3%) were positive at the same time as the four Dermatophagoides allergens. Specific IgE for a third mite species, Euroglyphus maynei, were detected less frequently. Eur m 2 (54.4%) presented all positive tests at Der p 1, Der p 2, Der f 1 and Der f 2. The highest median levels of IgE antibodies were also detected in sera from mite sensitized patients: Der p 1: 26.5; Der p 2: 20.2; Der f 1: 18.5; Der f 2: 30.7.	Virtually all sera reactive to *Dermatophagoides pteronyssinus* also reacted to *Dermatophagoides farinae*, and most reacted to the supply mite Euroglyphus maynei, perhaps due to cross-reactivity.	Lack of a healthy control group to compare results; Possible variability in diagnostic methods and patient inclusion criteria.	3
Lou et al. [133]	2017	CS, R, M	Analyze aeroallergen sensitization patterns in patients with self-reported allergic rhinitis in China and identify effective SPT minimal screening panels in different climate regions of the country.	Subjects (6–75 years old) who self-report allergic rhinitis.	7148	Calculate sensitization rate to different inhalant allergens, identification of minimum allergen panels for SPT, correlation between sensitization and comorbidities.	In total, 1081 patients (17.0%) had one monosensitization, 1567 patients (24.7%) had two sensitizations, 1252 patients (19.7%) had three sensitizations, 631 patients (9.94%) had four sensitizations, and 1819 patients (28.6%) had five or more sensitizations; overall, 2950 patients (47.3% of the 6236 self-reported RA patients in central China) tested positive for Der f were detected by the first panel allergen. In the second phase, 1028 (16.48%) patients sensitized to mugwort were identified among Derf-negative patients, and then 3978 (63.8%) patients with sensitization to Derf or mugwort were confirmed. With this process, after Der p, the eighth panel allergen, to which 5313 (85.2%) patients were sensitized, this accounted for more than 96% (5313/5530) of all sensitized subjects in the Central China group.	Using the same allergen panel as in central China, more than 96% of sensitized subjects in other regions were screened for RA.	The sensitivity of the 5-year skin test in predicting allergic symptoms in adulthood is relatively low; a negative skin test at 5 years does not exclude the possibility of sensitization and allergic symptoms in old age.	2
Gonzalez et al. [156]	2016	CS, R, M	Compare the evolution of allergic sensitization profiles in children with hay fever using skin testing (SPT) and resolved molecular diagnosis (CRD).	Children (2–14 years) with symptoms suggestive of seasonal respiratory allergic diseases confirmed by skin tests.	123	Changes in allergic sensitization profiles detected by SPT and CRD during a 3-year follow-up.	The number of patients who, being monosensitive at the initial visit, became polysensitive at the final visit is significant for both SPT (seven patients, *p* = 0.0106) and CRD (eight patients, *p* = 0.0060). No statistical differences were found between the different age groups.	Emphasized the importance of incorporating CRD in polysensitized patients to allow the distinction between authentic sensitizers and cross-reactivity sensitizations. CRD is also useful when clinical benefit objectives are not achieved at the start of immunotherapy treatment.	Study conducted on a high-risk population, which limits the generalizability of the results; lack of long-term follow-up to assess the evolution of allergic sensitization; possible variations in the laboratory methods used for SPT and sIgE that affect the reliability of the results.	3
Martinez-Canavate et al. [154]	2018	CS, R, M	To evaluate how molecular diagnosis influences the prescription of specific immunotherapy (AIT) in children sensitized to both grass and olive pollen.	Children (5–15 years) with seasonal akkerguca rhinitis and sensitization to various pollens	281	Evaluation of changes in AIT prescribing based on molecular diagnosis; evaluation of the response to AIT in relation to molecular sensitization profiles.	When the researchers received the information on in vitro sensitization patterns, 73.44% said they would change the prescribed AIT. Overall, treatment would have been changed in 52.87% of patients.	Molecular diagnosis is a useful tool in polysensitized patients as it distinguishes between real sensitization and cross-reactions and helps to define the correct composition of AIT.	Cross-selective study, therefore impossible to establish a causal link; lack of long-term follow-up to assess the evolution of endotypes over time; possible selection bias as participants were recruited in a specific geographic area.	3
Vernal conjunctivitis										
Gupta et al. [164]	2024	C, R, M	To analyze the epidemiology and clinical presentation of allergic eye diseases (AEDs) and vernal keratoconjunctivitis (VKC) in Indian children, exploring the influence of environmental factors.	Children (5–12 years) aged 5–15 years, from rural and urban areas of different geographical regions of India.	8231	Prevalence of AED and VKC; association between environmental exposure and incidence of AED/VKC; prevalence of visual impairment in cases of VKC.	AED prevalence: 4.98% (95% CI: 4.51–5.45%). Prevalence of VKC: 1.11% (95% CI: 0.89–1.34%). Most common clinical subtype of VKC: eyelid (82.6%). 11.9% of VKC cases had visual impairment.	Highest incidence of AED among children aged 11–16 years, in urban areas, in lower socioeconomic classes, and in the presence of exposure to: incense smoke, intense sunlight, dust/pollution and in the winter months, higher incidence of VKC in rural areas, with exposure to wind and during spring.	Cross-sectional design limits causal inference. Potential selection and information biases. Absence of longitudinal data to assess the long-term progression of VKC.	1, 2
Dubbaka et al. [168]	2023	CS, R, S	To evaluate the presence of conjunctival perilimbal pigmentation (PLP) in Indian patients with vernal keratoconjunctivitis (VKC).	Pediatric patients (11.4 ± 5.6 years) diagnosed with VKC at a tertiary ophthalmic care center in Western Maharashtra, India.	152	Incidence and characteristics (type, color, extent) of PLP; correlation with severity and duration of VKC.	PLP present in 81 cases (53.29%; 95% CI: 45.03–61.42%; *p* < 0.001). 15 cases (18.5%) had PLPs in all four quadrants. Significant change in the extent of PLP between quadrants (χ^2^ = 73.85, *p* < 0.001).	No correlation between PLP extent and age, sex, disease duration, or PLP type/color.	Cross-sectional design limits causal inference. Single-center study, which may affect the generalizability of results.	1, 2
Ghiglioni et al. [172]	2019	C, P, S	To evaluate whether the control of ocular symptoms in children with severe vernal keratoconjunctivitis (VKC), using cyclosporine or tacrolimus-based eye drops, improves sun exposure and, consequently, serum vitamin D (25OHD) levels.	242 children with active VKC: 94 treated with cyclosporine 1% or tacrolimus 0.1%. 148 with mild VKC, not treated with immunomodulators. Serum vitamin D levels were measured in 71 children (60 with cyclosporine, 11 with tacrolimus), between March and November 2016.	242	Change in serum 25OHD levels before and after treatment.	Median vitamin D levels before treatment were: 23.7 ng/mL in the cyclosporine group, 23.8 ng/mL in the tacrolimus group. After treatment, levels increased to: 32.8 ng/mL (cyclosporine), 32.9 ng/mL (tacrolimus). Before treatment, 33% had levels < 20 ng/mL (deficiency); At the end of the summer, only 4% were still lacking.	Overweight children showed less improvement in vitamin D levels than those with BMI below the 85th percentile. Children who needed local steroid therapy during the summer showed a greater improvement in vitamin D levels than those who did not. Patients with limbal VKC had a significantly higher increase in vitamin D levels than those with tarsal VKC (*p* = 0.04).	Absence of a control group. Lack of detailed information on sun exposure, subjective well-being of patients, and bioavailability of immunomodulatory eye drops.	1, 2
Wang et al. [169]	2020	CS, R, S	To evaluate the distribution of serum specific IgE in children with allergic conjunctivitis and analyze the presence of concomitant allergic diseases.	Children (2 months—6 years) diagnosed with allergic congiountivitis.	310	Serum specific IgE levels for common allergens (e.g., dust mites, pollen, etc.), presence of concomitant allergic diseases (e.g., asthma, allergic rhinitis).	Higher serum specific IgE levels for common allergens have been found in children with allergic conjunctivitis. Concomitant allergic diseases such as asthma and allergic rhinitis were frequently present.		No longitudinal follow-up, limited generalizability of results due to the sample coming from a single center.	1
Artesani et al. [171]	2021	CS, R, S	To assess health-related quality of life (HRQoL) in children diagnosed with vernal keratoconjunctivitis (VKC).	Children (5–12 years) diagnosed with Keratoconjunctivitis Vernal.	70	Health-related quality of life assessed by a standardized questionnaire.	Children with VKC had a significantly reduced quality of life compared to healthy children.		Small sample size, single-center study, subjective nature of quality of life assessment.	1, 2
**Non-original studies**										
**AUTHOR**	**YEAR**	**DESIGN**	**OBJECTIVES**		**POPULATION**	**OUTCOME**	**RESULTS (Quantitative Elements)**		**RESULTS** **(Description)**	**PICO**
Allergic Asthma										
Wang L et al. [17]	2023	Non-systematic review	Discuss the definition and epidemiology of mild asthma and summarize the risk factors and biomarkers of “severe” acute exacerbations in patients with mild asthma to improve understanding of mild asthma and its severe acute exacerbations.		n.a.	n.a.	n.a.		Higher use of SABA is related to a higher risk of exacerbations and death. Risk factors for acute exacerbations and progression of mild asthma include genetic factors, disease pathology, but also abnormal lung function, increased inflammatory cells, and also inappropriate use of SABA and ICS.	1
Irving G et al. [39]	2017	Without meta-analysis	Describe the average length of GP visits in economically developed countries and low/middle-income countries and examine the relationship between the length of visits and organizational economic and health outcomes.		Adults	//	The average length of the visit varied around the world, ranging from 48 s in Bangladesh to 22.5 min in Sweden. 18 countries, or about 50% of the world’s population, spend 5 min or less on their GPs.		The duration of visits varies internationally and a large percentage of the world’s population has only a few minutes with their general practitioner.	1
Manti S et al. [24]	2021	Without meta-analysis	To provide recommendations for the diagnosis and therapeutic management of asthma exacerbations in the pediatric population.		Pediatric	//	//		Risk factors for asthma exacerbations include: hospital admissions or emergency room visits for asthma in the past year, excessive use of inhaled bronchodilators, and incorrect inhalation technique.	1
O’Connor A et al. [40]	2025	With meta-analysis	To evaluate the effects of asthma education interventions, delivered at home to children, their caregivers, or both, on asthma-related outcomes.		Children and adolescents (2–18 years) with asthma	The primary outcomes were exacerbations that led to emergency department visits and exacerbations that required a course of oral corticosteroids. Six months was the primary time point for the outcomes.	Homeschooling may result in little or no difference in exacerbations leading to emergency department visits at six-month follow-up compared to control, but the evidence is very uncertain (Peto OR 1.22, 95% CI 0.50 to 2.94; 5 studies (2 studies with 2 intervention arms), 855 participants; very low certainty trials). Homeschooling results in little to no difference in exacerbations requiring a course of oral corticosteroids compared to control (mean difference (MD) −0.18, 95% CI −0.63 to 0.26; 1 study (2 intervention arms), 250 participants; low certainty trials). Homeschooling can improve quality of life scores compared to control, but the evidence is very uncertain (standardized mean difference (SMD) 0.32, 95% CI 0.08 to 0.56; 4 studies, 987 participants; very low certainty trials). The evidence is very uncertain about the effects of homeschooling on the average of symptom-free days, days missed from school/work, and exacerbations leading to hospitalization compared to control (all evidence of very low certainty). A more intensive homeschooling intervention did not reduce exacerbations that led to emergency department visits (Peto OR 1.36, 95% CI 0.35 to 5.30; 4 studies, 729 participants; low certainty trials) or exacerbations that required a course of oral corticosteroids (MD 0.08, 95% CI −0.14 to 0.30; 3 studies, 605 participants; evidence of low certainty), compared to a less intensive type of homeschooling. A more intensive at-home asthma education intervention may reduce hospital admissions due to an asthma exacerbation (Peto OR 0.14, 95% CI 0.04 to 0.55; 4 studies, 689 participants; low-certainty trials), but not days missed from school (low-certainty trials), compared to a less intensive home-based asthma education intervention.		There is uncertain evidence in favor of home-based educational interventions for asthma compared to traditional care, out-of-home education or a less intensive educational intervention. Homeschooling can improve quality of life compared to control and reduce the risk of hospitalization compared to a less intensive educational intervention.	1
Osama H et al. [41]	2024	With meta-analysis	Investigate the impact of different interventions on asthmatic patients’ adherence to control inhalers.		Pediatric population (6–12 years)	Interventions in the selected studies included asthma-focused educational sessions, SMS reminders and technology-based feedback.	The overall effectiveness of the interventions compared to the control group produced a statistically significant but small effect (SMD 0.44, 95% CI 0.24–0.63, *p* < 0.001).		Adherence promotion interventions have been shown to be effective among patients with asthma.	1
Fainardi V et al. [51]	2020	Non-systematic review	Describe a 6-step approach to the diagnosis and management of the child with severe asthma problem.		Pediatric	//	//		More than 85% of children with severe asthma are atopic, have a positive family history of asthma, and show some degree of eosinophilic inflammation with multiple sensitization to aeroallergens. Skin prick tests for aeroallergens, food and molds, tests for serum total IgE and tests for specific IgE are performed to determine the atopic status. The basic elements should be evaluated: alternative diagnoses, comorbidities, adherence to treatment and inhalation techniques.	2, 3
Roberto G et al. [50]	2024	Non-systematic review	Explore the evolving landscape of pediatric asthma and rhinitis, focusing on the identification and characterization of different subtypes.		Pediatric	//	//		Recognizing endotypes, such as eosinophil, neutrophil, pauci-granulocytic asthma, and mixed granulocytic asthma, provides molecular insight into the underlying mechanisms, paving the way for personalized treatment strategies.	3
Fainardi V et al. [49]	2022	Non-systematic review	Discuss the main phenotypes and endotypes of asthma recognized in children.		Pediatric	//	//		A deep knowledge of the patient’s phenotype and endotype can guide a personalized therapeutic approach.	3
Licari A et al. [48]	2018	Non-systematic review	To provide an up-to-date overview of asthma endotyping, focusing primarily on novel non-invasive biomarkers in childhood asthma.		Pediatric	n.a.	n.a.		Careful evaluation of inflammatory endotypes should be considered a central component of the diagnostic workup and management of severe asthma in children. Currently, a limited number of biomarkers are available in pediatric clinical practice, including blood and sputum eosinophils, serum IgE, periostin, and FeNO, which primarily reflect several molecular components of type 2 high airway inflammation. Individually or in combination, they can help improve diagnosis and predict disease severity and response to both conventional and innovative targeted biologic therapies.	3
Rhinoconjunctivitis										
Knyziak-Mędrzycka et al. [162]	2023	Systematic review with meta-analysis	Exploring the role of precision molecular diagnosis of allergies (PAMD) in predicting the development of atopy and planning allergen-specific immunotherapy in children.		Children and adolescents with suspected or confirmed atopy	Predict the development of atopy; Plan allergen-specific immunotherapy Monitor the “Allergen Brand”.	The cut-off levels of sIgE associated with clinically significant peanut allergy were ≥5 kU/L in children ≤2 years of age and ≥14 kU/L in those older than 2 years; Bet v 1 > Mal d1 > Cor a 1.04 > Ara h 8 > Pru p 1 > Aln g 1 > Api g 1 > Act d 8 > Gly m 4.		Elevated levels of allergen-specific IgE such as Gal d1 and Bet v1 are predictive of persistent allergies and progression to respiratory disease; PAMD enables more targeted patient selection for allergen-specific immunotherapy, improving treatment efficacy.	1
Nevis et al. [135]	2016	Systematic review with meta-analysis	To evaluate the diagnostic accuracy of SPT for allergic rhinitis, using nasal provocation as the gold standard.		Patients with allergic rhinitis	430	Calculate Sensitivity and Specificity of SPT in the Diagnosis of Allergic Rhinitis.		Average SPT sensitivity: 85%; Average SPT specificity: 77%.	2
Vernal Conjunctivitis										
Rasmussen et al. [166]	2023	Systematic review with meta-analysis	Determine the prevalence of allergic sensitization in patients with vernal keratoconjunctivitis (VKC) and provide an overview of published studies on this topic.		33 eligible studies with a total of 2122 patients with VKC	Prevalence of allergic sensitization and types of allergens involved.	The combined prevalence of allergic sensitization in patients with VKC was 57.7% (95% CI: 52.5–62.8%).		The most frequently positive allergy tests were those for inhalant allergens, in particular house dust mites and pollen. Subgroup analyses showed: Conjunctival provocation test: 51.4%, total IgE in tears: 68.7%, specific IgE in tears: 58.9%, Skin Prick test: 58.2%, Serum specific IgE: 54.2%.	**2**
**Guidelines**										
**AUTHOR**	**YEAR**	**DOCUMENT TYPE**	**OBJECTIVES**				**ELEMENTS OF INTEREST**			**PICO**
Allergic Asthma										
Lougheed MD et al. [23]	2010	Consensus document	Integrate the new evidence into the Canadian continuum diagram for asthma management, encompassing both pediatric and adult asthma.				Risk factors for asthma exacerbations are: inadequate inhalation technique, underuse or poor adherence to treatment, and discontinuous medical care.			1
Asher I et al. [19]	2017	Guidelines	To provide simple, practical, evidence-based recommendations for the diagnosis, assessment, and management of asthma in children and adolescents in New Zealand, with the goal of improving outcomes and reducing inequalities.				Risk factors for asthma exacerbations are: excessive use of inhaled bronchodilators, incorrect inhalation technique, underuse or poor adherence to treatment, and discontinuous medical care.			1
National Institutes of Health—2020 Focused updates to the Asthma Management Guidelines [178]	2020	Guidelines	Overseeing the development of national asthma guidelines.				Clinical assessment of asthma control should be performed by medical history, validated asthma control instruments and, when appropriate, pulmonary function tests.			2
Matsunaga K et al. [58]	2025	Guidelines	Provide evidence for the interpretation of type 2 inflammation measurements and promote the widespread use of inflammation assessment to further improve airway efficiency and respiratory diseases.				SIgE has good significance as an indicator of inflammation (also SPT).			3
Gaillard et al. [66]	2021	Guidelines	Develop evidence-based clinical practice guidelines for asthma diagnosis using nine PICO questions.				The working group recommends spirometry, bronchodilator reversibility testing and FeNO measurement as first-line diagnostic tests in children undergoing asthma investigation. The working group advises against diagnosing asthma in children based only on clinical history or a single abnormal objective test.			4
Chung et al. [179]	2014	Guidelines	The guidelines reviewed the definition of severe asthma and provided recommendations and guidelines on the assessment and treatment of severe asthma in children and adults.				The guidelines recommend the use of FeNO for the diagnosis of asthma in children aged 5–16 years, with a suggested cut-off value of 25 ppb. However, FeNO should be interpreted as part of a broader clinical evaluation, considering the potential for overtreatment resulting from false positive results.			5
Global Strategy for Asthma Management and Prevention (2025 Update) [2]	2025	Guidelines	This report aims to provide an evidence-based asthma management strategy for healthcare professionals and policymakers.				For most patients, asthma can be managed in general practice, but some clinical situations require expert advice regarding diagnosis and/or management. One such case is persistent or severely uncontrolled asthma or frequent exacerbations. - Atopic status can be identified by SPT or sIgE. The presence of a positive SPT or positive sIgE does not mean that the allergen is causing symptoms. The relevance of exposure to the allergen and its relationship to symptoms should be confirmed by the patient’s medical history. - One or more positive tests for a clinically relevant allergen-specific IgE (or SPT) in a patient with a confirmed diagnosis of asthma is consistent with allergic asthma, if consistent with a history of symptoms triggered by exposure to a specific aeroallergen.			1, 2, 3, 4, 5
Asthma: diagnosis, monitoring and chronic asthma management (BTS, NICE, SIGN) guidelines [25]	2024	Guidelines	These guidelines cover the diagnosis, monitoring and management of asthma in adults, young people and children. Their goal is to improve the accuracy of diagnosis, help people control their asthma, and reduce the risk of asthma attacks.				For children aged 5–16 years, if there are still diagnostic doubts after performing other tests (such as FeNO, spirometry, BDR, PEF variability, SPT for house dust mites, total IgE levels and blood eosinophil counts), the committee agreed that the diagnosis should be sent to an asthma specialist for a second opinion, including the evaluation of a provocation test. With regard to children aged 5 to 16 years with a clinical history suggestive of asthma, both the skin prick test for house dust mite sensitization and the measurement of total IgE are sensitive tests, and the committee agreed that one or the other should be performed thereafter. If the test is negative, asthma is highly unlikely and can be ruled out without resorting to the bronchial provocation test. Although blood sampling for IgE is invasive, it has the advantage of also allowing an eosinophil count, and if this is greater than 0.5 × 109 per liter, it would support a diagnosis of asthma.			1, 2, 3, 4, 5
Allergic conjunctivitis										
Roberts G et al. [3]	2018	To provide evidence-based clinical recommendations for the use of AIT in allergic rhinoconjunctivitis, considering both seasonal and perennial forms.	Patients of all ages with allergic rhinoconjunctivitis, with or without asthma.				Reduction in symptoms of allergic rhinoconjunctivitis; reduction in the use of symptomatic drugs; improved quality of life (QoL); persistence of efficacy after the end of treatment (long-term effect); safety and tolerability of AIT; prevention of progression to allergic asthma (secondary but relevant endpoint).			1

**Table A3:** Quality appraisal of original studies on allergic asthma included in the present analysis according to the Newcastle Ottawa Scale (NOS). For detailed description of included item, please refer to Wells G et al. [8].

	YEAR	PICO	D1	D2	D3	D4	D5	D6	D7	D8	D9	SCORE
Hansen et al. [22]	2023	1	+1	+1	+1	0	0	0	0	+1	0	4
Nwaru et al. [18]	2019	1	+1	+1	+1	+1	+1	+1	+1	+1	0	8
Hakizimana et al. [27]	2024	1	+1	0	+1	+1	0	0	+1	+1	+1	6
Paskaradevan et al. [26]	2022	1	+1	+1	+1	+1	+1	0	+1	0	0	6
Rosman et al. [28]	2021	1	+1	+1	+1	0	+1	0	+1	+1	+1	7
Berdach et al.	2022	1	+1	+1	+1	+1	+1	+1	+1	+1	+1	9
Bender et al. [31]	2000	1	+1	+1	+1	+1	+1	+1	+1	+1	+1	9
Butz et al. [30]	2015	1	+1	+1	+1	+1	+1	+1	+1	+1	+1	9
Erickson et al. [37]	2005	1	+1	+1	+1	+1	+1	+1	+1	+1	0	8
Frey et al. [36]	2016	1	+1	+1	+1	+1	+1	+1	+1	+1	+1	9
Beck et al. [35]	2015	1, 3	+1	+1	+1	+1	+1	+1	+1	+1	0	8
Diette et al. [33]	2011	1	+1	+1	+1	+1	+1	+1	+1	+1	0	8
Yee et al. [38]	2013	1	+1	+1	+1	+1	+1	+1	+1	+1	0	8
Hauersley et al. [44]	2022	2	+1	+1	+1	+1	+1	0	+1	0	0	6
Kallis et al. [43]	2023	2	+1	0	+1	+1	+1	0	+1	+1	0	6
Keemink et al. [46]	2015	2	+1	0	+1	+1	0	0	+1	+1	0	5
Koole et al. [45]	2020	2	+1	0	+1	+1	0	0	+1	0	0	4
Nyenhuis et al. [47]	2020	2	+1	0	+1	+1	+1	0	+1	0	0	5
Håkansson et al. [59]	2024	3	+1	+1	+1	+1	+1	+1	+1	+1	0	8
De Jong et al. [62]	2020	3	+1	0	+1	+1	+1	0	+1	0	0	5
Lehmann et al. [60]	2017	3	+1	0	+1	+1	+1	0	+1	0	0	5
Castanhinha et al. [61]	2015	3	+1	0	+1	+1	+1	0	+1	0	0	5
Mingotti et al. [70]	2020	4	+1	0	+1	+1	+1	0	+1	0	0	5
Yang et al. [67]	2017	4	+1	+1	+1	+1	+1	0	+1	0	0	6
Tran et al. [71]	2024	4	+1	+1	+1	+1	+1	0	+1	+1	0	7
Yimlamai et al. [72]	2024	4	+1	+1	+1	+1	+1	0	+1	+1	+1	8
Barański et al. [175]	2022	4	+1	+1	+1	+1	+1	0	+1	0	0	6
Lo et al. (a) [80]	2020	4	+1	+1	+1	+1	0	0	+1	0	0	5
Lo et al. (b) [81]	2020	4	+1	+1	+1	+1	0	0	+1	0	0	5
Chen et al. [82]	2023	4	+1	+1	+1	+1	+1	+1	+1	+1	+1	9
Zhu et al. [87]	2019	4	+1	+1	+1	+1	+1	0	+1	0	0	6
Dabbaghzadeh et al. [88]	2019	4	+1	+1	+1	+1	+1	0	+1	0	0	6
Yin et al. [86]	2017	4	+1	+1	+1	+1	+1	0	+1	0	0	6
Eom et al. [69]	2020	4	+1	0	+1	0	0	0	+1	0	0	3

**Table A4:** Appraisal of the risk of bias of randomized controlled trials included in the analyses according to the National Toxicology Program (NTP)’s Office of Health Assessment and Translation (OHAT) handbook and respective risk of bias (ROB) tool. Note: D1: possibility of selection bias; D2: exposure assessment; D3: outcome assessment; D4: confounding factors; D5: reporting bias; D6: other bias; ☹☹: definitively high; ☹: probably high; ☺: probably low; ☺☺: definitively low.

ASTHMA	D1	D2	D3	D4	D5	D6
Sheares et al. 2015 [34]	☺	☺☺	☺☺	☺☺	☺☺	☺
Boonjindasup et al. 2023 [73]	☺	☹	☹	☹ ☹	☹	☹
Gao et al. 2018 [85]	☺	☹	☹ ☹	☹	☺	☺

**Table A5:** Quality appraisal of original studies on rhinoconjunctivitis included in the present analysis according to the Newcastle Ottawa Scale (NOS). For detailed description of included item, please refer to Wells G et al. [8].

	YEAR	PICO	D1	D2	D3	D4	D5	D6	D7	D8	D9	SCORE
Agenas et al. [103]	2023	1	+1	0	0	+1	+1	+1	+1	+1	0	6
Kiotseridis et al. [176]	2018	1	+1	0	+1	+1	+1	0	+1	+1	0	6
Yang et al. [102]	2018	1	0	0	+1	+1	+1	+1	+1	0	0	5
Paoletti et al. [96]	2025	1	+1	0	+1	+1	+1	0	0	0	+1	5
Nyiraneza et al. [95]	2020	1	+1	0	+1	+1	+1	0	+1	0	+1	6
Klangkalya et al. [94]	2024	1	+1	0	+1	+1	+1	+1	+1	0	+1	7
Alimuddin et al. [177]	2018	1	+1	0	+1	+1	+1	0	+1	0	+1	6
Alessandri et al. [55]	2018	1	+1	0	+1	+1	+1	0	+1	+1	+1	7
Masrur et al. [129]	2020	2	+1	0	+1	+1	+1	0	+1	+1	+1	7
Arasi et al. [160]	2021	2	+1	0	+1	+1	+1	+1	+1	+1	+1	8
Douladiris et al. [130]	2019	2	+1	0	+1	+1	+1	0	+1	0	+1	6
Peveri et al. [159]	2019	1	+1	0	+1	+1	+1	+1	+1	+1	+1	8
Miranda Machado et al. [131]	2018	2	0	0	+1	+1	+1	+1	+1	0	0	5
Farraia et al. [163]	2023	1	+1	0	+1	+1	+1	+1	+1	+1	+1	8
Fernandes Polido et al. [138]	2015	2	+1	0	+1	+1	+1	+1	+1	+1	0	7
Pesonem et al. [132]	2015	2	+1	0	+1	+1	+1	+1	+1	+1	+1	8
Schoos et al. [89]	2015	1, 2	+1	0	+1	+1	+1	+1	+1	+1	+1	8
Mastrorilli et al. [153]	2016	3	+1	0	+1	+1	+1	+1	+1	0	+1	7
Araujo et al. [90]	2016	3	+1	0	+1	+1	+1	0	+1	0	+1	6
Lou et al. [113]	2018	2	+1	0	0	+1	0	+1	0	+1	+1	5
Gonzalez-Mancebo et al. [156]	2016	3	+1	0	+1	+1	+1	0	+1	+1	+1	7
Martinez Canavate et al. [154]	2018	3	+1	0	+1	+1	+1	+1	+1	+1	+1	8
Del Rio et al. [155]	2018	1	+1	0	+1	+1	+1	0	+1	+1	+1	7

**Table A6:** Appraisal of the risk of bias of randomized controlled trials on allergic rhinoconjunctivitis included in the analyses according to the National Toxicology Program (NTP)’s Office of Health Assessment and Translation (OHAT) handbook and respective risk of bias (ROB) tool. Note: D1: possibility of selection bias; D2: exposure assessment; D3: outcome assessment; D4: confounding factors; D5: reporting bias; D6: other bias; ☹ ☹: definitively high; ☹: probably high; ☺: probably low; ☺☺: definitively low.

Allergic Rhinoconjunctivitis	D1	D2	D3	D4	D5	D6
Nolte et al. 2016 [117]	☺☺	☺☺	☺☺	☺	☺☺	☺☺
Okamoto et al., 2019 [116]	☺☺	☺☺	☺☺	☺☺	☺☺	☺☺
Lin et al., 2016 [112]	☺☺	☺☺	☹	☹	☺	☹
Schuster et al., 2025 [111]	☺☺	☺☺	☺☺	☺☺	☺☺	☺☺
Caffarelli et al., 2000 [108]	☺☺	☹	☺☺	☹	☹	☹
Macedo et al., 2025 [104]	☺☺	☺☺	☺☺	☺☺	☺☺	☺☺

**Table A7:** Quality appraisal of original studies on vernal keratoconjunctivitis included in the present analysis according to the Newcastle Ottawa Scale (NOS). For detailed description of included item, please refer to Wells G et al. [8].

	YEAR	PICO	D1	D2	D3	D4	D5	D6	D7	D8	D9	SCORE
Wang et al. [169]	2020	1	+1	0	0	+1	+1	0	0	0	0	4
Artesani et al. [171]	2021	1, 2	+1	0	0	+1	0	0	0	+1	0	3
Gupta et al. [164]	2024	1, 2	+1	+1	0	0	+1	+1	0	+1	+1	6
Dubbaka et al. [168]	2023	1, 2	+1	+1	0	0	0	0	0	0	0	2
Ghiglioni et al. [172]	2019	1, 2	+1	+1	+1	+1	+1	+1	+1	+1	+1	9
